# Metal‐Free Scission of the NO^+^ Triple Bond

**DOI:** 10.1002/anie.202524527

**Published:** 2026-02-15

**Authors:** Julie Willrett, Harald Scherer, Burkhard Butschke, Ingo Krossing

**Affiliations:** ^1^ Institut Für Anorganische und Analytische Chemie and Freiburger Materialforschungszentrum (FMF) Albert‐Ludwigs‐Universität Freiburg Freiburg Germany

**Keywords:** bond activation, nitrosonium cation, NMR spectroscopy, reaction mechanisms, quantum chemical calculations

## Abstract

We report the facile and direct room temperature unimolecular scission of the N≡O^+^ triple bond, one of the strongest known chemical bonds with a bond dissociation energy of 1049 kJ mol^−1^. The reaction of NO[Al(OR^F^)_4_] (R^F^ = C(CF_3_)_3_) and PNP*^t^* ^Bu^ (2,6‐bis(di‐*tert*‐butylphosphinomethyl)pyridine) in CH_2_Cl_2_ yields the 1,2,3‐diazaphospholo[1,5‐*a*]pyridinium derivative [DAPP*^t^* ^Bu^]^+^[Al(OR^F^)_4_]^–^ (**1**), featuring the complete cleavage of the N≡O^+^ bond. At −30°C, the reaction intermediate [PNOP*^t^* ^Bu^]^+^[Al(OR^F^)_4_]^–^ (**2**), which contains the novel bridging P═N−O−P^+^ motif, can be isolated. Mechanistic insights were gained through quantum chemical calculations, which elucidated the formation pathways of **1** and **2**, while NMR spectroscopic kinetic studies quantified the conversion rate from **2** to **1**.

## Graphical abstract

Breaking bonds: The reaction between NO[Al(OR^F^)_4_] (R^F^ = C(CF_3_)_3_) and the diphosphane PNP*^t^* ^Bu^ (2,6‐bis(di‐*tert*‐butylphosphino‐methyl)pyridine) leads to the direct and complete cleavage of the NO^+^ triple bond, one of the strongest chemical bonds known. The mechanism of this reaction was studied using quantum chemical calculations and NMR spectroscopic kinetics investigations.

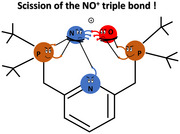

## 1 Introduction

The nitrosonium ion NO^+^ is a versatile and widely used reagent in synthetic chemistry [1, 2], most commonly employed as an oxidant in organic [3, 4, 5, 6, 7] or organometallic [8, 9, 10] synthesis. Moreover, it can be used to synthesize transition metal nitrosyl complexes [11, 12, 13, 14] and readily forms Wheland‐type σ‐complexes with most arenes [15, 16, 17, 18]. Besides its synthetic use, NO^+^ is of fundamental interest as it is isoelectronic to the diatomic molecules CO, N_2_, CN^−^, and C_2_^2−^ [19]. Upon deelectronation [20] of neutral nitric oxide NO (bond order: 2.5), the removal of an electron from an antibonding π* molecular orbital increases the bond order to 3 in NO^+^. This transformation is accompanied by a substantial increase of the bond dissociation energy^1^ from about 630 kJ mol^−1^ in NO [8] to 1049 kJ mol^−1^ in NO^+^ [21], which makes the N≡O^+^ triple bond considerably stronger than that in N_2_ (about 948 ± 6 kJ mol^−1^) [21, 22], while being slightly weaker than the triple bond in CO (1074 ± 4 kJ mol^−1^) [21, 22].

The high oxidizing power of NO^+^ salts is caused by both the relatively high ionization energy of neutral NO (*IE*_NO_ = 9.26 eV) [23] and by the thermodynamic driving force provided by liberation of gaseous NO_(g)_ during redox processes. Yet, the effective redox potential of NO^+^ salts in solution is highly dependent on the employed counteranion [24] and the solvent (levelling effect) [16]. Reported potentials versus the ferrocenium/ferrocene (Fc^+^/Fc) couple span a broad range from +0.56 V (DMF) [25] in strongly interacting solvents over +0.87 V (MeCN) [16] to very high potentials in weakly coordinating solvents, for example, +1.40 V in DCM [16] and +1.52 V in 1,2,3,4‐tetrafluorobenzene (4FB) [16]. The highest reported potential of NO^+^ of +2.34 V is reached in the absence of any solvent and in the solid state when paired with the very weakly coordinating anion (WCA) [F{Al(OR^F^)_3_}_2_]^−^ (R^F^ = C(CF_3_)_3_) [24]. To circumvent this strong solvent dependency of NO^+^ and its propensity to engage in undesired side reactions, a wide variety of selective deelectronators were developed in our group over the last years [24, 26, 27]. These deelectronators simply remove one electron from a substrate without further reactivity, that is, they are single electron oxidants that only remove an e^–^ from the substrate without further bond‐breaking and making. Additionally, their potentials are very high and range from +1.29 to +2.00 V versus Fc^+^/Fc, and are almost independent from the solvent [24, 26]. Typically, these deelectronators are halogenated arene radical cations, which are easily accessible from their neutral precursors via deelectronation by NO[WCA], Ag[WCA], or mixed Ag[WCA]/0.5 X_2_ (X = Br and I) systems [24].

## 2 Background of this Work

**SCHEME 1:**
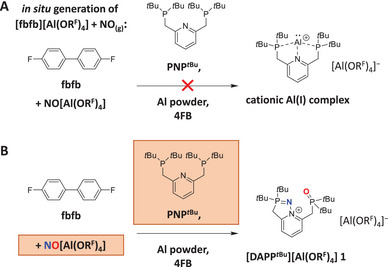
(A) Attempted synthesis of a low‐valent aluminum complex using in situ generated [fbfb][Al(OR^F^)_4_] as deelectronator. (B) Observed NO^+^ activation product [DAPP^*t*Bu^][Al(OR^F^)_4_] (**1**), formed from residual NO[Al(OR^F^)_4_] in the reaction mixture.

A longstanding objective in our group and one potential field of application for the deelectronators has been the synthesis of cationic aluminum(I) complexes from elemental aluminum. After multiple futile attempts to synthesize cationic, low‐valent aluminum complexes with various ligand systems and deelectronators [28], we finally succeeded in synthesizing the low‐valent aluminum(I) cluster cation [Al(AlCp*)_3_]^+^[Al(OR^F^)_4_]^–^ (Cp* = [C_5_Me_5_]^−^) [29], though through a metathesis reaction, not via an oxidative pathway. Only recently, the novel deelectronator [fbfb]^+^[Al(OR^F^)_4_]^–^ (fbfb = 4,4’‐difluorobiphenyl) was developed in our group [30]. Due to its facile preparation and the high redox potential of +1.53 V versus Fc^+^/Fc, we decided to test its reactivity with aluminum powder in the presence of the pincer ligand PNP*^t^* ^Bu^ (PNP*^t^* ^Bu^ = 2,6‐bis(di‐*tert*‐butylphosphinomethyl)pyridine). PNP*^t^* ^Bu^ is a potentially interesting ligand for low‐valent aluminum chemistry (Scheme 1), combining both soft (phosphorus) and hard (nitrogen) donor sites, which could stabilize both Al(I) and Al(III) oxidation states and thus potentially enabling transition metal like catalytic activity at aluminum [31, 32, 33, 34, 35, 36].

## 3 Accidental Reaction of PNP^*t*Bu^ With NO^+^

In orienting work, [fbfb]^+^[Al(OR^F^)_4_]^–^ was prepared in situ by reacting neutral fbfb with NO[Al(OR^F^)_4_] in 4FB, followed by three freeze‐pump‐thaw cycles to remove the NO_(g)_ formed and achieve complete conversion. The resulting dark blue solution was added without further purification to a mixture of PNP*^t^* ^Bu^ and aluminum powder, resulting in an immediate color change to orange. After filtration, layering with *n*‐pentane afforded few colorless crystals inside a dark oil. The crystals were analyzed by single crystal X‐ray diffractometry (scXRD). Strikingly, the obtained structure did not contain any aluminum (as following Scheme 1A), but instead revealed the intriguing N≡O^+^ activation product [DAPP*^t^* ^Bu^][Al(OR^F^)_4_] (**1**, DAPP = **d**i**a**za**p**hospholo**p**yridinium, Scheme 1B). Apparently, the reaction between NO[Al(OR^F^)_4_] and fbfb was not complete, leaving traces of NO^+^ present in the reaction mixture, which reacted with PNP*^t^* ^Bu^ under direct cleavage of the N≡O^+^ triple bond between the two P‐donor atoms.

## 4 Direct Synthesis of **1**

The synthesis of **1** can be easily reproduced by directly reacting NO[Al(OR^F^)_4_] with PNP*^t^* ^Bu^ in 4FB or DCM as a solvent (Equation 1), proving that neither fbfb nor aluminum are required. However, the reaction is always accompanied by the formation of multiple side products. Crystallization of the reaction mixture allows for an efficient purification as **1** crystallizes exclusively out of the product mixture, but the crystallization always results in the formation of dark oil sticking to the crystals. Hence, only low yields ranging from 10% to 15% were obtained for **1** after crystallization.

(1) $$\mathrm{NO}\left[\mathrm{Al}{\left(OR^{F}\right)}_{4}\right]+\mathrm{PN}P^{t\mathrm{Bu}}\overset{4\mathrm{FB}\;\;\;\mathrm{or}\;\;\mathrm{DCM}}{\underset{\mathrm{RT}}{\to }}\begin{array}{c} \left[\mathrm{DAP}P^{t\mathrm{Bu}}\right]\left[\mathrm{Al}{\left(OR^{F}\right)}_{4}\right]\\ 1\;\;\;\;\;(15\%) \end{array}$$

## 5 Molecular Structure of **1**

**FIGURE 1:**
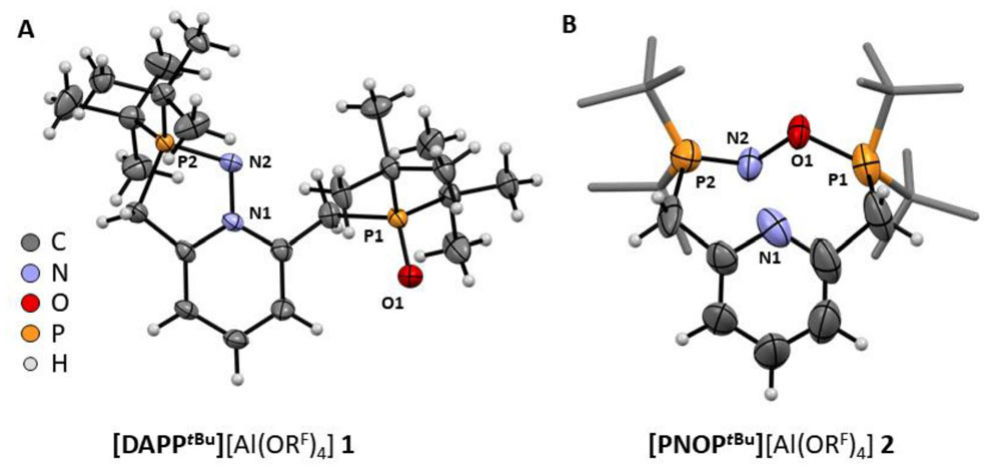
(A) Molecular structure of the cationic moiety of [DAPP^*t*Bu^][Al(OR^F^)_4_] (**1**, P2_1_/c, R_1_ = 7.41%, wR_2_ = 20.08%). Thermal ellipsoids are shown at the 50% probability level. Selected bond lengths: N1−N2 1.393(5) Å, N2−P2 1.644(4) Å, and P1−O1 1.476(3) Å. Computed bond lengths (r^2^SCAN‐3c/def2‐mTZVPP): N1−N2 1.375 Å, N2−P2 1.654 Å, and P1−O1 1.504 Å. (B) Molecular structure of the cationic moiety of [PNOP^*t*Bu^][Al(OR^F^)_4_] (**2**). The structure of **2** refined only to poor crystallographic agreement factors (P2_1_, R_1_ = 12.0%, wR_2_ = 34.7%), yet the connectivity could be determined unambiguously. For a discussion see Section S5. Thermal ellipsoids are shown at the 50% probability level. Disorder of the (NO) moiety not shown and *t*Bu groups represented in capped sticks style with omitted hydrogen atoms for clarity. Computed bond lengths (r^2^SCAN‐3c/def2‐mTZVPP) for comparison: N2−P2 1.651 Å, N2−O1 1.517 Å, and P1−O1 1.601 Å. Full molecular structures of **1** and **2** with anions and disorders included are shown in the Figures S34 and S35.

Figure 1A illustrates the molecular structure of **1**, which features a phosphane oxide moiety (P═O bond: 1.476(3) Å) formed by oxidation of one of the *tert*‐butyl phosphane groups by the NO^+^ oxygen atom. The nitrogen atom from NO^+^ bridges the other *tert*‐butyl phosphane group and the pyridine nitrogen atom, forming a planar five membered 1,2,3‐diazaphospholium moiety. A benzannulated structural motive as in **1**, which can be described as a 1,2,3‐diazaphospholo[1,5‐*a*]pyridinium, however, was hitherto unknown. The N1−N2 bond length (1.393(5) Å) is consistent with an N−N single bond (avg. 1.401 Å) [37] rather than an N = N double bond (avg. 1.240 Å) [37], resembling that in structurally similar phosphanimines (R’*_x_* N^+^−N = PR_3_, 1.372(1) Å for R = Cy, 1.383(2) Å for R = Ph) [38]. The P2═N2 bond in **1** is best described as a double bond to properly reflect the valences of both N1 and N2. However, this bond is slightly elongated (1.644(4) Å) compared to the corresponding P═N double bonds in the acyclic phosphanimines (1.600(1)–1.6130(6) Å) [38]. This bond elongation is likely caused by the ring strain inherent to the cyclic structure in **1** as well as by the high steric demand of the *tert*‐butyl groups.

Reaction (1) represents a very rare direct and metal‐free scission of the N≡O^+^ triple bond. Previously, mostly transition metals were shown to perform this kind of reactivity in (bridged) nitrosyl complexes and often at elevated temperatures [39, 40, 41, 42]. In transition‐metal systems, nitrosyl ligands exhibit non‐innocent behavior, with ambiguous charge distribution, bond orders, and activation degrees [43, 44, 45, 46]. Thus, the cleavage of a non‐activated NO^+^ triple bond without metallic participation and at room temperature (RT) is even more remarkable. While earlier this year, the scission of the NO^+^ triple bond by a frustrated Lewis pair (FLP) decorated with a redox‐active phenothiazine moiety was reported [47], the latter process involves a two‐step initiation (1. electronation of NO^+^ to neutral NO by the phenothiazine moiety, 2. bond scission of neutral NO at the FLP site), and requires a sacrificial solvent molecule as a hydrogen atom donor [47]. In the reaction reported here, the NO bond activation process is entirely intramolecular (after the initial encounter of the reactants) and does not require any further reagents. The mechanistic evidence for this claim is provided in the following.

## 6 Initial NMR‐Monitoring and Synthesis of **2**

To better understand Reaction (1) and its side products, and in order to optimize the yield of **1**, we conducted NMR monitoring of the synthesis of **1**. The reaction mixture was frozen upon addition of the NMR solvent CD_2_Cl_2_, thawed to RT and immediately measured by ^1^H and ^31^P{^1^H} NMR spectroscopy. These experiments revealed that both **1** and the side products form quantitatively within minutes at RT. In an attempt to avoid side‐product formation, we performed the reaction at −30°C throughout. Surprisingly, crystallization under these conditions yielded not the expected structure of **1** but that of a cyclization product of NO^+^ with the PNP*^t^* ^Bu^ ligand where NO^+^ bridges both phosphorus atoms (Figure 1B and Equation 2).

(2) $$\mathrm{NO}\left[\mathrm{Al}{\left(OR^{F}\right)}_{4}\right]+\mathrm{PN}P^{t\mathrm{Bu}}\overset{\mathrm{DCM}}{\underset{-30^{\circ }C}{\to }}\;\;\begin{array}{c} \left[\mathrm{PNO}P^{t\mathrm{Bu}}\right]\left[\mathrm{Al}{\left(OR^{F}\right)}_{4}\right]\\ 2 \end{array}$$

With the intriguing novel P═N−O−P^+^ bridging motif we chose to name this unexpected compound [PNOP*^t^* ^Bu^]^+^[Al(OR^F^)_4_]^–^ (**2**). Unfortunately, the crystals of **2** only diffracted very poorly, and we were only able to measure crystallographic data of inferior quality (*R*_1_ = 12.0%, *wR*_2_ = 34.7%, for more information see Section S5) and thus refrain from discussing bond lengths in the crystal structure of **2**. Yet, the connectivity of **2** could be determined without any doubt, and repeated reactions according to Equation (2) reliably yielded crystals with the same unit cell and molecular structure, but a better data set could not be obtained. However, for comparison, we obtained bond lengths for **2** from the structure calculated at the r^2^SCAN‐3c/def2‐mTZVPP level of density functional theory (DFT). For **1**, the computed bond lengths match those obtained by scXRD within 0.03 Å, so we consider them trustworthy also for **2**. The P2═N2 bond in **2** (calc. 1.651 Å) is very similar to that in **1** (1.644(4) Å, calc. 1.654 Å), and it is thus best described as a double bond. Intriguingly, the NO bond in **2** (calc. 1.517 Å) is already very activated and resembles more an N−O single bond (avg. 1.394 Å) [37] rather than an N═O double bond (avg. 1.218 Å) [37]. The P1−O1 bond in **2** (calc. 1.601 Å) is considerably longer than in **1** (1.476(3) Å, calc: 1.504 Å) and in the range of P−O single bonds (avg. 1.590 Å) [37], thus leading to a reasonable description of the bridging motif as P═N−O−P^+^.

While the majority of the crystals in the targeted syntheses of **2** (Equation 2) are indeed the desired product, we always obtained few crystals of **1** as well, which could not be separated from the mixture and which cause signals of **1** to be visible in the NMR spectra of **2**. NMR spectra of the isolated crystals of **2** show a P‐P coupling constant of ^3^ *J*_P‐__P_ = 9.8 Hz over the NO bridge and higher order spin systems for the methylene and *tert*‐butyl groups, confirming the cyclic structure of **2**. In solution, **2** converts slowly to **1** over the course of multiple days. This slow transformation, however, stands in contrast to the very fast formation of **1** within minutes and implies that **2** is not necessarily an intermediate in the formation of **1**.

Note that the use of a good WCA such as [Al(OR^F^)_4_]^–^ is crucial for the observed reactivity. Reactions of nitrosonium salts with more strongly coordinating anions, for example, NO[BF_4_] and NO[SbF_6_], are much slower, need ultrasonication due to the low solubilities of these inorganic salts and yield, in addition to **1^+^** and **2^+^**, the signals of a much larger series of side products in the NMR spectra. Hence, only with the weakly coordinating and weakly interacting anion [Al(OR^F^)_4_]^–^ a fast NO^+^ scission at RT with **1** as main reaction product is achieved.

## 7 DFT Calculations of the Mechanism

**FIGURE 2:**
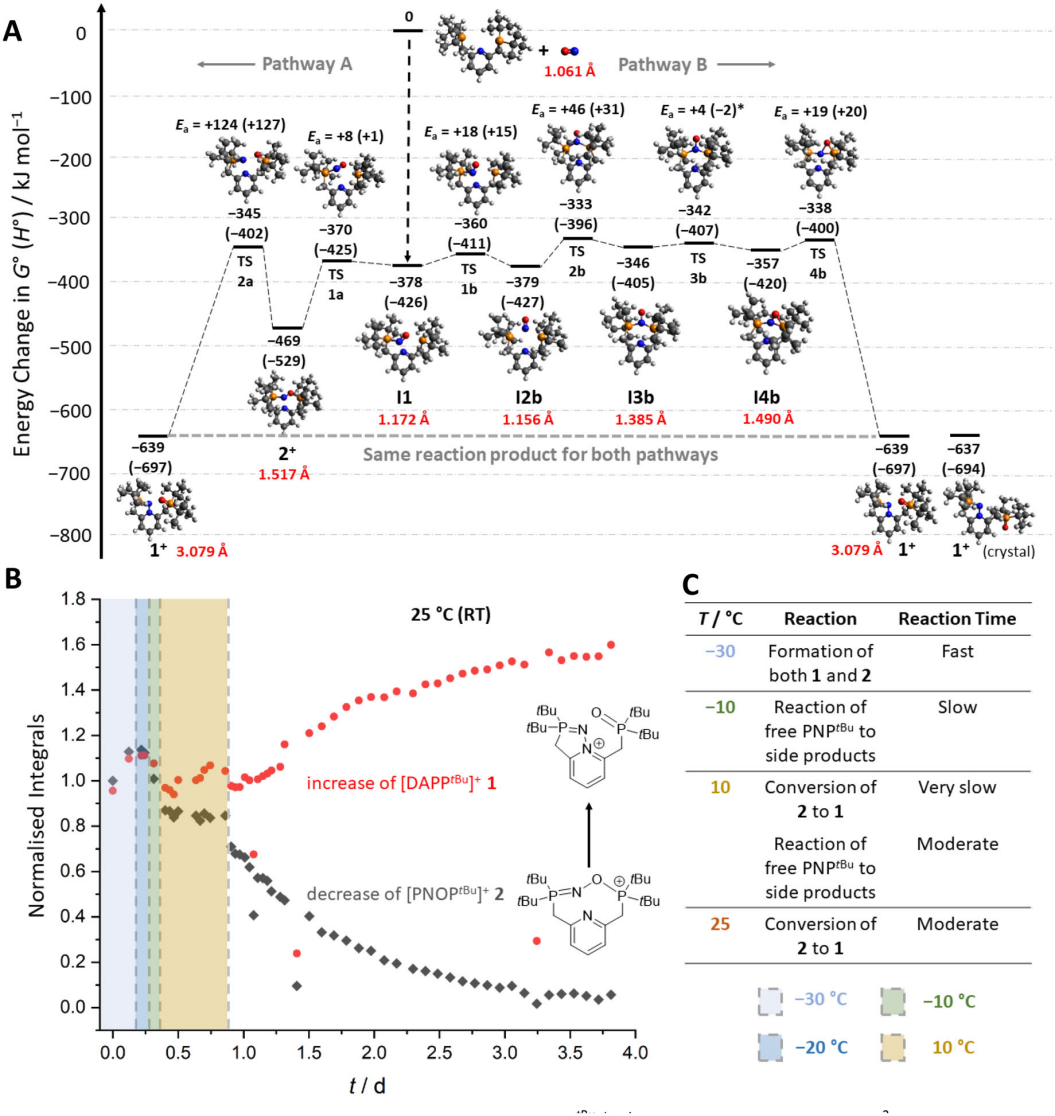
(A) Computed reaction profile for the formation of [DAPP^*t*Bu^]^+^ (**1** ^**+**^) in the gas phase at the RI‐r^2^SCAN‐3c(D4)/def2‐mTZVPP level of theory with computed N−O distances shown in red for all minimum structures. Intermediates in the mechanism are abbreviated as Inx, transition states as TSnx (*n* = running number, *x* = a or b indicating the two possible pathways). *The negative enthalpy of TS3b can be explained by its zero‐point energy and electronic energy close to 0 (see *E*_el_ plot in Figure S36). (B) Plot of the integrals of the low‐field signals in the ^31^P{^1^H} NMR spectra of [DAPP^*t*Bu^][Al(OR^F^)_4_] **1** (red) and [PNOP^*t*Bu^][Al(OR^F^)_4_] **2** (black) over time and at different temperatures. (C) Summary of observed reactions occurring in the reaction mixture at different temperatures with their reaction times.

To better understand the mechanism of the formation of **1**, we conducted quantum chemical calculations in the gas phase at the r^2^SCAN‐3c/def2‐mTZVPP level of DFT (Figure 2A). The overall reaction of NO^+^ and PNP*^t^* ^Bu^ to [DAPP*^t^* ^Bu^]^+^ (**1^+^**) is highly exergonic (∆*G* (∆*H*) = −639 (−697) kJ mol^−1^) showing an immense thermodynamic driving force. In particular, the first step of the reaction, the barrierless addition of NO^+^ to one phosphane group of PNP*^t^* ^Bu^ to form the first intermediate (**I1**), releases a large amount of energy (∆*G* (∆*H*) = −378 (−426) kJ mol^−1^). **I1** can then react exergonically to form [PNOP*^t^* ^Bu^]^+^ (**2^+^**) with a negligible barrier of ∆*G* ^‡^ = +8 kJ mol^−1^ (pathway A). Yet, the following conversion of **2^+^** to **1^+^** is kinetically hindered by a barrier of +124 kJ mol^−1^. Hence, once formed, **2^+^** is long‐living and NMR‐observable.

However, in reaction pathway B in Figure 2A, the formation of **1^+^** was investigated on an alternative route. Here, **I1** reacts to the symmetric intermediate **I2b**, which is almost isoenergetic with **I1** and in which the two phosphane groups are bridged via a P−N(═O)−P motif. From **I2b**, a flat potential energy surface (PES) with minimal barriers leads to **1^+^** over an ‘energy plateau’. Following this mechanism, the rate limiting step toward the formation of **1^+^** (∆*G* ^‡^ = +46 kJ mol^−1^) is easily surmountable at RT. This flat PES also explains the multitude of side products, as—apparently—leaving this plateau to follow various other ‘valleys’ may lead to many independent local minima on the PES.

Note that in neither pathway neutral NO gas is released and that all computed species are diamagnetic. This mechanism contrasts the previous report on the reaction of NO^+^ with a redox‐active FLP, where in fact neutral NO gas is the species that is activated by the FLP radical cation, and deelectronation and bond activation take place at different sites in the molecule [47]. By contrast, our system described in Figure 2A represents a direct unimolecular activation of the N≡O^+^ triple bond, that is, the intramolecular bond activation takes place as the consequence of adduct formation and does not require a preceding bimolecular electron transfer step in the periphery of the of molecule.

To experimentally test whether an initial deelectronation of the PNP*^t^* ^Bu^ ligand by [9,10‐dichlorooctafluoroanthracene][Al(OR^F^)_4_] (with a potential of 1.42 V vs. Fc^+^/Fc) [27] in the presence of an NO gas atmosphere would lead to **1** or **2**, we reacted these components in pentafluorobenzene solution. Yet very broad signals in the ^31^P NMR spectrum of this reaction (which are not in the spectral range of radical species) suggest these correspond to oligomers of PNP*^t^* ^Bu^, resulting from a reaction of [PNP*^t^* ^Bu^]^+^ with itself. No signals corresponding to **1** or **2** were observed. Presumably, the reaction of [PNP*^t^* ^Bu^]^+^ with itself is much faster than the reaction with NO under these conditions. Thus, a mechanism related to the recently published NO scission in Ref [47], appears unlikely in our case and indicates direct scission of NO^+^.

## 8 Refined VT‐NMR Measurements

To further validate these computed mechanisms for the formation of **1** and **2**, we performed NMR measurements at variable temperatures (VT, Figure 2B,C). For this purpose, the starting materials were dissolved in CD_2_Cl_2_ at −30°C, then introduced into the pre‐cooled NMR spectrometer, and measured at −30°C. The initial spectrum already shows **1** and **2** in a 1:1 ratio, alongside unreacted PNP*^t^* ^Bu^ (arising from the low solubility of NO[Al(OR^F^)_4_] in CD_2_Cl_2_ at −30°C). After 3 h at −30°C, the formation of **1** and **2** from the reactants present in solution was complete. The mixture was slowly warmed to 10°C, while measuring spectra at −20 and −10°C. Only at 10°C, the very slow conversion of **2** into **1** started, while the remaining PNP*^t^* ^Bu^ ligand gave rise to various side products. We suspect that all the NO[Al(OR^F^)_4_] only fully dissolved at temperatures above −10°C, where side reactions are highly favored.

Subsequently, room temperature spectra were measured for further 3 days to investigate the kinetics of the formation of **1** and to elucidate the reaction order of the conversion. In Figure 2B, the concentrations of **1** and **2** are plotted against the time, and in Figures S31 and S32, their logarithms are plotted. The logarithmic plots confirm first‐order kinetics for the formation of **1** out of **2**, which are consistent with a unimolecular mechanism as suggested by the quantum chemical calculations. The rate constant for the decay of **2** was determined as *k*_decrease_ = −(6.74 ± 0.14) · 10^−4^ min^−1^ and the rate constant of the increase of **1** amounts to *k*_increase_ = (1.53 ± 0.04) · 10^−4^ min^−1^.^2^

Interestingly, when the reactants are dissolved in CD_2_Cl_2_ at RT and directly measured by NMR spectroscopy, not only **1** is present in the product mixture but also a smaller amount of **2** (ratio 3:1). The formation of both **1** and **2** at RT is not necessarily comprehensible from the computed mechanism alone as the chemical equilibrium should clearly favor the formation of **1** (ΔΔ*G* = −170 kJ mol^−1^, which corresponds to *K* = e^–ΔΔ^ *^G^* ^/(RT)^ = 6 ∙ 10^29^) and should be easily reached at RT due to the overall low barriers toward both the formations of **1** and **2**. This apparent contradiction most likely arises from the competition between energy dissipation into the medium (after formation of **I1**) and formation of **1**. The highly exergonic formation of **I1** (∆*G* = −378 kJ mol^−1^) creates a high local energy surplus, first stored in the adduct **I1**, that can on the one hand lead to the productive formation of **1** via pathway B (and to a considerably lesser amount to the formation of **2**). On the other hand, the energy can dissipate (e.g., via solvent interaction) favoring the formation of **2** via a smaller barrier. At lower temperatures, energy dissipation dominates, explaining the higher ratio of formed **2** via pathway A. At −30°C, formed **2** is then trapped in an ‘energetic sink’ and can only react at higher temperatures to **1** either via TS2a (∆*G* ^‡^ = +124 kJ mol^−1^) or by first reacting back to **I1** and traversing the energetic plateau of pathway B (∆*G* ^‡^_total_ = +136 kJ mol^−1^).

## 9 Conclusion

We report a direct and metal‐free cleavage of the nitrosonium triple bond using NO[Al(OR^F^)_4_] and the diphosphane PNP*^t^* ^Bu^. In this reaction, the structurally unprecedented 1,2,3‐diazaphospholo[1,5‐*a*]pyridinium derivative [DAPP*^t^* ^Bu^]^+^[Al(OR^F^)_4_]^–^ **1** is formed. As shown computationally and experimentally, the formation of **1** can proceed either via the isolable intermediate [PNOP*^t^* ^Bu^]^+^[Al(OR^F^)_4_]^–^ **2** or via various intermediates on a low‐barrier pathway. In our studies, we focused on an intramolecular mechanism, which is strongly supported by the temperature dependent kinetic NMR studies revealing a first order mechanism of the interconversion of **2** to **1**. The use of a good weakly coordinating anion is crucial for the observed reactivity as the use of the smaller and more strongly interacting anions [BF_4_]^−^ and [SbF_6_]^−^ leads to longer reaction times and a wider range of side products.

## Conflicts of Interest

The authors declare no conflicts of interest.

## Supporting information




**Supporting File 1**: The electronic Supporting Information contains the general synthetic methods and characterization techniques used for this work together with the experimental procedures. Additional figures such as NMR, IR, and Raman spectra are presented, as well as the crystallographic data of the isolated salts and details of the quantum chemical calculations and kinetic studies. The authors have cited additional references within the Supporting Information [48–71].

**Supporting File 2**: anie71442‐sup‐0002‐Data.zip.

## Data Availability

X‐ray crystallographic data are available free of charge from the Cambridge Crystallographic Data Centre (CCDC) under the reference numbers 2497214 ([DAPP^*t*Bu^][Al(OR^F^)_4_] 1) and 2497215 ([PNOP^*t*Bu^][Al(OR^F^)_4_] 2) via https://www.ccdc.cam.ac.uk/structures/. All other data are contained in the main text or in the Supporting Information.

## Endnotes

1 Reports of bond dissociation energies (BDEs) of small molecules differ slightly in the literature, but the differences are in general small, ranging from 3 to 7 kJ mol^−1^.

2 Note that both rate constants are in the same order of magnitude and that the discrepancy most probably stems from errors in the integration of the signals.
