# Supplementary material for: Metal‐Free Scission of the NO+ Triple Bond
Source: Angew Chem Int Ed Engl. 2026 Feb 15;65(13):e24527. doi: 10.1002/anie.202524527 (PMC13007579; doi:10.1002/anie.202524527)
Supplement: Supplementary file 1 — Supporting File 1: The electronic Supporting Information contains the general synthetic methods and characterization techniques used for this work together with the experimental procedures. Additional figures such as NMR, IR, and Raman spectra are presented, as well as the crystallographic data of the isolated salts and details of the quantum chemical calculations and kinetic studies. The authors have cited additional references within the Supporting Information [48–71]. [file ANIE-65-e24527-s002.pdf]

Supporting Information

Room Temperature Metal-Free Cleavage of the NO^+ Triple Bond

Julie Willrett[a], Harald Scherer[a], Burkhard Butschke[a] and Ingo Krossing*[a]

[a] M. Sc. J. Willrett, Dr. Harald Scherer, Dr. Burkhard Butschke, Prof. Dr. I. Krossing, Institut für Anorganische und Analytische Chemie and Freiburger Materialforschungszentrum (FMF), Albert-Ludwigs-Universität Freiburg, Albertstr. 21, 79104 Freiburg, Germany.

* E-Mail: krossing@uni-freiburg.de.

Table of contents

1. Abbreviations 2
2. Material and Methods 3
3. Detailed Syntheses with NMR and Vibrational Spectra 5
3.1 PNP^{tBu} 5
3.2 [DAPP^{tBu}][Al(OR^F)$_4$] **1** 6
3.3 [PNOP^{tBu}][Al(OR^F)$_4$] **2** 12
4. NMR Spectroscopy and Kinetic Analysis 21
5. Single-Crystal XRD Data 29
6. Computational details 47
7. References 69

1. Abbreviations

[Al(OR^F)]$^-$ = [Al{OC(CF$_3$)$_3$}$_4$]$^-$

[DAPP^{tBu}]$^+$ = 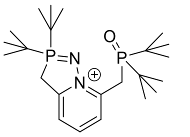

DFT = density functional theory

FTIR = Fourier-transform infrared spectroscopy

FWHM = full width at half maximum

HMBC = heteronuclear multiple bond correlation

HSQC = heteronuclear single quantum coherence

OR^F = OC(CF$_3$)$_3$

[PNOP^{tBu}]$^+$ = 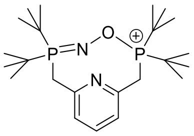

PNP^{tBu} = 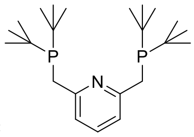

RT = room temperature

TS = transition state

XRD = X-ray diffraction

2. Material and Methods

General procedure

All manipulations were carried out under an argon atmosphere and exclusion of air and moisture using a MBraun glovebox filled with argon (O_2/H_2O < 1 ppm) and standard Schlenk techniques. All reactions were carried out in Schlenk tubes with grease-free PTFE valves. All glassware was dried over-night in an oven at 150 °C and was thoroughly flame-dried prior to usage. The solvents DCM (Sigma Aldrich) and CD_2Cl_2 (Deutero) were pre-dried over CaH_2 and condensed onto activated 3 Å molecular sieves. 1,2,3,4-Fluorobenzene (4FB, Fluorochem) was pre-dried over CaH_2. 4FB is usually contaminated with traces of less fluorinated benzenes. To remove these impurities, 4FB was stirred over $Ag[Al(OR^F)_4]$ at room temperature and distilled onto activated 3 Å molecular sieves. Oxygen was removed from all solvents by three freeze-pump-thaw cycles. $NO[Al(OR^F)_4]$[1] and PNP^{tBu}[2] were prepared using literature known syntheses.

Vibrational Spectroscopy

ATR FTIR spectra were recorded inside a glovebox with a Bruker ALPHA spectrometer equipped with a QuickSnap Platinum ATR sampling module and a ZnSe crystal. Spectra were measured at RT with 32 scans and a resolution of 2 cm^{-1} in the range of 4000-550 cm^{-1}.
Raman measurements were carried out at on a Vertex 70 IR spectrometer with installed RAM II Raman module (1064 nm exciting line of a Nd-YAG laser) and liquid nitrogen cooled Ge detector. Samples were flame-sealed in glass Pasteur pipettes. Spectra were recorded at RT in a range from 4000-80 cm^{-1} and a resolution of 4 cm^{-1} with a laser power of 50 mW and 1,000 scans.
Spectroscopic data were processed with the Bruker OPUS 7.5 software and baseline corrections were carried out with five iterations. The signal intensity was normalized and the relative IR intensities were reported as followed with regard to the most intensive signal: ≥ 0.7 = very strong (vs), ≥ 0.6 strong (s), ≥ 0.5 = medium strong (ms), ≥ 0.4 = medium (m), ≥ 0.3 = medium weak (mw), ≥ 0.2 = weak (w), < 0.2 = very weak (vw). Raman intensities are not determined and reported due to considerable fluorescence.

NMR Spectroscopy

NMR spectra were recorded at RT on a Bruker DPX 200 MHz, a Bruker Avance III HD 300 MHz or a Bruker AvanceIVNeo WB 400 MHz spectrometer. The samples were dissolved in CD_2Cl_2 (0.6 mL) in a 5 mm NMR tube with J. Young PTFE valve under inert conditions (argon atmosphere). The spectra were calibrated by using the residual ^{1}H signal of the solvent $CDHCl_2$ (δ = 5.32 ppm, rel. to TMS).[3] The calibration of the spectra of other nuclei were adjusted accordingly using die IUPAC Ξ-table.[4] Spectroscopic data was analysed and plotted using Bruker TopSpin 4.1.3 software. ^{1}H NMR spectra are referenced against TMS, ^{19}F NMR spectra against $CFCl_3$, ^{27}Al NMR spectra against an aqueous solution of $Al(NO_3)_3$ (1.1 mM) and ^{31}P NMR spectra against 85 % H_3PO_4 in H_2O. The broad signal at around 60 ppm in the ^{27}Al spectra stems from the probe head.

Single Crystal X-Ray Diffraction

Single crystal X-ray diffraction data was acquired using a D8 Venture Photon III HPAD (Bruker) detector diffractometer. Crystal selection was performed at RT under PFPE oil JC 1800 (Sunoit Performance Material Science). Crystals were mounted on 0.1 to 0.2 mm diameter CryoLoops and were shock-cooled using an Oxford Cryostream 800 low temperature device.[5] Data were collected at 100(2) K using monochromatic MoK_α radiation (λ = 0.71073 Å). Crystallographic data were integrated with SAINT (version 8.40B) and a multi-scan absorption correction using SADABS or TWINABS was

performed.[6] Structures were solved by direct methods with SHELXT[7] and refined by full-matrix least-squares methods against F^2 by SHELXL-2019/3[8] using the GUI software ShelXle.[9] Refinement of disordered moieties was done using bond lengths restraints and displacement parameter restraints and was performed using the program DSR.[10] Finalization of the gathered data was done with FinalCif.[11] Graphical representations of the crystal structures were created using Mercury (version 2022.3.0). Crystallographic data for the structures reported here have been deposited with the Cambridge Crystallographic Data Centre (CCDC numbers: 2497214 (**1**), 2497215 (**2**)).[12]

Computational Details

All quantum chemical calculations were performed with the ORCA programme package (version 5.0).[13] Density functional theory (DFT) with the r^{2}SCAN-3c functional[14] and def2-mTZVPP basis set[15] was used with the resolution-of-identity (RI) approximation[16] and dispersion correction (D4).[17] All computed minimum structures were checked for the absence of imaginary vibrational frequencies and computed transition state structures were checked for the presence of a single imaginary vibrational frequency. Transition state structures were determined by performing relaxed surface scans or nudged elastic band (NEB)[18] calculations. In order to link a transition state to the corresponding minimum structures, an IRC[19] calculation was performed with ORCA. The resulting structures were either directly identified as minimum structures or subjected to a further geometry optimization.
IR spectra were simulated without scaling factor and a FWHM of 30 cm^{-1} at the RI-r^{2}SCAN-3c(D4)/def2-mTZVPP level of theory. Raman activities were simulated at the RI-r^{2}SCAN-3c(D4)/def2-mTZVPP level of theory and plotted with a FWHM of 30 cm^{-1}.

3. Detailed Syntheses with NMR and Vibrational Spectra

3.1 PNP^{tBu}

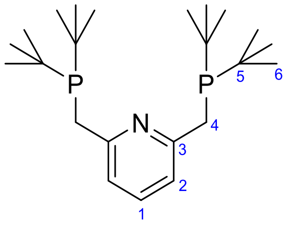

PNP^{tBu} was prepared following and slightly modifying a literature known synthesis.[2]

n-BuLi (2.5 M in hexane, 7.50 mL, 18.6 mmol, 2.0 equiv.) was added to a solution of 2,6-lutidine (1.08 mL, 9.33 mmol) in Et_2O (15 mL) at 0 °C over the course of 30 min leading to a colour change of the solution to orange. The reaction mixture was stirred overnight at 40 °C leading to a colour change to dark red and the formation of a solid. The mixture was cooled to −78 °C and $(tBu)_2PCl$ (3.60 mL, 18.6 mmol, 2.0 equiv.) was added slowly. The reaction mixture was brought to room temperature and stirred for 30 min, then degassed water (10 mL) was added to quench the reaction. The organic phase was decanted and dried with Na_2SO_4, filtered off and allowed to crystallize at −30 °C to obtain PNP^{tBu} as a slightly yellow crystalline solid (1.16 g, 2.93 mmol, 31 %). This crude product was recrystallized from Et_2O three times to obtain the product in a higher purity as colourless crystals. NMR spectroscopic data stems from the purified crystals.

^{1}H NMR (300.18 MHz, CD_2Cl_2, RT): δ = 7.46 (1 H, 1-H, t, $^{3}J_{H\text{-}H}$ = 7.7 Hz), 7.18 (2 H, 2-H, d, $^{3}J_{H\text{-}H}$ = 7.7 Hz), 2.98 (4 H, 4-CH_2, d, $^{2}J_{H\text{-}P}$ = 3.2 Hz), 1.13 (36 H, 6-CH_3, d, $^{3}J_{H\text{-}P}$ = 10.8 Hz) ppm.

^{13}C NMR (75.48 MHz, CD_2Cl_2, RT): δ = 161.0 (2 C, 3-C), 135.6 (1 C, 1-C), 120.3 (2 C, 2-C), 31.6 (2 C, 4-C), 31.6 (4 C, 5-C), 29.4 (12 C, 6-C) ppm. *^{13}C NMR shifts taken from ^{1}H,^{13}C-HMBC spectrum.*

^{31}P{^{1}H} NMR (81.01 MHz, CD_2Cl_2, RT): δ = 35.6 (2 P) ppm.

3.2 [DAPP^{tBu}][Al(OR^F)$_4$] **1**

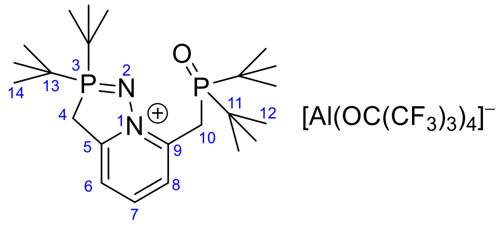

NO[Al(OR^F)$_4$] (40.0 mg, 40.1 µmol) and PNP^{tBu} (15.9 mg, 40.1 µmol, 1.0 equiv.) were dissolved in DCM at RT and stirred overnight. The orange solution was layered with *n*-heptane (7 mL) to obtain colourless crystals of **1** (8.6 mg, 6.2 µmol, 15 %) in a dark oil. Vibrational and NMR spectroscopic data stems from the isolated crystals.

^{1}H NMR (400.17 MHz, CD_2Cl_2, RT): δ = 8.03 (1 H, 8-H, ABC signal, higher order), 7.51 (1 H, 6-H, ABC signal, higher order), 7.49 (1 H, 7-H, ABC signal, higher order), 3.85 (2 H, 4-CH_2, d, $^2J_{H\text{-}P}$ = 8.8 Hz), 3.81 (2 H, 10-CH_2, d, $^2J_{H\text{-}P}$ = 9.9 Hz), 1.41 (18 H, 14-CH_3, d, $^3J_{H\text{-}P}$ = 16.3 Hz), 1.29 (18 H, 12-CH_3, d, $^3J_{H\text{-}P}$ = 14.1 Hz) ppm. *Solvent signal ($CDHCl_2$) visible at 5.32 ppm and grease signal visible at 0.09 ppm.*

^{13}C NMR (100.62 MHz, CD_2Cl_2, 243 K): δ = 144.0 (1 C, 5-C), 143.3 (1 C, 9-C), 129.8 (1 C, 7-C), 129.3 (1 C, 8-C), 122.3 (1 C, 6-C), 37.3 (2 C, 13-C), 36.6 (2 C, 11-C), 26.4 (6 C, 12-C), 25.5 (6 C, 14-C), 23.3 (1 C, 10-C), 23.0 (1 C, 4-C) ppm. *^{13}C NMR shifts taken from ^{1}H,^{13}C-HSQC and ^{1}H,^{13}C-HMBC spectra measured at 243 K.*

^{19}F NMR (376.53 MHz, CD_2Cl_2, RT): δ = –75.7 (36 F, [Al(OR^F)$_4$]$^-$, s) ppm.

^{27}Al NMR (104.27 MHz, CD_2Cl_2, RT): δ = 34.6 (1 Al, [Al(OR^F)$_4$]$^-$, s) ppm.

^{31}P{^{1}H} NMR (161.99 MHz, CD_2Cl_2, RT): δ = 82.7 (1 P, N=P), 61.2 (1 P, O=P) ppm.

FTIR (ZnSe, ATR): $\tilde{\nu}$/cm^{-1} = 2973 (vw), 2880 (vw), 1594 (vw), 1575 (vw), 1475 (vw), 1455 (vw), 1398 (vw), 1351 (vw), 1296 (m), 1274 (s), 1240 (s), 1211 (vs), 1166 (m), 1021 (vw), 969 (vs), 936 (w), 831 (w), 755 (vw), 726 (vs), 651 (vw), 630 (vw), 560 (w).

FT Raman (1,000 scans, 50 mW): $\tilde{\nu}$/cm^{-1} = 2939, 1271, 798, 569.

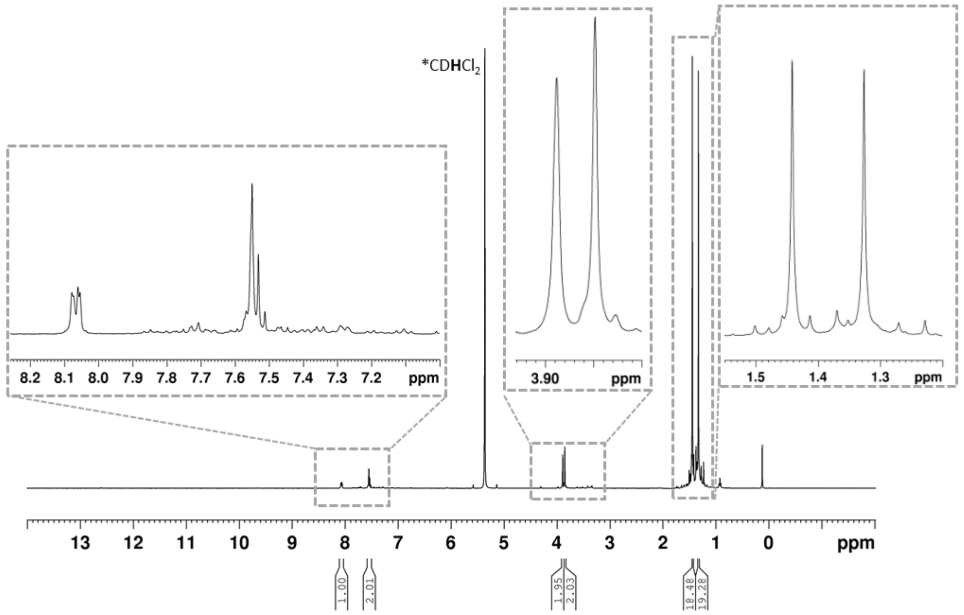

Figure S 1: ^{1}H NMR spectrum (400.17 MHz, CD_2Cl_2, RT) of [DAPP^{tBu}][$Al(OR^F)_4$] **1**.

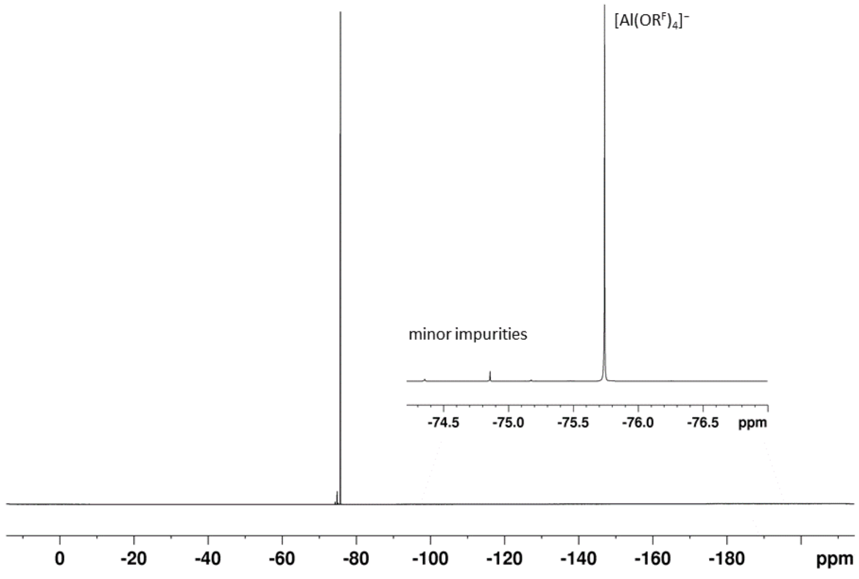

Figure S 2: ^{19}F NMR spectrum (376.53 MHz, CD_2Cl_2, RT) of [DAPP^{tBu}][$Al(OR^F)_4$] **1**.

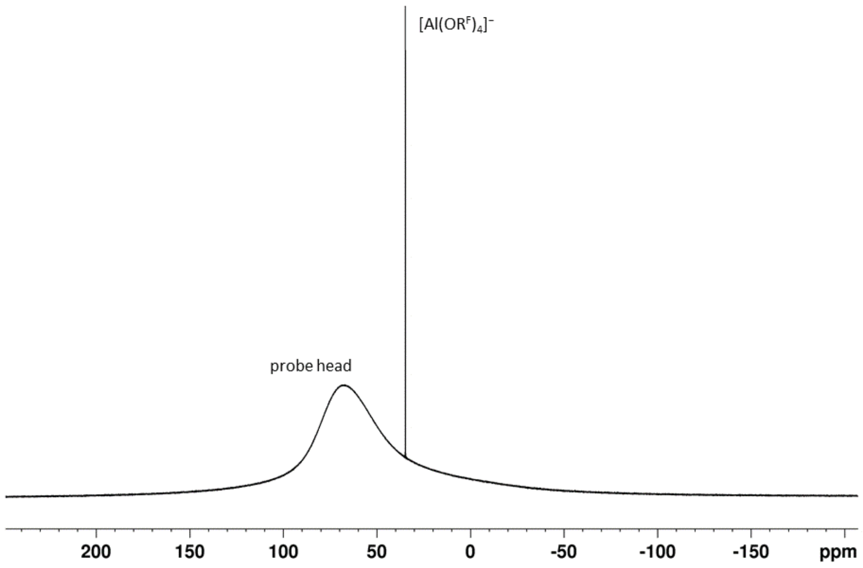

Figure S 3: ^{27}Al NMR spectrum (104.27 MHz, CD_2Cl_2, RT) of [DAPP^{tBu}][Al(OR^F)$_4$] **1**.

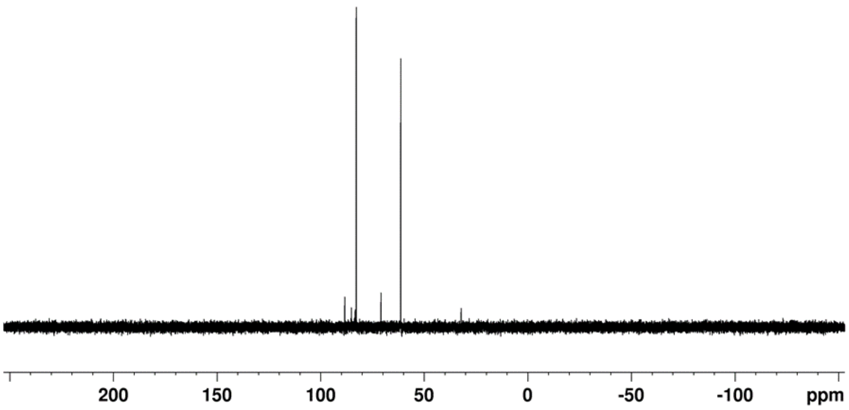

Figure S 4: ^{31}P{^{1}H} NMR spectrum (161.99 MHz, CD_2Cl_2, RT) of [DAPP^{tBu}][Al(OR^F)$_4$] **1**.

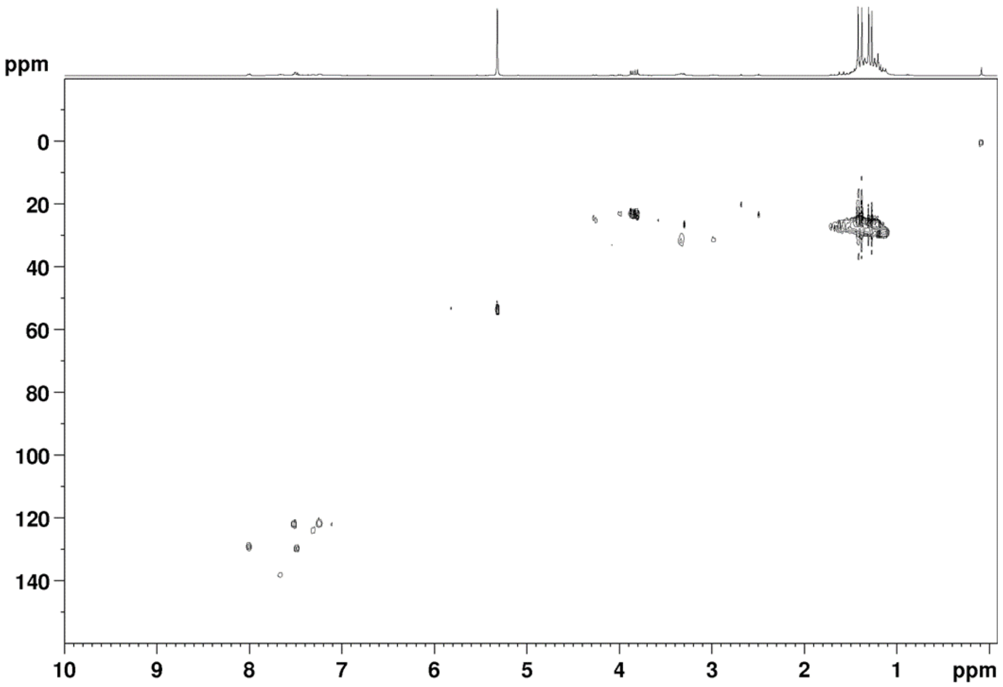

Figure S 5: $^{1}H,^{13}C$-HSQC NMR spectrum (400.17 MHz and 100.62 MHz, CD_2Cl_2, 243 K, optimised for J = 145 Hz) of [DAPP^{tBu}][Al(OR^F)$_4$] **1**.

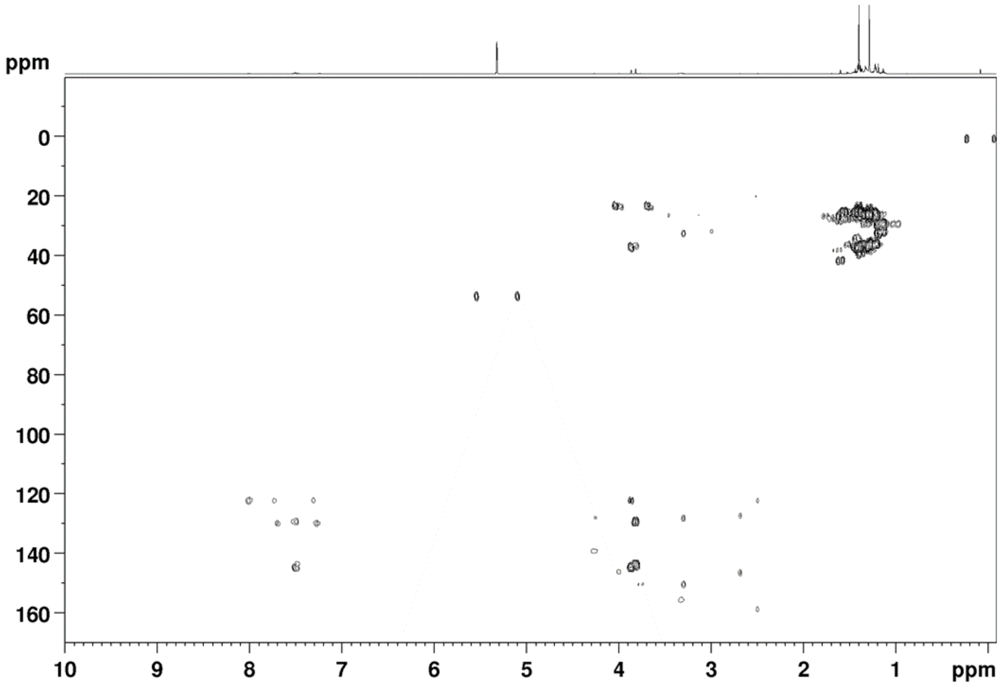

Figure S 6: $^{1}H,^{13}C$-HMBC NMR spectrum (400.17 MHz and 100.62 MHz, CD_2Cl_2, 243 K, optimised for J = 8 Hz) of [DAPP^{tBu}][Al(OR^F)$_4$] **1**.

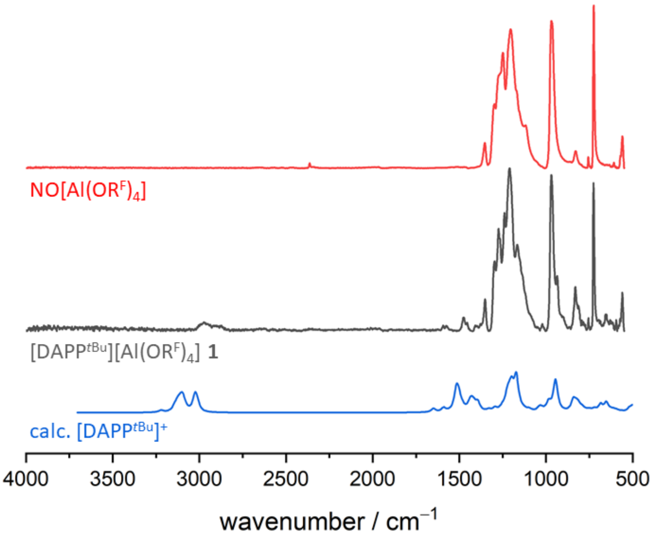

Figure S 7: IR spectra (32 scans, ZnSe) of NO[Al(OR^F)$_4$] (red) and of [DAPP^{tBu}][Al(OR^F)$_4$] **1** (black) as well as the calculated spectrum of [DAPP^{tBu}]$^+$ at the r2SCAN-3c/def2-mTZVPP level of theory (blue).

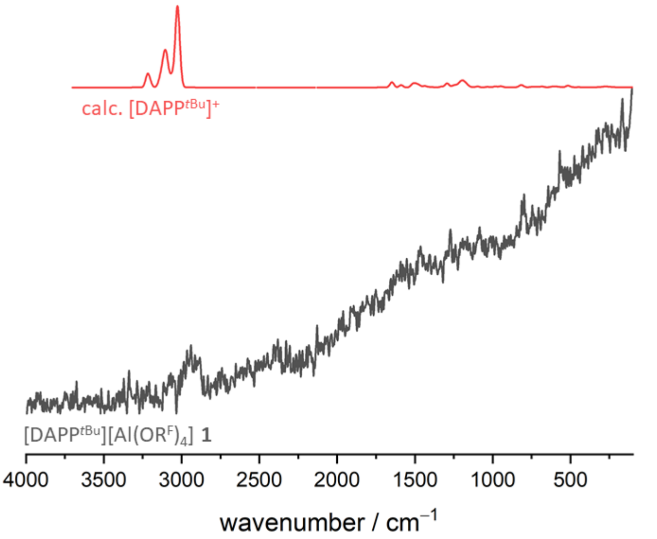

Figure S 8: Raman spectrum (1,000 scans, 50 mW) of [DAPP^{tBu}][Al(OR^F)$_4$] **1** (black) and calculated spectrum of [DAPP^{tBu}]$^+$ at the r2SCAN-3c/def2-mTZVPP level of theory (red).

Table S 1: Assignment of IR and Raman vibrations of [DAPP^{tBu}][Al(OR^F)$_4$] **1** and comparison with the calculated spectra for cation bands and with the reference compound [NEt$_4$][Al(OR^F)$_4$] for anion bands.[20] Due to the uneven baseline in the experimental Raman spectrum (caused by fluorescence), no Raman intensities are reported.

**1**				**[Al(OR^F)$_4$]$^-$**		**Assignment[a]**
**exp. IR**	**calc. IR**	**exp. Raman**	**calc. Raman**	**IR**	**Raman**	
560 (w)		569		562 (mw)	563 (w)	Anion, Al−O, C−C
630 (vw)	649					δ P−N−N, C−N−C, C−P−O
651 (vw)	685					δ P−N−N, C−N−C
726 (vs)				727 (s)		Anion, C−C, C−O
755 (vw)				756 (mw)		Anion, Al−O
		798			798 (s)	–
831 (w)				833 (m)		Anion, Al−O, C−C
936 (w)	944					ν P−N, C−C
969 (vs)				973 (s)		Anion, C−C, C−F
1021 (vw)	1035					δ C−C−H
1166 (m)				1176 (ms)		Anion, C−C, C−F
			1197[b]			δ H−C−C, ν P−P
1211 (vs)				1217 (vs)		Anion, C−C, C−F
1240 (s)				1240 (s)		Anion, C−C, C−F
1274 (s)		1271		1274 (vs)	1274 (mw)	Anion, C−C, C−F
			1293[b]			δ H−C−P
1296 (m)				1298 (s)		Anion, C−C, C−F
1351 (vw)				1353 (ms)		Anion, C−C, C−F
1398 (vw)	1392					δ H−C−H
1455 (vw)	1433					δ H−C−H
1475 (vw)	1508		1496[b]			δ H−C−H
1575 (vw)	1590					arene in plane C−C, C−H, C−N
1594 (vw)	1649		1647[b]			arene in plane C−C, C−H
2880 (vw)	3022					ν C−H
		2939 (br.)	3029			ν C−H
2973 (vw)	3097					ν C−H
			3105[b]			ν C−H
			3212[b]			ν arene C−H

[a] From a visualization of the calculated spectra.
[b] Not observed in the experimental spectrum due to their low intensity and high noise.

3.3 [PNOP^{tBu}][Al(OR^F)$_4$] 2

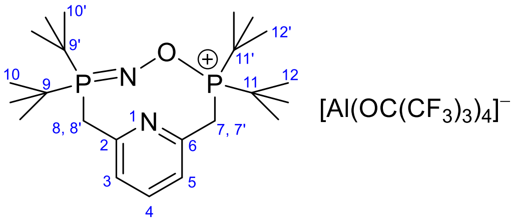

NO[Al(OR^F)$_4$] (50.0 mg, 50.1 µmol) and PNP^{tBu} (19.8 mg, 50.1 µmol, 1.0 equiv.) were suspended in DCM at −30 °C and stirred for 30 min. The yellow solution was layered with *n*-heptane (7 mL) and stored at −30 °C to obtain colourless crystals of **2** in a dark oil. Vibrational and NMR spectroscopic data stems from the isolated crystals. The crystals consist of an inseparable mixture of **2** and **1**.

^{1}H NMR (400.17 MHz, CD_2Cl_2, 243 K): δ = 7.63 (1 H, 4-H, ABC signal, higher order), 7.17 (1 H, 5-H, ABC signal, higher order), 7.15 (1 H, 3-H, ABC signal, higher order), 3.96 (1 H, 8-H, higher order), 3.75 (1 H, 7-H, higher order), 3.45 (1 H, 8’-H, higher order), 3.43 (1 H, 7’-H, higher order), 1.40 (27 H, 10-H, 10’-H, 12-H, higher order), 1.15 (9 H, 12’-H, higher order) ppm. *Solvent signal ($CDHCl_2$) visible at 5.32 ppm. Coupling constants could not be taken from higher order signals due to superposition of signals.*

^{13}C NMR (100.62 MHz, CD_2Cl_2, 243 K): δ = 150.2 (at RT, 1 C, 6-C), 149.8 (at RT, 1 C, 2-C), 138.5 (1 C, 4-C), 121.2 (1 C, 5-C), 120.3 (1 C, 3-C), 37.0 (at RT, 3 C, 9-C, 9’-C, 11-C), 36.3 (at RT, 1 C, 11’-C), 32.9 (1 C, 7-C), 30.6 (1 C, 8-C), 27.2 (9 C, 10-C, 10’-C, 12-C), 26.5 (3 C, 12’-C) ppm. *^{13}C NMR shifts taken from the ^{1}H,^{13}C-HSQC spectrum measured at 243 K, shifts of quaternary C nuclei taken from the ^{1}H,^{13}C-HMBC spectrum measured at RT.*

^{19}F NMR (376.53 MHz, CD_2Cl_2, 243 K): δ = –75.8 (36 F, [Al(OR^F)$_4$]$^-$, s) ppm.

^{27}Al NMR (104.27 MHz, CD_2Cl_2, 243 K): δ = 34.6 (1 Al, [Al(OR^F)$_4$]$^-$, s) ppm.

^{31}P{^{1}H} NMR (161.99 MHz, CD_2Cl_2, 243 K): δ = 97.6 (1 P, N=P, $^3J_{P-P}$ = 9.8 Hz), 58.5 (1 P, O=P, $^3J_{P-P}$ = 9.8 Hz) ppm.

FTIR (ZnSe, ATR): $\tilde{\nu}$/cm^{-1} = 2958 (vw), 2903 (vw), 1576 (vw), 1475 (vw), 1455 (vw), 1410 (vw), 1351 (vw), 1297 (m), 1274 (s), 1239 (vs), 1212 (vs), 1150 (s), 1020 (vw), 972 (vs), 935 (w), 832 (w), 813 (vw), 772 (vw), 755 (vw), 745 (vw), 727 (vs), 693 (vw), 654 (vw), 620 (vw), 593 (vw), 571 (vw), 559 (vw).

FT Raman (5,000 scans, 100 mW): $\tilde{\nu}$/cm^{-1} = 3085, 2979, 2924, 1618, 1466, 1299, 1275, 1152, 810, 798, 745, 572, 521, 358, 322, 280.

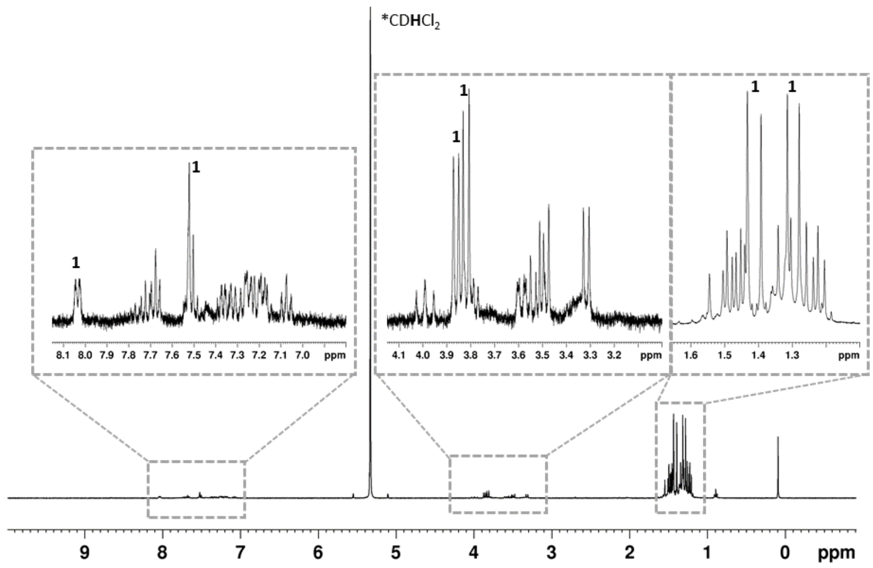

Figure S 9: ^{1}H NMR spectrum (400.17 MHz, CD_2Cl_2, 243 K) of [PNOP^{tBu}][Al(OR^{F})$_4$] **2**. Crystals of **2** could not be separated from crystals of **1**.

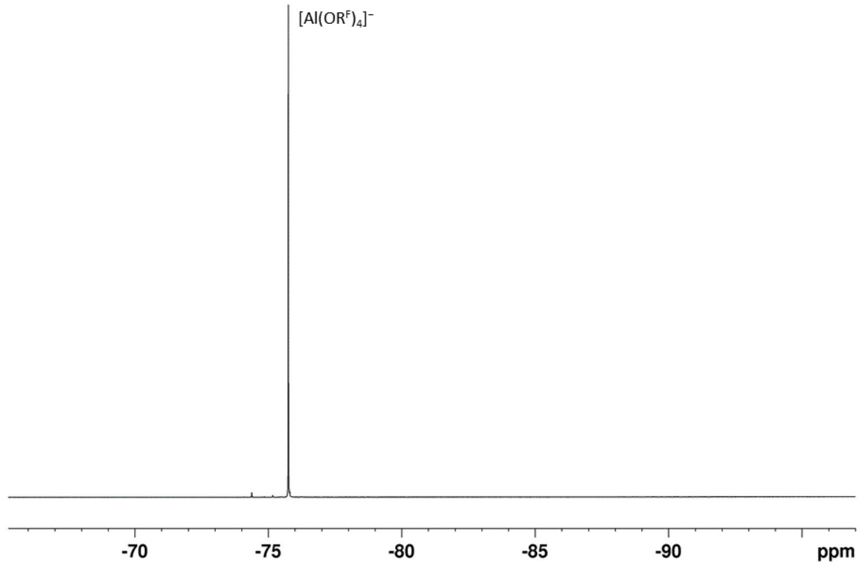

Figure S 10: ^{19}F NMR spectrum (376.53 MHz, CD_2Cl_2, 243 K) of [PNOP^{tBu}][Al(OR^{F})$_4$] **2**. Crystals of **2** could not be separated from crystals of **1**.

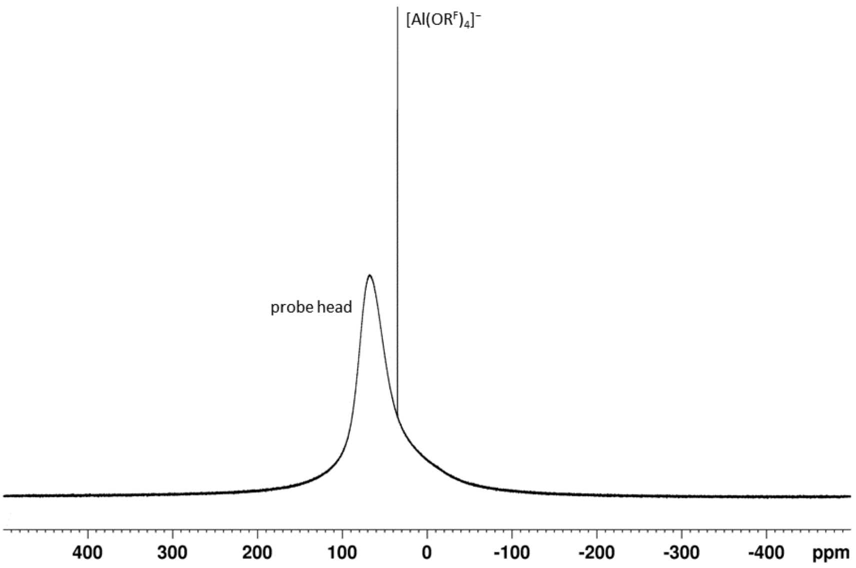

Figure S 11: ^{27}Al NMR spectrum (104.27 MHz, CD_2Cl_2, 243 K) of $[PNOP^{tBu}][Al(OR^F)_4]$ **2**. Crystals of **2** could not be separated from crystals of **1**.

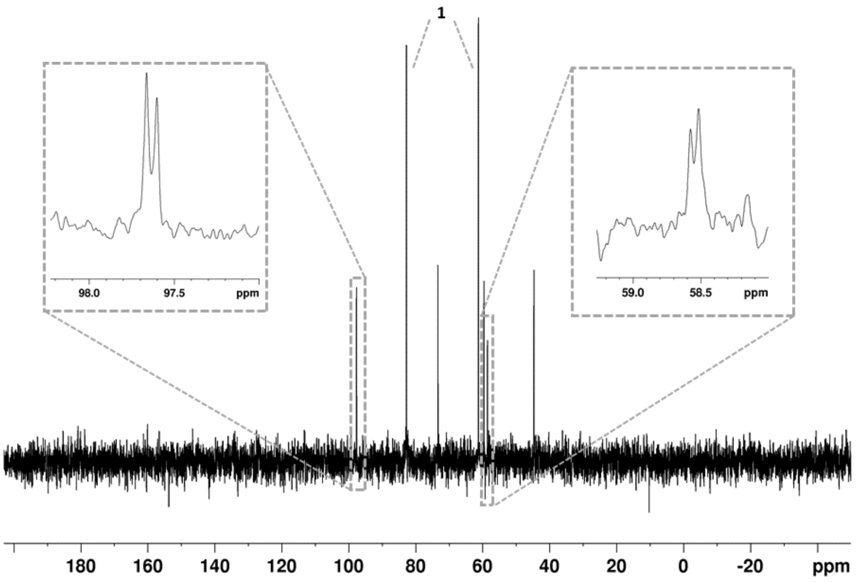

Figure S 12: $^{31}P\{^1H\}$ NMR spectrum (161.99 MHz, CD_2Cl_2, 243 K) of $[PNOP^{tBu}][Al(OR^F)_4]$ **2**. Crystals of **2** could not be separated from crystals of **1**.

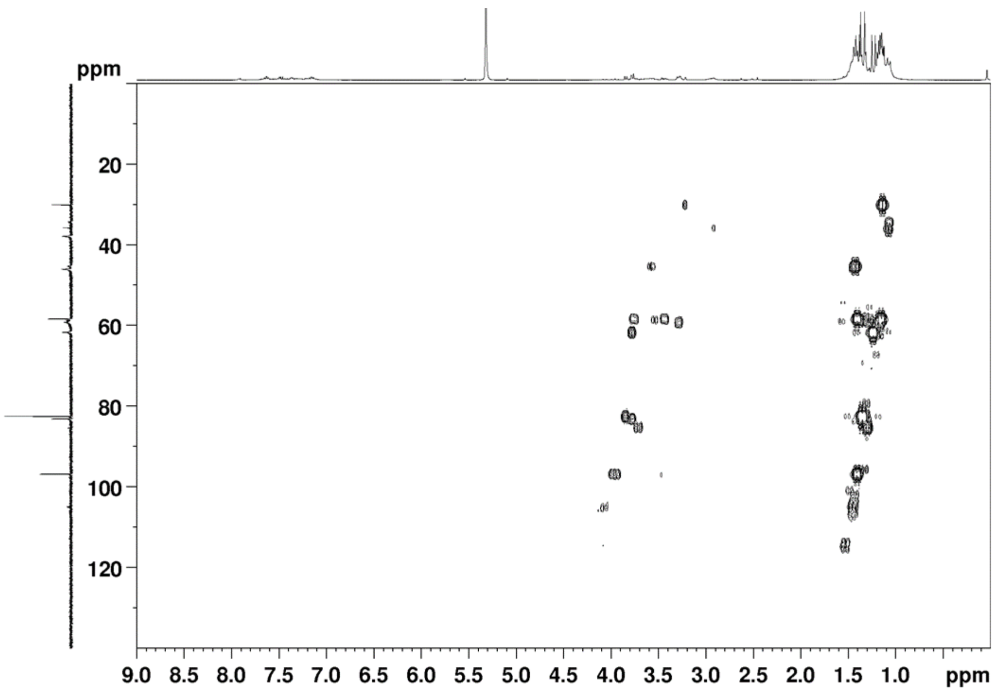

Figure S 13: $^{1}H,^{31}P$-HMBC NMR spectrum (400.17 MHz and 161.99 MHz, CD_2Cl_2, 243 K, optimised for J = 15 Hz) of a reaction mixture containing **2**.

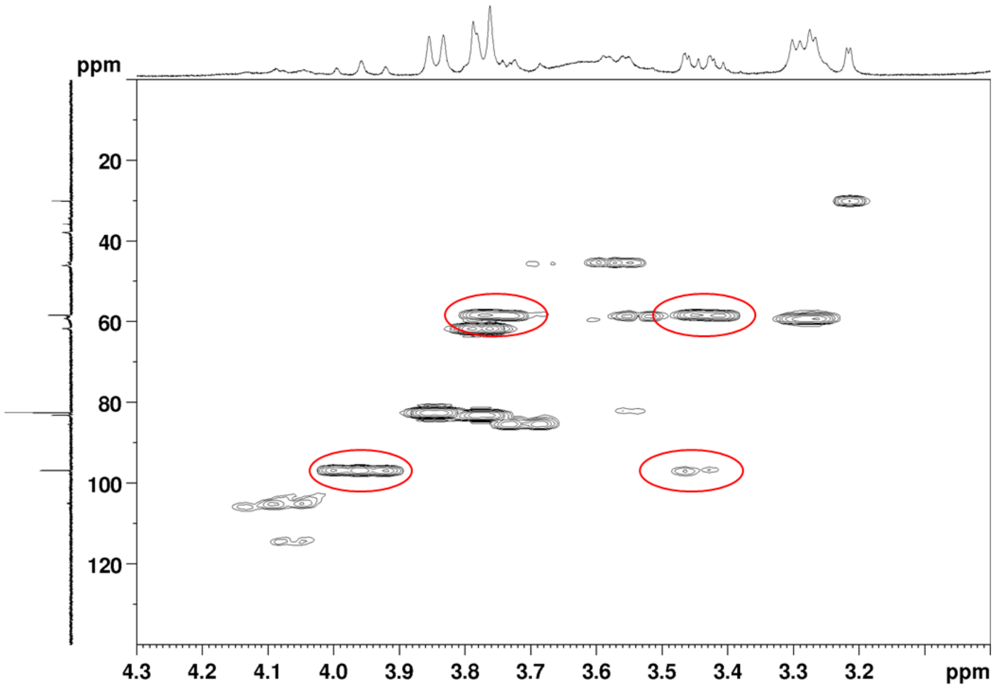

Figure S 14: Zoom of the $^{1}H,^{31}P$-HMBC NMR spectrum (400.17 MHz and 161.99 MHz, CD_2Cl_2, 243 K) of a reaction mixture containing **2** in the spectral region of the methylene groups. Signals belonging to **2** are marked.

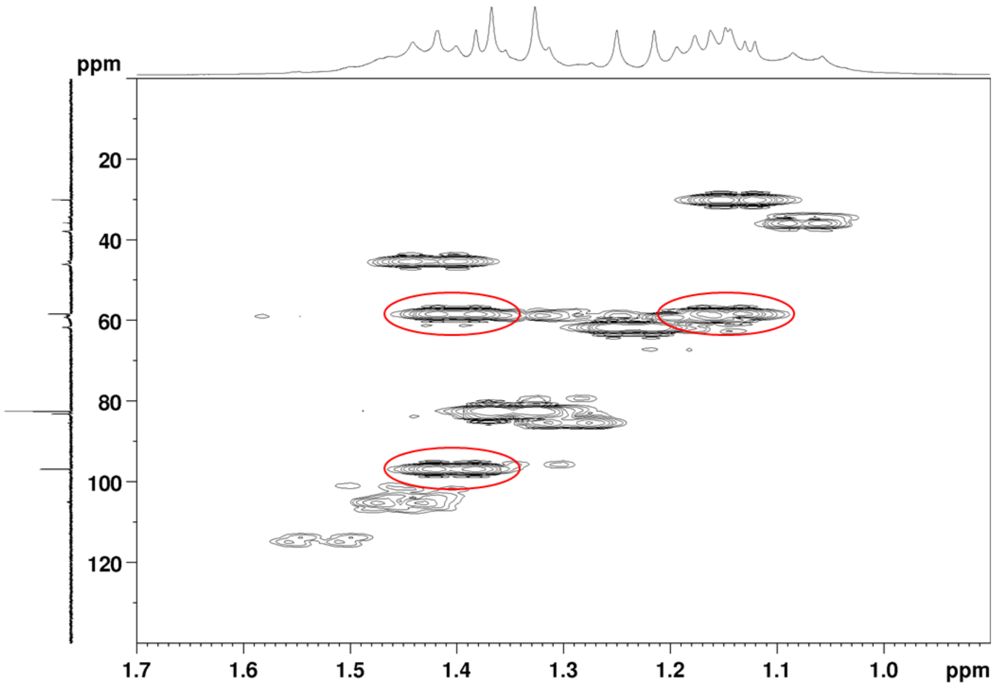

Figure S 15: Zoom of the $^{1}H,^{31}P$-HMBC NMR spectrum (400.17 MHz and 161.99 MHz, CD_2Cl_2, 243 K) of a reaction mixture containing **2** in the spectral region of the *tert*-butyl groups. Signals belonging to **2** are marked.

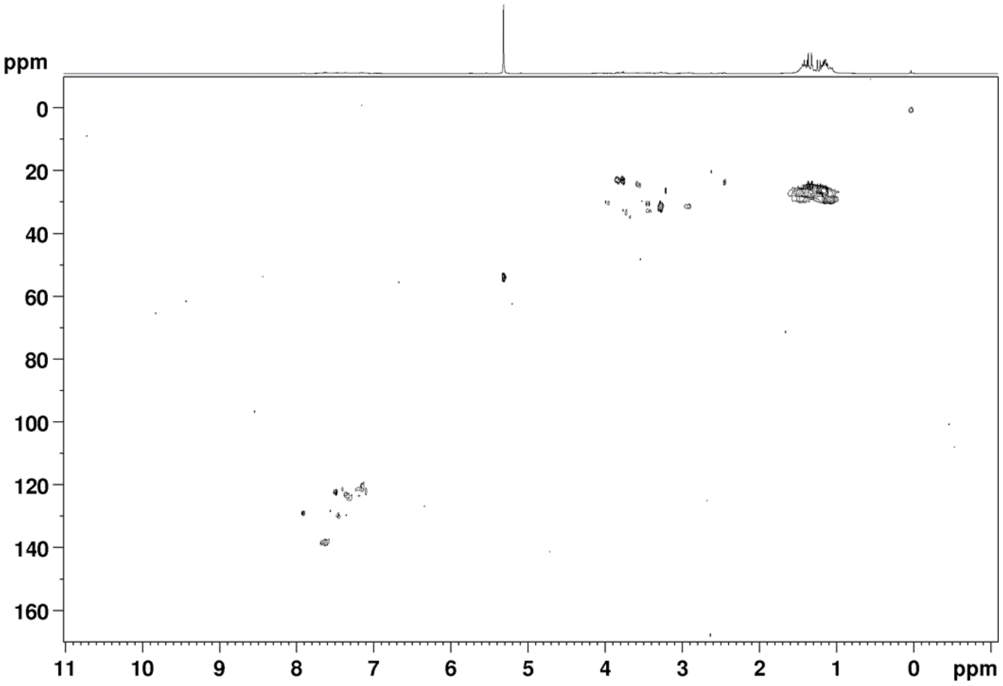

Figure S 16: $^{1}H,^{13}C$-HSQC NMR spectrum (400.17 MHz and 100.62 MHz, CD_2Cl_2, 243 K, optimised for J = 145 Hz) of a reaction mixture containing **2**.

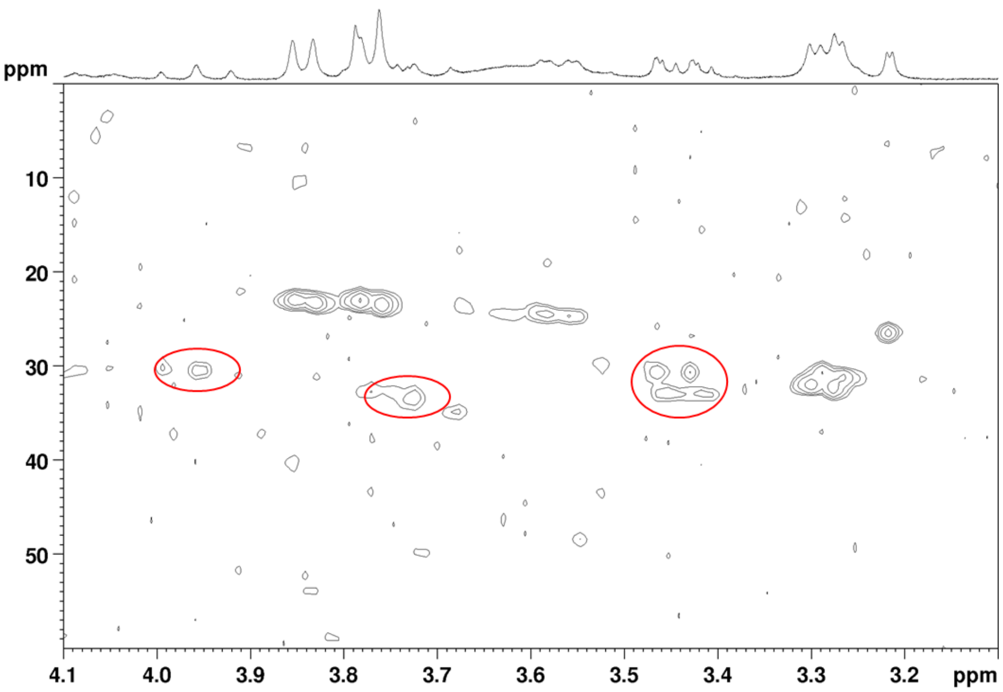

Figure S 17: Zoom of the ^{1}H,^{13}C-HSQC NMR spectrum (400.17 MHz and 100.62 MHz, CD_2Cl_2, 243 K) of a reaction mixture containing **2** in the spectral region of the methylene groups. Signals belonging to **2** are marked.

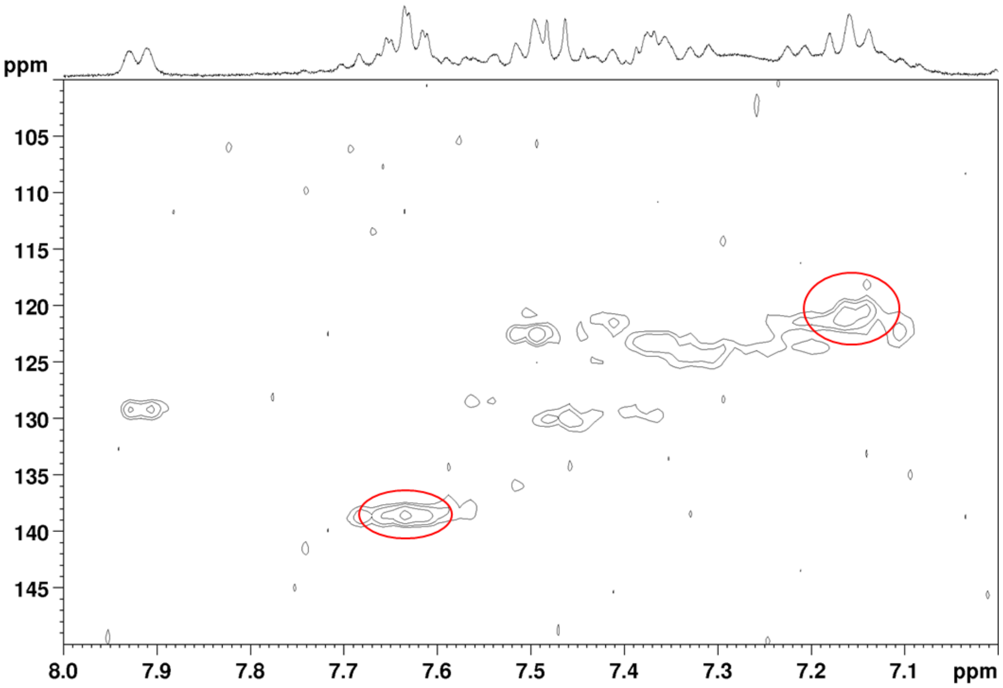

Figure S 18: Zoom of the ^{1}H,^{13}C-HSQC NMR spectrum (400.17 MHz and 100.62 MHz, CD_2Cl_2, 243 K) of a reaction mixture containing **2** in the spectral region of aromatic groups. Signals belonging to **2** are marked.

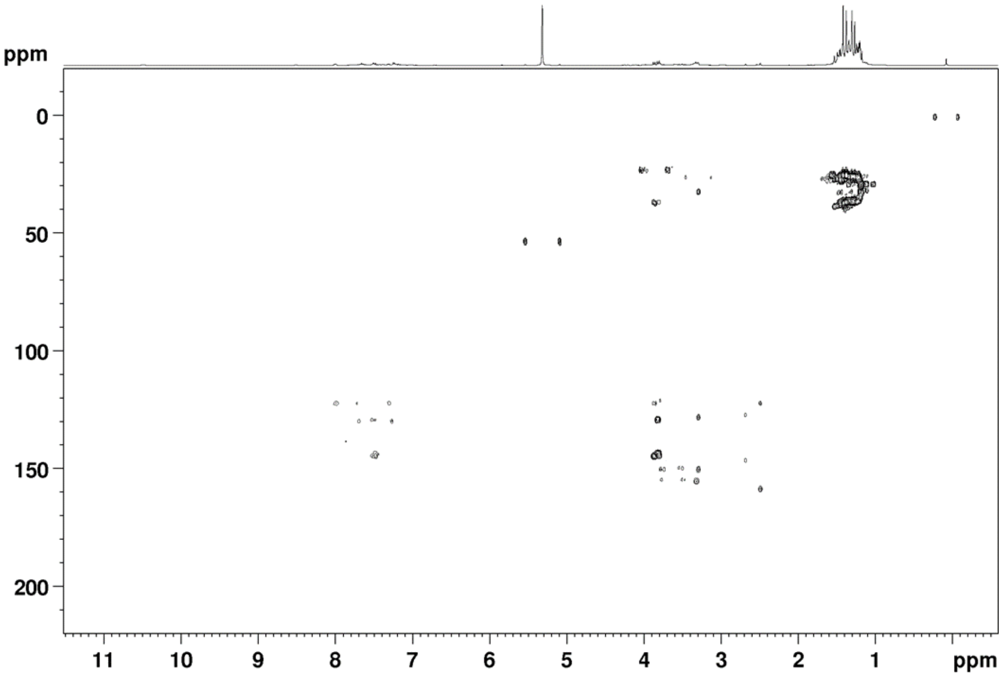

Figure S 19: ^{1}H,^{13}C-HMBC NMR spectrum (400.17 MHz and 100.62 MHz, CD_2Cl_2, RT, optimised for J = 8 Hz) of a reaction mixture containing **2**.

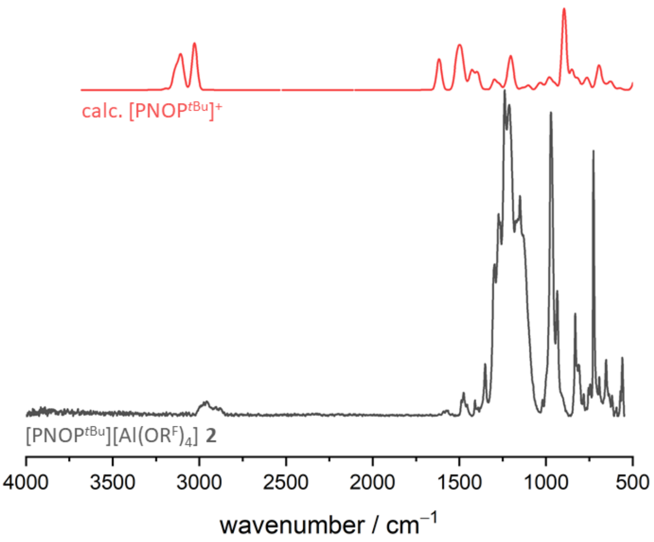

Figure S 20: IR spectrum (32 scans, ZnSe) of [PNOP^{tBu}][Al(OR^F)$_4$] **2** (black) as well as the calculated spectrum of [PNOP^{tBu}]$^+$ at the r2SCAN-3c/def2-mTZVPP level of theory (red).

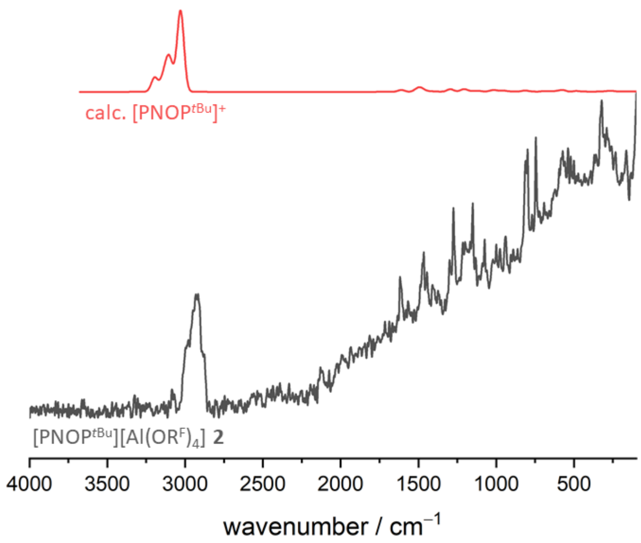

Figure S 21: Raman spectrum (1,000 scans, 50 mW) of [PNOP^{tBu}][Al(OR^F)$_4$] **2** (black) and calculated spectrum of [PNOP^{tBu}]$^+$ at the r2SCAN-3c/def2-mTZVPP level of theory (red).

Table S 2: Assignment of IR and Raman vibrations of [PNOP^{tBu}][Al(OR^F)$_4$] **2** and comparison with the calculated spectra for cation bands and with the reference compound [NEt$_4$][Al(OR^F)$_4$] for anion bands.[20] Due to the uneven baseline in the experimental Raman spectrum (caused by fluorescence), no Raman intensities are reported.

**1**				**[Al(OR^F)$_4$]$^-$**		**Assignment[a]**
**exp. IR**	**calc. IR**	**exp. Raman**	**calc. Raman**	**IR**	**Raman**	
		280			289 (w)	Anion, C–C
		322			323 (ms)	Anion, Al–O, C–C
		358			368 (w)	Anion, C–F, C–C
		521			538 (w)	Anion, C–O, C–C
559 (vw)				562 (mw)	563 (w)	Anion, Al–O, C–C
571 (vw)		572		571 (w)		Anion, Al–O, C–C
			574			–
593 (vw)	603					δ arene in plane
620 (vw)	616					ν P–C
654 (vw)	548					ν N–O
693 (vw)	692					ν N–O
727 (vs)				727 (s)		Anion, C–C, C–O
745 (vw)		745			747 (ms)	–
755 (vw)				756 (mw)		Anion, Al–O
772 (vw)	768					arene out of plane
		798			798 (s)	–
813 (vw)	814	810	816			ν C–C
832 (w)				833 (m)		Anion, Al–O, C–C
935 (w)	941					ν C–C
972 (vs)				973 (s)		Anion, C–C, C–F
			1015			arene in plane C–C, C–N
1020 (vw)	1024					δ H–C–H
1150 (s)		1152		1176 (ms)		Anion, C–C, C–F
			1204			δ H–C–H
1212 (vs)				1217 (vs)		Anion, C–C, C–F
1239 (vs)				1240 (s)		Anion, C–C, C–F
1274 (s)		1275		1274 (vs)	1274 (mw)	Anion, C–C, C–F
			1296			arene in plane C–C, C–H
1297 (m)		1299		1298 (s)	1300 (m)	Anion, C–C, C–F
1351 (vw)				1353 (ms)		Anion, C–C, C–F
1410 (vw)	1421					δ H–C–H
1455 (vw)	1456					arene in plane C–C, C–H
		1466	1486			δ H–C–H
1475 (vw)	1493					δ H–C–H
1576 (vw)	1621					arene in plane C–C, C–H
		1618	1607			arene in plane C–C, C–H, C–N
2903(vw)	3023					ν C–H
		2924	3034			
2958 (vw)	3111					ν C–H
		2979	3108			ν C–H
		3085	3190			ν arene C–H

[a] From a visualization of the calculated spectra.

4. NMR Spectroscopy and Kinetic Analysis

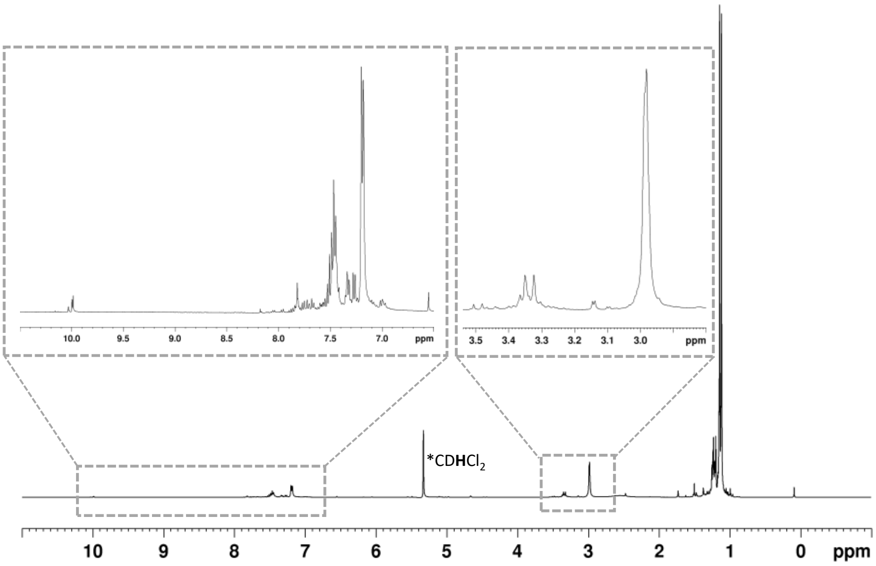

Figure S 22: ^{1}H NMR spectrum (400.17 MHz, CD_2Cl_2, RT) of PNP^{tBu} after being oxidised on air for 4 days.

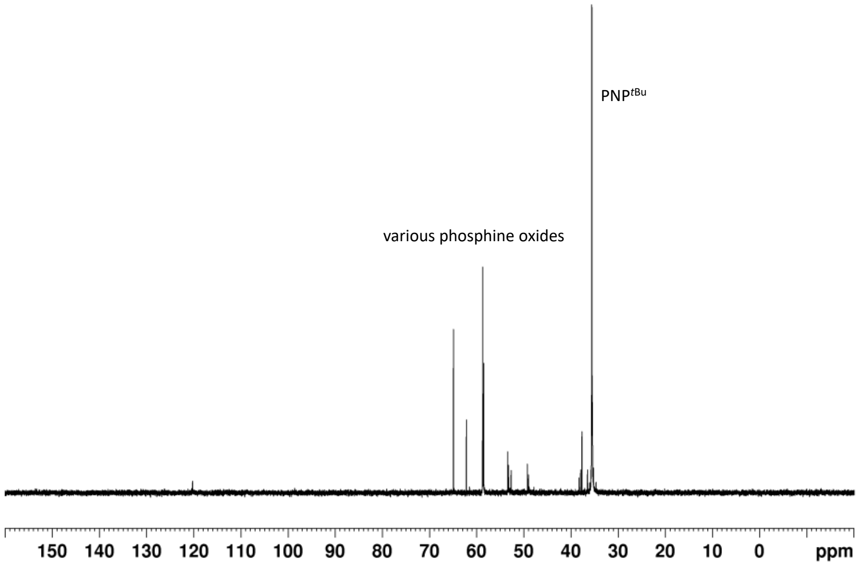

Figure S 23: $^{31}P\{^{1}H\}$ NMR spectrum (161.99 MHz, CD_2Cl_2, RT) of PNP^{tBu} after being oxidised on air for 4 days.

Kinetic Analysis of the conversion of **2** to **1** at RT

$NO[Al(OR^F)_4]$ (15 mg, 15 µmol) and PNP^{tBu} (5.9 mg, 15 µmol, 1.0 equiv) were weighed into a J. Young NMR tube and dissolved in CD_2Cl_2 (0.6 mL) which was pre-cooled to −30 °C. The NMR tube was shaken multiple times and put back into the cooling bath to ensure that it stayed at < −30 °C all the time. The sample was then measured in a pre-cooled NMR spectrometer at −30 °C. The first spectra of measurements at −30 °C, −20 °C, −10 °C, 10 °C and RT are shown in Figure S 24 to Figure S 28. At each temperature, one or multiple spectra were collected in regular intervals. For the kinetic analysis, the low-field signals of **1** and **2** in the $^{31}P\{^1H\}$ NMR spectrum at 82.7 ppm and 97.6 ppm respectively were used. For this, the signal of **2** in the first measured spectrum at T = 243 K and t = 0 was used as a reference and all other $^{31}P\{^1H\}$ signal integrals in all following spectra are referenced and calibrated against this signal. All integrals are summarized in Table S 3 and Table S 4.

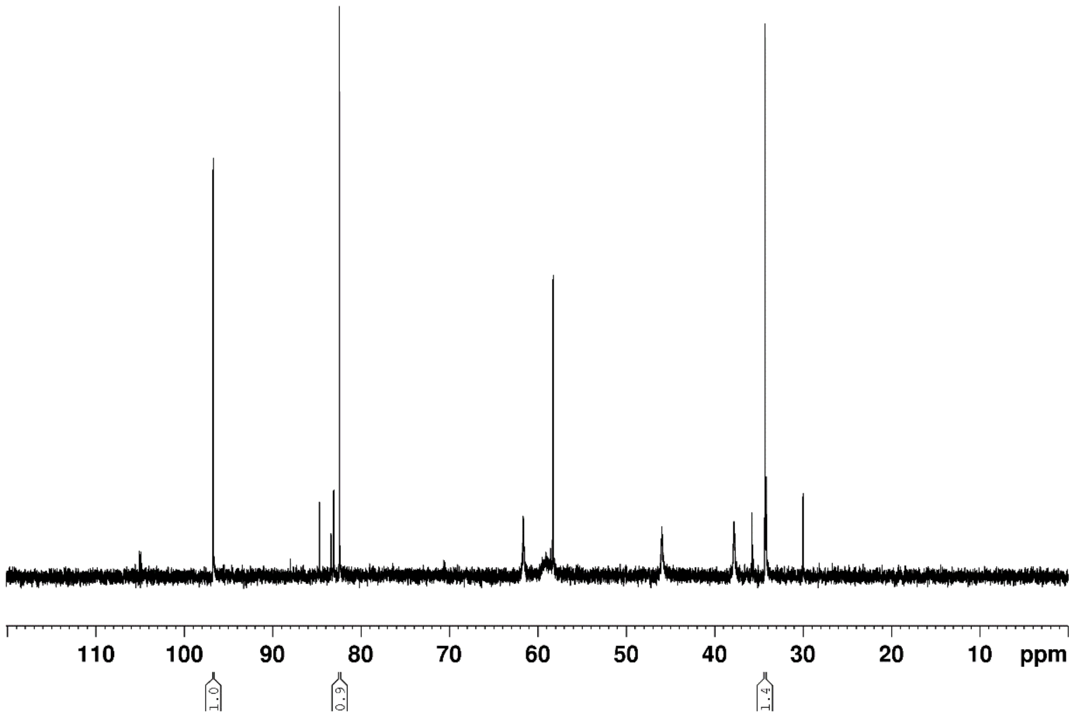

Figure S 24: $^{31}P\{^1H\}$ NMR spectrum (161.99 MHz, CD_2Cl_2, 243 K) at t = 0. Integrals given for the low-field signals of **2** (1.0) and **1** (0.9) as well as for free PNP^{tBu}.

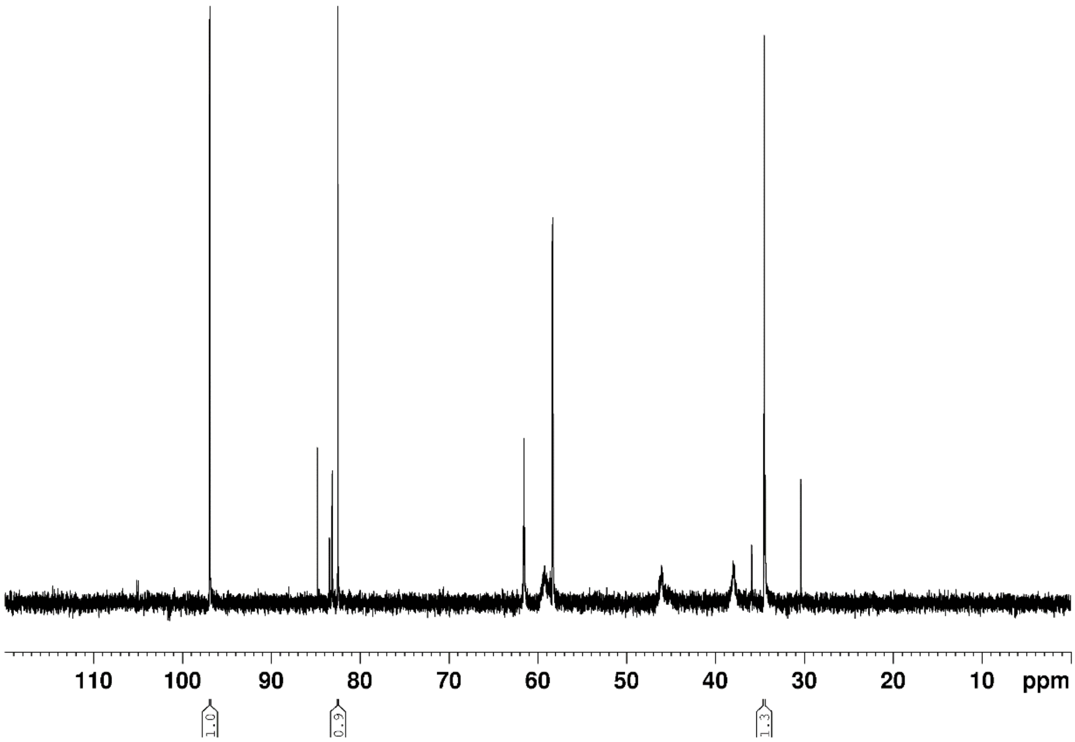

Figure S 25: $^{31}P\{^{1}H\}$ NMR spectrum (161.99 MHz, CD_2Cl_2, 253 K) at *t* = 0.

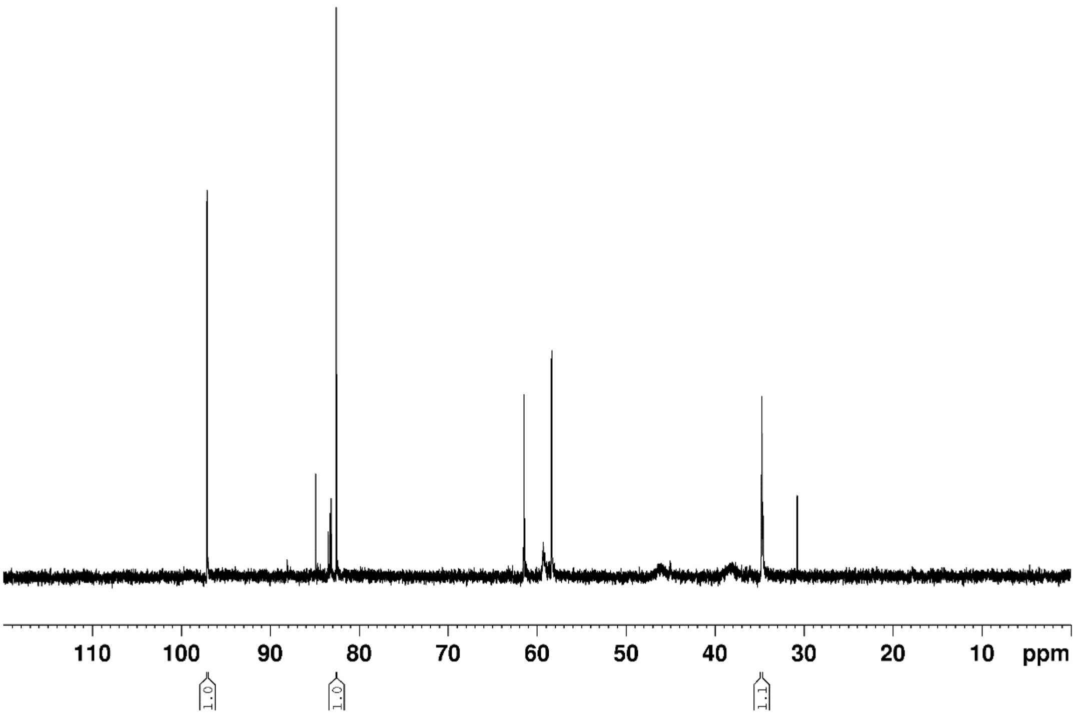

Figure S 26: $^{31}P\{^{1}H\}$ NMR spectrum (161.99 MHz, CD_2Cl_2, 263 K) at *t* = 0.

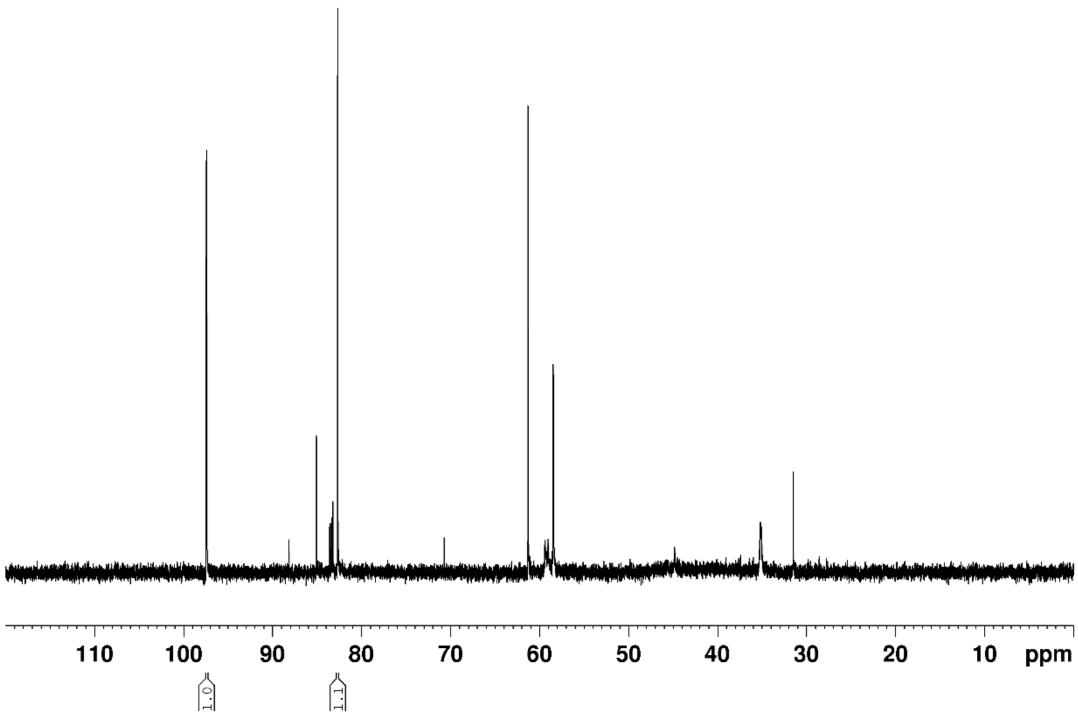

Figure S 27: $^{31}P\{^{1}H\}$ NMR spectrum (161.99 MHz, CD_2Cl_2, 283 K) at t = 0.

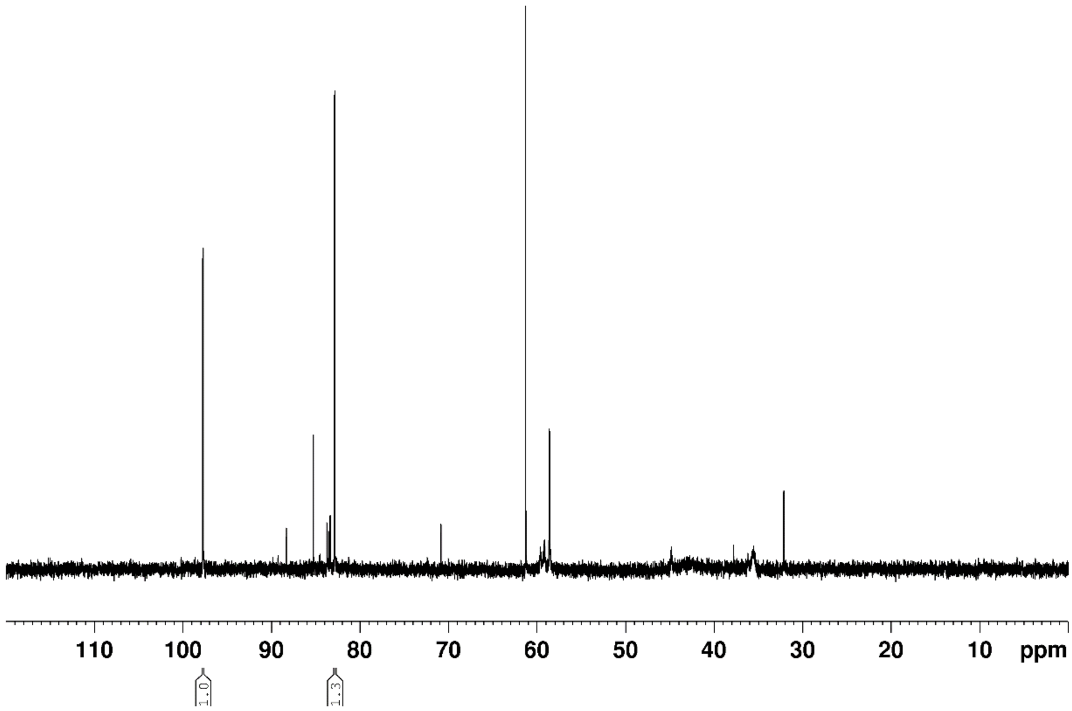

Figure S 28: $^{31}P\{^{1}H\}$ NMR spectrum (161.99 MHz, CD_2Cl_2, RT) at t = 0.

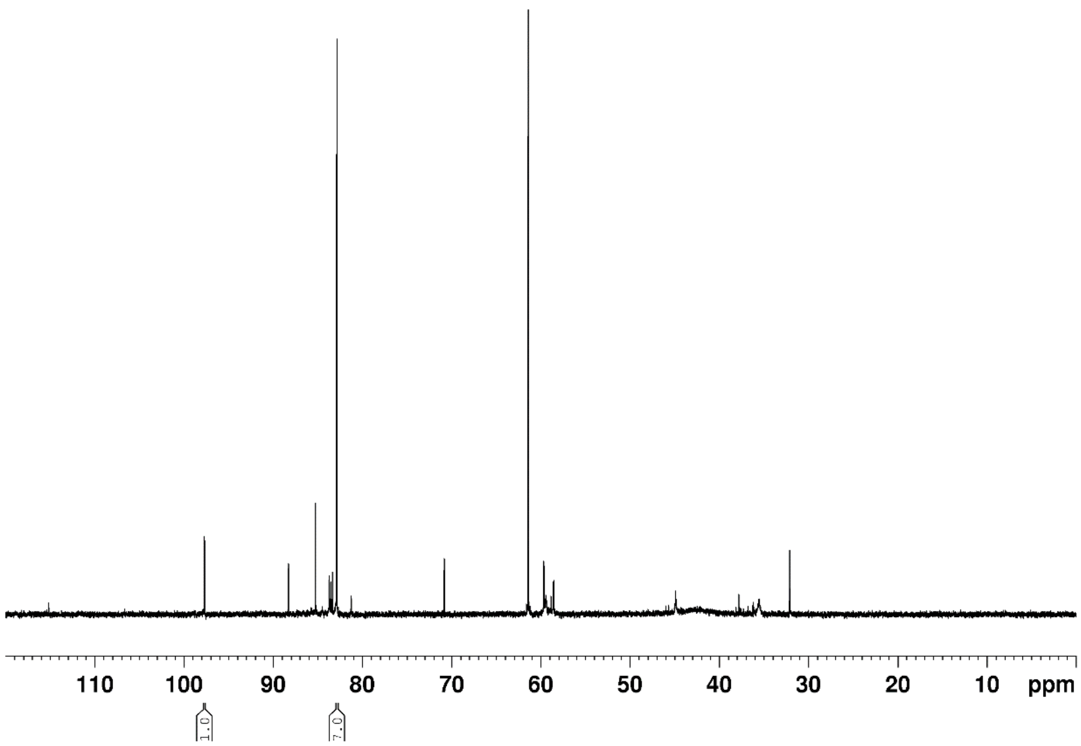

Figure S 29: $^{31}P\{^1H\}$ NMR spectrum (161.99 MHz, CD_2Cl_2, RT) at *t* = 1688 min (2 d).

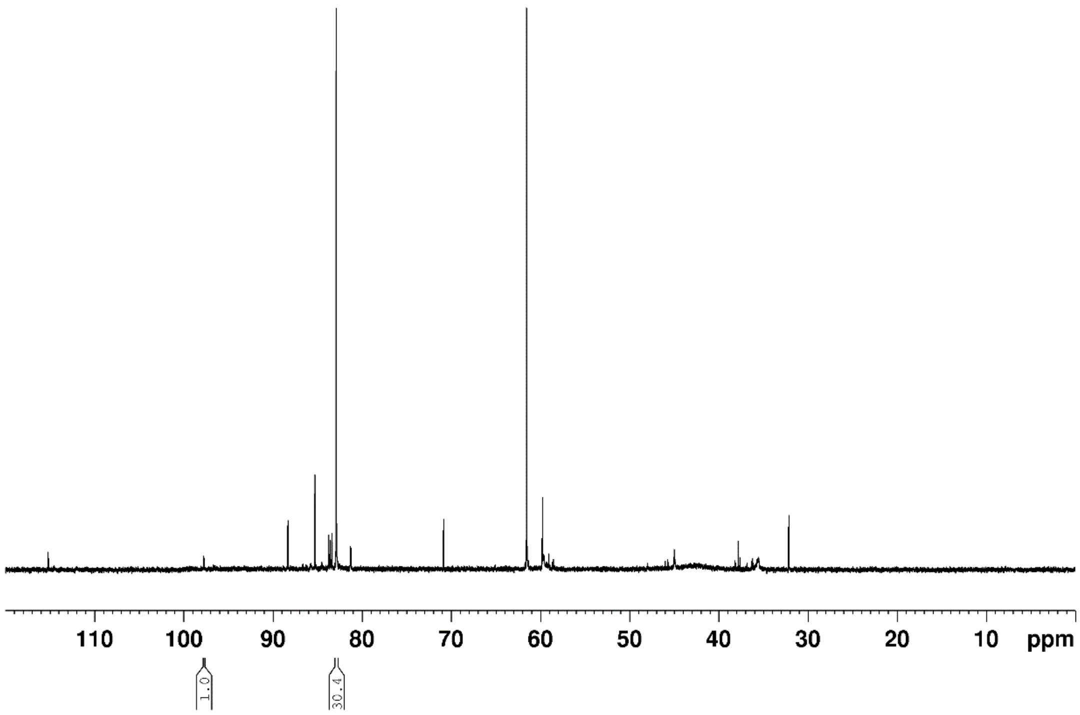

Figure S 30: $^{31}P\{^1H\}$ NMR spectrum (161.99 MHz, CD_2Cl_2, RT) at *t* = 4186 min (3.8 d).

Table S 3: Integrals of the low-field signals of **1** and **2** in the $^{31}P\{^1H\}$ NMR spectrum at different temperatures *T* (*T* = 243 K, 253 K, 263 K, 283 K) and at different times *t* at the given temperature. All integrals are referenced against the low-field signal of **2** at *T* = 243 K and *t* = 0 (grey) which was arbitrarily set 1.0000.

Temperature *T* / K	Time *t* / min (at a given *T*)	Integral[a] of low-field signal of **2** at 97.6 ppm	Integral[a] of low-field signal of **1** at 82.7 ppm
243	0	1.0000	0.9557
243	178	1.1275	1.0970
253	0	1.1370	1.1110
253	35	1.1239	1.1122
263	0	1.0082	1.0760
283	0	0.8696	0.9688
283	47	0.8863	0.9589
283	94	0.8386	0.9393
283	140	0.8654	1.0031
283	338	0.8459	1.0027
283	384	0.8236	1.0120
283	430	0.8557	1.0478
283	493	0.8362	1.0683
283	660	0.8464	1.0427

[a] relative to the spectrum at *T* = 243 K and *t* = 0 (grey background)

Table S 4: Integrals of the low-field signals of **1** and **2** in the $^{31}P\{^1H\}$ NMR spectrum at *T* = 298 K (RT) at different times *t*. All integrals are referenced against the low-field signal of **2** at *T* = 243 K and *t* = 0 (grey, Table S 3) which was arbitrarily set 1.0000. The sums of both integrals are given and remain constant inside the error. The integral for **1** was corrected by subtracting the first integral of **1** at *T* = 243 K and *t* = 0 (0.9557, grey, Table S 3) to account for the presence of **1** in the reaction mixture before the first measurement.

Time *t* / min	Integral[a] of low-field signal of **2** at 97.6 ppm	Integral[a] of low-field signal of **1** at 82.7 ppm	Sum of integrals	Corrected[b] integral of low-field signal of **1** at 82.7 ppm
0	0.7099	0.9776	1.6875	0.0219
46	0.6784	0.9712	1.6496	0.0155
93	0.6751	0.9722	1.6473	0.0165
152	0.6627	1.0150	1.6777	0.0593
201	0.6188	1.0025	1.6213	0.0468
247	0.4077	0.6756	1.0833	−0.2801
293	0.5722	1.0065	1.5787	0.0508
351	0.5704	1.0204	1.5908	0.0647
398	0.5595	1.0316	1.5911	0.0759
444	0.5130	1.0455	1.5585	0.0898
542	0.4885	1.0613	1.5498	0.1056
589	0.4730	1.1598	1.6327	0.2041
725	0.0957	0.2381	0.3338	−0.7176
862	0.4031	1.2099	1.6129	0.2542
998	0.3325	1.2404	1.5728	0.2847
1134	0.3178	1.2829	1.6006	0.3272
1270	0.2958	1.3253	1.6211	0.3696
1407	0.2622	1.3537	1.6159	0.3980
1543	0.2503	1.3688	1.6191	0.4131
1688	0.2083	1.3679	1.5762	0.4122
1824	0.1943	1.3936	1.5879	0.4379
2004	0.1707	1.3854	1.5562	0.4297
2141	0.1613	1.4247	1.5859	0.4690
2277	0.1488	1.4287	1.5775	0.4730
2414	0.1326	1.4519	1.5845	0.4962

2550	0.1157	1.4724	1.5881	0.5167
2686	0.1070	1.4854	1.5924	0.5297
2823	0.0983	1.4904	1.5887	0.5247
2959	0.0882	1.5088	1.5970	0.5531
3095	0.0953	1.5258	1.6211	0.5701
3232	0.0643	1.5118	1.5760	0.5561
3368	0.0168	0.2935	0.3102	−0.6623
3504	0.0562	1.5658	1.6220	0.6101
3641	0.0596	1.5314	1.5910	0.5757
3777	0.0620	1.5498	1.6117	0.5941
3914	0.0516	1.5465	1.5981	0.5908
4050	0.0356	1.5483	1.5838	0.5926
4186	0.0567	1.5996	1.6563	0.6439

[a] relative to the spectrum at T = 243 K and t = 0 in Table S 3

[b] To account for the presence of **1** in the reaction mixture before the first measurement, the first relative integral (0.9557, see Table S 3) was subtracted from the following integrals.

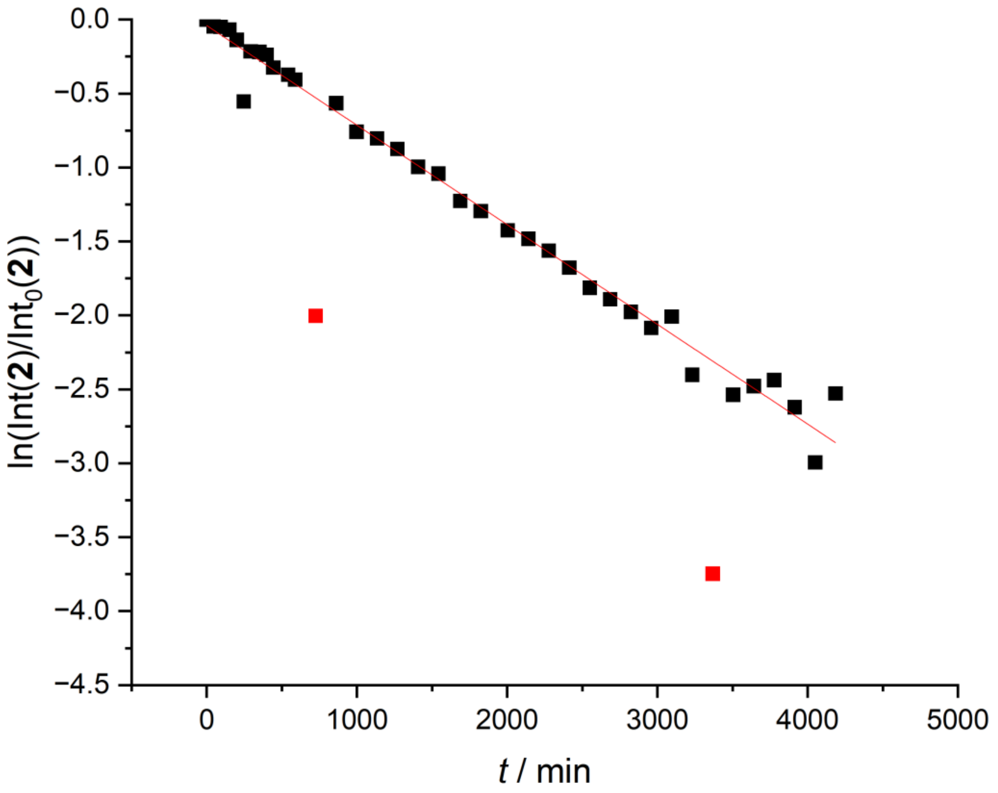

Figure S 31: Plot of the logarithm of the integral of **2** at a certain time t divided by the initial integral of **2** against the time t at RT. A linear regression of the type $\ln(\mathrm{Int}(\mathbf{2})/\mathrm{Int}_0(\mathbf{2})) = a + k \cdot t$ was performed (R^2 = 0.99) yielding a = −0.04 ± 0.03 and k = −(6.74 ± 0.14) · 10^{-4} min^{-1} with k being the rate constant for a first order reaction. Red data points are not considered for the regression. At larger t the data is more scattered due to very small quantities of **2** being present in the reaction mixture and thus more unreliable integration of signals.

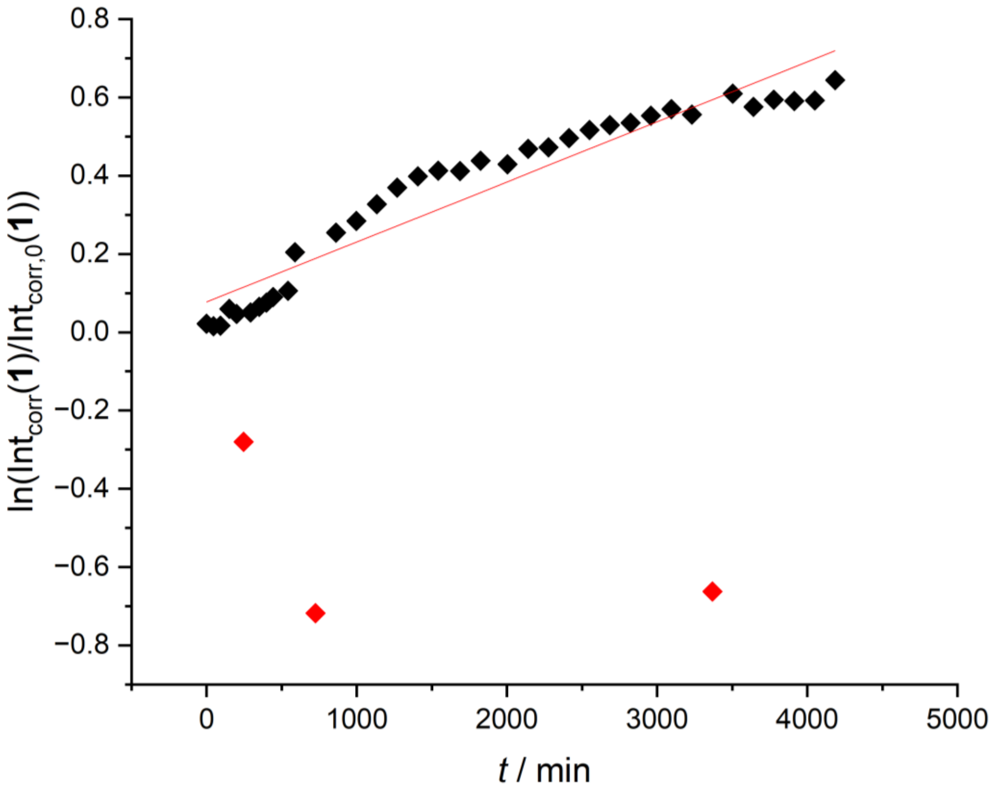

Figure S 32: Plot of the logarithm of the corrected integral of **1** at a certain time *t* divided by the initial corrected integral of **1** against the time *t* at RT. A linear regression of the type ln(Int$_{corr}$(**1**)/ Int$_{corr,0}$(**1**)) = a + k · *t* was performed (R^2 = 0.91) yielding a = 0.077 ± 0.019 and k = (1.53 ± 0.08) · 10^{-4} min^{-1} with k being the rate constant for a first order reaction. Red data points are not considered for the regression.

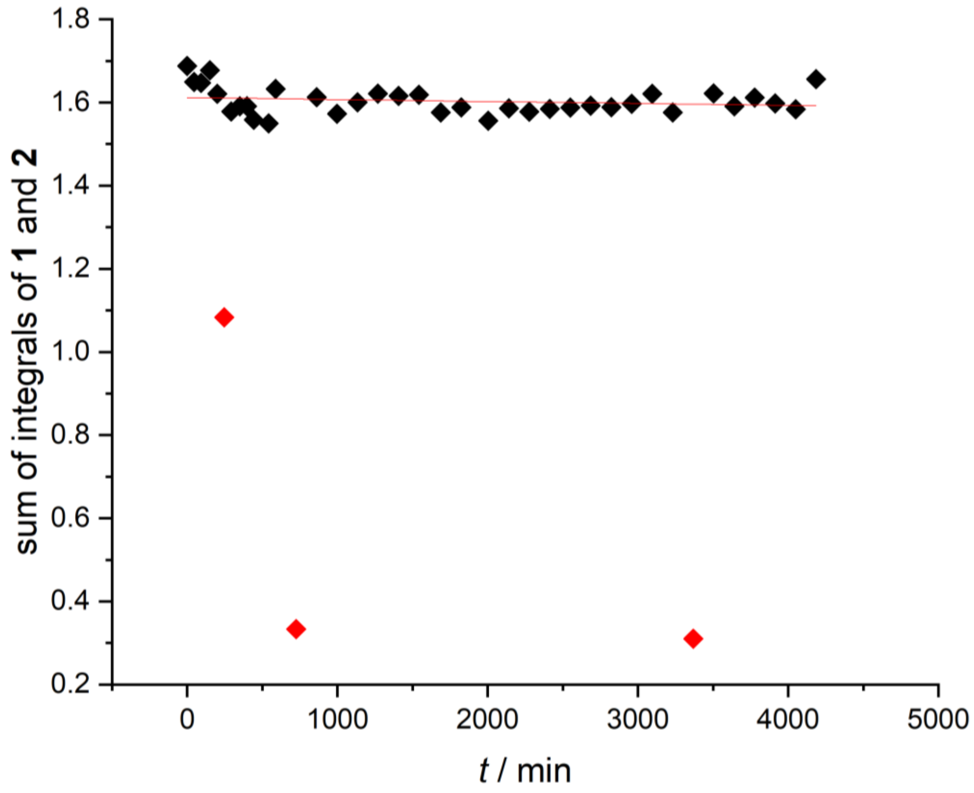

Figure S 33: Sum of the integrals of the low-field ^{31}P-signals of **1** and **2** at different times *t* at RT. A linear regression with a slope of −5 · 10^{-6} shows that the sum of integrals remains relatively constant over time which supports a conversion of **2** to **1**. Red data points are not considered for the regression.

5. Single-Crystal XRD Data

Table S 5: Crystallographic data of [DAPP^{tBu}][Al(OR^F)$_4$] **1** and [PNOP^{tBu}][Al(OR^F)$_4$] **2**. **1** was refined as a 2-component twin. **2** was refined as a 4-component inversion twin.

	**1**	**2**
**CCDC number**	2497214	2497215
**Empirical formula**	$C_{39}H_{43}AlF_{36}N_2O_5P_2$	$C_{39}H_{43}AlF_{36}N_2O_5P_2$
**Formula weight**	1392.67	1392.67
**Temperature [K]**	100(2)	100(2)
**Crystal system**	monoclinic	monoclinic
**Space group (number)**	$P2_1/c$ (14)	$P2_1$ (4)
a **[Å]**	17.307(9)	10.161(5)
b **[Å]**	15.025(7)	23.277(8)
c **[Å]**	20.918(11)	11.428(4)
**α [°]**	90	90
**β [°]**	92.664(13)	90.683(18)
**γ [°]**	90	90
**Volume [Å^3]**	5433(5)	2702.6(18)
Z	4	2
**ρ_{calc} [g cm^{-3}]**	1.702	1.711
**μ [mm^{-1}]**	0.264	0.265
***F*(000)**	2792	1396
**Crystal size [mm^3]**	0.131×0.150×0.217	0.122×0.264×0.536
**Crystal colour**	colourless	colourless
**Crystal shape**	block	block
**Radiation**	MoK_α (λ=0.71073 Å)	MoK_α (λ=0.71073 Å)
**2θ range [°]**	1.95 to 52.82 (0.80 Å)	4.37 to 50.38 (0.83 Å)
**Index ranges**	−21 ≤ h ≤ 21	−11 ≤ h ≤ 12
	−18 ≤ k ≤ 18	−27 ≤ k ≤ 27
	−26 ≤ l ≤ 26	−13 ≤ l ≤ 13
**Reflections collected**	92781	32211
**Independent reflections**	11142	9547
	R_{int} = 0.0577	R_{int} = 0.0960
	R_{sigma} = 0.0340	R_{sigma} = 0.0916
**Completeness to θ = 25.242° / 25.188°**	100.0 %	99.6 %
**Data / Restraints / Parameters**	11142 / 11583 / 1287	9547 / 9160 / 1180
**Absorption correction T_{min}/T_{max} (method)**	0.7187 / 0.7454 (multi-scan)	0.5849 / 0.7452 (multi-scan)
**Goodness-of-fit on *F*^{2}**	1.087	1.268
**Final *R* indexes [*I* ≥ 2σ(*I*)]**	R_1 = 0.0741	R_1 = 0.1203
	wR_2 = 0.1867	wR_2 = 0.3062
**Final *R* indexes [all data]**	R_1 = 0.0970	R_1 = 0.1650
	wR_2 = 0.2008	wR_2 = 0.3467
**Largest peak/hole [eÅ^{-3}]**	0.94/−0.53	0.71/−0.49

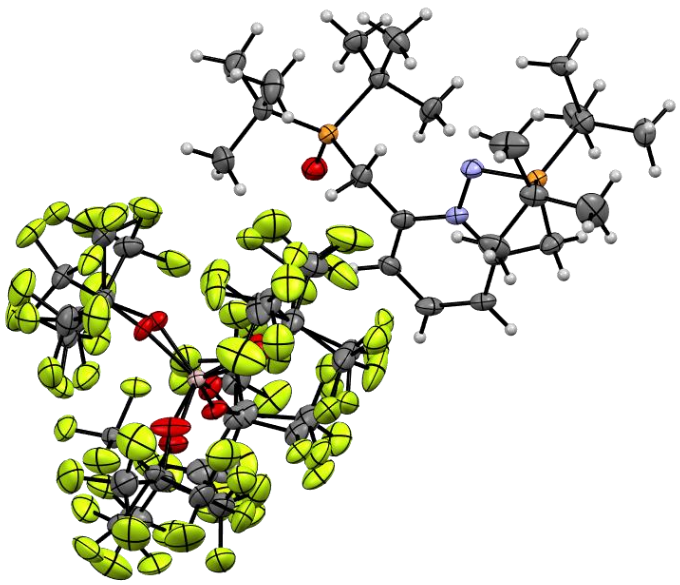

Figure S 34: Molecular structure of [DAPP^{tBu}][Al(OR^F)$_4$] **1** ($P2_1/c$, R_1 = 7.4 %, wR_2 = 20.1 %) with disorders of the anion shown. Thermal ellipsoids are set at the 50 % probability level.

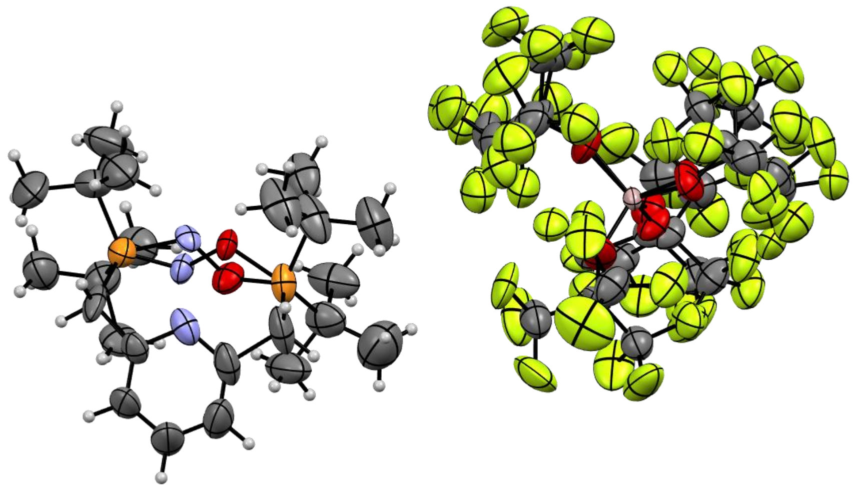

Figure S 35: Molecular structure of [PNOP^{tBu}][Al(OR^F)$_4$] **2** ($P2_1$, R_1 = 12.0 %, wR_2 = 34.7 %) with disorders of the anion and the $(NO)^+$ moiety shown. Thermal ellipsoids are set at the 50 % probability level.

Details on the structure of **2**

Crystallisation of **2** was attempted using different concentrations and solvents (DCM or 4FB). The result was always the same: Very thin crystalline plates that diffract very poorly (resolution of 1.00 Å with an exposure time of 60 s). Our best data stems from a larger crystal consisting of multiple plates sticking together and leading to multiple domains (resolution of 0.84 Å with an exposure time of 60 s). We integrated the main domain separately and together with the second largest domain as a twin, but integration of only the main domain yielded the best results. The twinning was then addressed later during the refinement as a 4-component inversion twin. This treatment led to a solution in the non-centrosymmetric space group $P2_1$ and together with the bad diffraction and high degree of disorder to our poor data/parameter ratio and high R_1/wR_2 values. We decided to include the structure nonetheless in our manuscript as the connectivity of **2** can be unambiguously determined and is well in line with NMR spectroscopic data and quantum chemical calculations.

Atom Coordinates, Bond Lengths, Bond Angles

Table S 6: Atomic coordinates and U_{eq} [Å^2] for [DAPP^{tBu}][Al(OR^F)$_4$] **1**.

Atom	*x*	*y*	*z*	U_{eq}
P1	0.24653(6)	0.24895(7)	0.87045(5)	0.0239(2)
P2	0.39060(6)	0.32892(7)	0.63567(5)	0.0254(2)
Al1	0.18890(6)	0.79035(7)	0.64422(5)	0.0187(2)
O1	0.34470(19)	0.3149(2)	0.57529(15)	0.0388(8)
N1	0.28922(19)	0.2867(2)	0.80730(17)	0.0274(7)
C1	0.1505(2)	0.2320(3)	0.8327(2)	0.0326(10)
H1A	0.137341	0.167898	0.829930	0.039
H1B	0.110362	0.263377	0.856317	0.039
C2	0.3318(3)	0.3754(3)	0.6992(2)	0.0312(9)
H2A	0.359626	0.364980	0.740956	0.037
H2B	0.328181	0.440629	0.692974	0.037
N1_5	0.23252(18)	0.2998(2)	0.75873(16)	0.0245(7)
C1_5	0.1587(2)	0.2714(3)	0.76776(19)	0.0249(8)
C2_5	0.1023(2)	0.2794(3)	0.7197(2)	0.0285(9)
H2_5	0.051302	0.258917	0.726068	0.034
C3_5	0.1203(2)	0.3175(3)	0.6619(2)	0.0351(10)
H3_5	0.082015	0.322759	0.628061	0.042
C4_5	0.1946(2)	0.3475(3)	0.6542(2)	0.0344(10)
H4_5	0.207045	0.374326	0.614869	0.041
C5_5	0.2515(2)	0.3394(3)	0.7029(2)	0.0284(9)
C1_6	0.3254(3)	0.3642(4)	0.9528(3)	0.0557(15)
H1A_6	0.355950	0.373707	0.915038	0.084
H1B_6	0.349415	0.317278	0.979448	0.084
H1C_6	0.323605	0.419558	0.977463	0.084
C2_6	0.2432(3)	0.3364(3)	0.9314(2)	0.0360(10)
C3_6	0.2017(3)	0.4156(3)	0.8979(3)	0.0469(13)
H3A_6	0.229682	0.432778	0.860119	0.070
H3B_6	0.200373	0.466008	0.927524	0.070
H3C_6	0.148774	0.398268	0.884709	0.070
C4_6	0.1983(4)	0.3071(4)	0.9894(3)	0.0594(16)
H4A_6	0.149369	0.279508	0.974577	0.089
H4B_6	0.187573	0.359150	1.015843	0.089
H4C_6	0.229183	0.264075	1.014780	0.089
C1_7	0.2832(4)	0.0801(3)	0.8368(2)	0.0497(14)
H1A_7	0.228618	0.067284	0.825963	0.075
H1B_7	0.310695	0.024318	0.846417	0.075
H1C_7	0.306301	0.109495	0.800456	0.075
C2_7	0.2894(2)	0.1410(3)	0.8951(2)	0.0301(9)
C3_7	0.3748(2)	0.1546(3)	0.9154(2)	0.0380(11)
H3A_7	0.400442	0.096553	0.919581	0.057
H3B_7	0.378701	0.185609	0.956674	0.057
H3C_7	0.399910	0.190185	0.883092	0.057
C4_7	0.2466(3)	0.0992(3)	0.9500(2)	0.0426(12)
H4A_7	0.190790	0.100962	0.939913	0.064
H4B_7	0.258458	0.132612	0.989510	0.064
H4C_7	0.263272	0.037274	0.955813	0.064
C1_8	0.5280(3)	0.3851(3)	0.5853(3)	0.0413(11)
H1A_8	0.504986	0.357051	0.546655	0.062
H1B_8	0.559891	0.341516	0.609342	0.062
H1C_8	0.560261	0.435413	0.573239	0.062

C2_8	0.4643(2)	0.4181(3)	0.6267(2)	0.0311(9)
C3_8	0.4213(3)	0.4930(4)	0.5895(3)	0.0535(14)
H3A_8	0.457664	0.540986	0.580767	0.080
H3B_8	0.379813	0.516180	0.615133	0.080
H3C_8	0.399132	0.469411	0.549020	0.080
C4_8	0.4981(3)	0.4546(4)	0.6900(2)	0.0488(13)
H4A_8	0.536035	0.501104	0.681621	0.073
H4B_8	0.523562	0.406351	0.714532	0.073
H4C_8	0.456555	0.479744	0.714659	0.073
C1_9	0.4779(3)	0.1772(3)	0.6157(2)	0.0384(11)
H1A_9	0.450562	0.181283	0.573705	0.058
H1B_9	0.485934	0.114495	0.627000	0.058
H1C_9	0.528128	0.207118	0.614093	0.058
C2_9	0.4296(2)	0.2223(3)	0.6662(2)	0.0278(9)
C3_9	0.4779(3)	0.2294(3)	0.7291(2)	0.0446(12)
H3A_9	0.492139	0.169587	0.744223	0.067
H3B_9	0.447657	0.259303	0.761196	0.067
H3C_9	0.524905	0.263751	0.722133	0.067
C4_9	0.3585(3)	0.1633(3)	0.6758(3)	0.0409(12)
H4A_9	0.325427	0.162927	0.636436	0.061
H4B_9	0.329287	0.186864	0.711064	0.061
H4C_9	0.375370	0.102435	0.685912	0.061
O1_10	0.1258(3)	0.8794(3)	0.6359(3)	0.0365(11)
C1_10	0.1067(3)	0.9616(3)	0.6542(3)	0.0264(10)
C2_10	0.0566(3)	1.0043(5)	0.5984(3)	0.0451(12)
F1_10	−0.0143(2)	0.9701(4)	0.5957(3)	0.0695(14)
F2_10	0.0506(3)	1.0929(3)	0.6056(3)	0.0686(13)
F3_10	0.0866(3)	0.9907(4)	0.5422(2)	0.0562(11)
C3_10	0.1808(3)	1.0192(3)	0.6656(3)	0.0305(10)
F4_10	0.23400(18)	0.9764(2)	0.70148(16)	0.0344(8)
F5_10	0.2125(2)	1.0384(3)	0.61117(18)	0.0484(10)
F6_10	0.1680(2)	1.0953(3)	0.6958(3)	0.0573(12)
C4_10	0.0610(4)	0.9625(5)	0.7155(3)	0.0497(13)
F7_10	0.1076(3)	0.9462(5)	0.7668(2)	0.0787(17)
F8_10	0.0250(3)	1.0400(4)	0.7252(3)	0.0801(16)
F9_10	0.0076(3)	0.9004(4)	0.7135(3)	0.0788(16)
O1_11	0.1512(9)	0.8904(11)	0.6531(9)	0.045(3)
C1_11	0.0925(8)	0.9502(9)	0.6516(6)	0.039(2)
C2_11	0.0498(9)	0.9537(10)	0.5847(7)	0.052(3)
F1_11	0.0026(8)	0.8851(10)	0.5748(8)	0.072(4)
F2_11	0.0111(12)	1.0299(11)	0.5753(9)	0.086(5)
F3_11	0.0994(8)	0.9351(12)	0.5419(6)	0.058(3)
C3_11	0.1196(8)	1.0445(9)	0.6710(7)	0.049(3)
F4_11	0.1718(10)	1.0406(12)	0.7186(8)	0.079(4)
F5_11	0.1548(9)	1.0868(10)	0.6253(8)	0.075(4)
F6_11	0.0585(9)	1.0930(10)	0.6872(9)	0.079(4)
C4_11	0.0338(8)	0.9169(10)	0.7001(7)	0.048(3)
F7_11	0.0628(9)	0.9248(11)	0.7594(6)	0.057(3)
F8_11	−0.0331(7)	0.9573(10)	0.6940(7)	0.064(4)
F9_11	0.0239(9)	0.8299(8)	0.6921(8)	0.069(4)
O1_12	0.2810(3)	0.8249(3)	0.6247(3)	0.0243(11)
C1_12	0.3364(3)	0.8255(3)	0.5814(2)	0.0226(9)
C2_12	0.3939(3)	0.9025(4)	0.6000(2)	0.0312(10)
F1_12	0.3641(3)	0.9801(3)	0.5802(2)	0.0510(11)
F2_12	0.4615(2)	0.8920(3)	0.57206(19)	0.0527(11)

F3_12	0.4083(2)	0.9064(3)	0.66186(15)	0.0434(9)
C3_12	0.3804(3)	0.7360(4)	0.5837(3)	0.0416(12)
F4_12	0.3300(2)	0.6687(2)	0.5857(2)	0.0588(11)
F5_12	0.4278(3)	0.7320(3)	0.6366(2)	0.0555(12)
F6_12	0.4233(3)	0.7237(3)	0.5338(2)	0.0730(14)
C4_12	0.3013(4)	0.8418(4)	0.5129(3)	0.0376(12)
F7_12	0.2717(3)	0.7647(3)	0.48862(16)	0.0484(10)
F8_12	0.3557(5)	0.8682(6)	0.4728(4)	0.065(2)
F9_12	0.2453(2)	0.9012(3)	0.5133(2)	0.0522(10)
O1_13	0.2815(12)	0.7930(12)	0.6240(11)	0.026(3)
C1_13	0.3338(8)	0.8320(9)	0.5875(8)	0.035(2)
C2_13	0.3386(9)	0.9334(9)	0.6018(7)	0.048(3)
F1_13	0.2805(8)	0.9754(10)	0.5722(8)	0.068(4)
F2_13	0.4050(10)	0.9667(13)	0.5822(10)	0.069(4)
F3_13	0.3386(11)	0.9463(10)	0.6648(6)	0.065(4)
C3_13	0.4153(8)	0.7936(10)	0.6025(7)	0.046(3)
F4_13	0.4118(12)	0.7058(10)	0.6103(11)	0.067(5)
F5_13	0.4439(8)	0.8246(13)	0.6579(7)	0.067(4)
F6_13	0.4640(7)	0.8108(12)	0.5569(7)	0.058(4)
C4_13	0.3125(10)	0.8185(12)	0.5155(8)	0.044(3)
F7_13	0.3282(12)	0.7350(10)	0.4995(9)	0.072(4)
F8_13	0.3461(15)	0.8795(17)	0.4794(15)	0.048(5)
F9_13	0.2372(7)	0.8355(14)	0.5062(7)	0.060(4)
O1_14	0.1569(3)	0.7115(3)	0.5902(2)	0.0280(10)
C1_14	0.0984(3)	0.6610(4)	0.5656(3)	0.0305(10)
C2_14	0.0452(4)	0.7189(5)	0.5205(3)	0.0459(12)
F1_14	−0.0012(2)	0.7707(3)	0.55430(19)	0.0524(11)
F2_14	−0.0006(3)	0.6694(4)	0.4814(2)	0.0735(15)
F3_14	0.0867(3)	0.7718(4)	0.4851(2)	0.0636(13)
C3_14	0.1341(3)	0.5844(4)	0.5271(3)	0.0404(12)
F4_14	0.1941(2)	0.5484(3)	0.56031(19)	0.0474(10)
F5_14	0.1588(3)	0.6145(5)	0.4720(2)	0.0540(14)
F6_14	0.0834(3)	0.5193(3)	0.5138(2)	0.0662(14)
C4_14	0.0496(3)	0.6214(4)	0.6188(3)	0.0349(11)
F7_14	0.0859(3)	0.5543(3)	0.6492(2)	0.0451(11)
F8_14	−0.0198(2)	0.5921(3)	0.5970(2)	0.0512(11)
F9_14	0.0360(2)	0.6835(3)	0.6631(2)	0.0349(9)
O1_15	0.1280(10)	0.7262(11)	0.5937(10)	0.030(4)
C1_15	0.0974(8)	0.6490(10)	0.5702(7)	0.039(2)
C2_15	0.0352(9)	0.6690(11)	0.5164(7)	0.051(3)
F1_15	−0.0298(8)	0.6958(12)	0.5420(8)	0.067(4)
F2_15	0.0215(12)	0.5984(11)	0.4792(8)	0.078(5)
F3_15	0.0582(12)	0.7340(12)	0.4766(8)	0.064(4)
C3_15	0.1628(10)	0.5901(10)	0.5442(9)	0.050(3)
F4_15	0.2247(9)	0.5921(11)	0.5842(8)	0.061(4)
F5_15	0.1819(15)	0.6163(19)	0.4865(9)	0.064(5)
F6_15	0.1411(12)	0.5055(9)	0.5391(9)	0.067(4)
C4_15	0.0604(10)	0.5965(12)	0.6249(8)	0.045(3)
F7_15	0.1154(10)	0.5581(14)	0.6619(11)	0.049(4)
F8_15	0.0112(10)	0.5349(11)	0.6029(8)	0.058(4)
F9_15	0.0197(11)	0.6495(11)	0.6618(10)	0.049(4)
O1_16	0.1860(3)	0.7568(4)	0.7224(2)	0.0263(12)
C1_16	0.2199(4)	0.7154(4)	0.7735(3)	0.0264(11)
C2_16	0.2698(4)	0.7825(5)	0.8139(3)	0.0368(13)
F1_16	0.2233(3)	0.8375(3)	0.8452(3)	0.0579(14)

F2_16	0.3178(3)	0.7444(4)	0.8563(2)	0.0447(13)
F3_16	0.3109(5)	0.8340(5)	0.7767(4)	0.0515(16)
C3_16	0.2740(4)	0.6383(5)	0.7532(3)	0.0425(14)
F4_16	0.2417(3)	0.5932(3)	0.7046(3)	0.0622(15)
F5_16	0.3401(3)	0.6723(5)	0.7327(3)	0.0639(17)
F6_16	0.2918(3)	0.5818(3)	0.8003(3)	0.0583(15)
C4_16	0.1562(4)	0.6763(5)	0.8151(4)	0.0353(14)
F7_16	0.1302(5)	0.6002(5)	0.7886(5)	0.060(2)
F8_16	0.1817(4)	0.6552(4)	0.8735(3)	0.0396(14)
F9_16	0.0970(4)	0.7294(5)	0.8180(3)	0.0606(18)
O1_17	0.1992(10)	0.7245(10)	0.7124(6)	0.037(3)
C1_17	0.2223(8)	0.7120(9)	0.7748(7)	0.036(2)
C2_17	0.2653(10)	0.7949(10)	0.8021(7)	0.052(3)
F1_17	0.2210(9)	0.8662(9)	0.7995(9)	0.095(4)
F2_17	0.2909(10)	0.7892(12)	0.8629(6)	0.070(4)
F3_17	0.3256(12)	0.8113(16)	0.7675(11)	0.078(5)
C3_17	0.2778(9)	0.6317(9)	0.7778(7)	0.053(3)
F4_17	0.2453(9)	0.5595(8)	0.7511(7)	0.085(4)
F5_17	0.3455(9)	0.6438(13)	0.7517(10)	0.088(5)
F6_17	0.2972(8)	0.6069(11)	0.8369(6)	0.081(4)
C4_17	0.1506(10)	0.6954(13)	0.8145(8)	0.046(3)
F7_17	0.1114(13)	0.6246(14)	0.7948(13)	0.072(5)
F8_17	0.1656(12)	0.6872(13)	0.8765(8)	0.055(4)
F9_17	0.0983(11)	0.7586(14)	0.8059(12)	0.085(5)

Table S 7: Bond lengths for [DAPP^{tBu}][Al(OR^F)$_4$] **1**.

Atom–Atom	Length [Å]
P1–N1	1.644(4)
P1–C1	1.825(4)
P1–C2_6	1.834(5)
P1–C2_7	1.846(4)
P2–O1	1.476(3)
P2–C2_9	1.841(4)
P2–C2	1.849(4)
P2–C2_8	1.865(4)
Al1–O1_11	1.653(14)
Al1–O1_13	1.678(18)
Al1–O1_14	1.712(4)
Al1–O1_16	1.713(5)
Al1–O1_10	1.731(4)
Al1–O1_17	1.737(13)
Al1–O1_12	1.742(5)
Al1–O1_15	1.748(15)
N1–N1_5	1.393(5)
C1–C1_5	1.495(6)
C1–H1A	0.9900
C1–H1B	0.9900
C2–C5_5	1.495(6)
C2–H2A	0.9900
C2–H2B	0.9900
N1_5–C5_5	1.364(5)
N1_5–C1_5	1.368(5)
C1_5–C2_5	1.373(5)
C2_5–C3_5	1.387(6)
C2_5–H2_5	0.9500
C3_5–C4_5	1.379(6)
C3_5–H3_5	0.9500
C4_5–C5_5	1.390(6)
C4_5–H4_5	0.9500
C1_6–C2_6	1.529(6)
C1_6–H1A_6	0.9800
C1_6–H1B_6	0.9800
C1_6–H1C_6	0.9800
C2_6–C4_6	1.536(7)
C2_6–C3_6	1.541(6)
C3_6–H3A_6	0.9800
C3_6–H3B_6	0.9800
C3_6–H3C_6	0.9800
C4_6–H4A_6	0.9800
C4_6–H4B_6	0.9800
C4_6–H4C_6	0.9800
C1_7–C2_7	1.525(6)
C1_7–H1A_7	0.9800
C1_7–H1B_7	0.9800
C1_7–H1C_7	0.9800
C2_7–C4_7	1.530(6)
C2_7–C3_7	1.533(6)
C3_7–H3A_7	0.9800
C3_7–H3B_7	0.9800
C3_7–H3C_7	0.9800
C4_7–H4A_7	0.9800
C4_7–H4B_7	0.9800
C4_7–H4C_7	0.9800
C1_8–C2_8	1.516(6)
C1_8–H1A_8	0.9800
C1_8–H1B_8	0.9800

C1_8–H1C_8	0.9800	C4_12–F8_12	1.350(7)
C2_8–C4_8	1.526(6)	C4_12–F7_12	1.356(7)
C2_8–C3_8	1.539(6)	O1_13–C1_13	1.345(14)
C3_8–H3A_8	0.9800	C1_13–C3_13	1.542(12)
C3_8–H3B_8	0.9800	C1_13–C4_13	1.546(13)
C3_8–H3C_8	0.9800	C1_13–C2_13	1.556(12)
C4_8–H4A_8	0.9800	C2_13–F1_13	1.317(13)
C4_8–H4B_8	0.9800	C2_13–F3_13	1.332(13)
C4_8–H4C_8	0.9800	C2_13–F2_13	1.335(13)
C1_9–C2_9	1.535(6)	C3_13–F5_13	1.324(13)
C1_9–H1A_9	0.9800	C3_13–F6_13	1.328(12)
C1_9–H1B_9	0.9800	C3_13–F4_13	1.330(13)
C1_9–H1C_9	0.9800	C4_13–F7_13	1.331(13)
C2_9–C3_9	1.529(6)	C4_13–F9_13	1.333(13)
C2_9–C4_9	1.538(5)	C4_13–F8_13	1.338(13)
C3_9–H3A_9	0.9800	O1_14–C1_14	1.347(6)
C3_9–H3B_9	0.9800	C1_14–C4_14	1.549(7)
C3_9–H3C_9	0.9800	C1_14–C3_14	1.550(7)
C4_9–H4A_9	0.9800	C1_14–C2_14	1.555(7)
C4_9–H4B_9	0.9800	C2_14–F3_14	1.321(8)
C4_9–H4C_9	0.9800	C2_14–F2_14	1.337(7)
O1_10–C1_10	1.340(6)	C2_14–F1_14	1.343(7)
C1_10–C4_10	1.539(7)	C3_14–F5_14	1.327(7)
C1_10–C3_10	1.556(7)	C3_14–F6_14	1.336(6)
C1_10–C2_10	1.559(7)	C3_14–F4_14	1.337(7)
C2_10–F3_10	1.323(7)	C4_14–F7_14	1.333(7)
C2_10–F1_10	1.329(7)	C4_14–F8_14	1.339(6)
C2_10–F2_10	1.345(8)	C4_14–F9_14	1.343(6)
C3_10–F5_10	1.318(6)	O1_15–C1_15	1.357(13)
C3_10–F4_10	1.326(6)	C1_15–C2_15	1.550(12)
C3_10–F6_10	1.330(6)	C1_15–C4_15	1.554(13)
C4_10–F9_10	1.313(8)	C1_15–C3_15	1.555(13)
C4_10–F7_10	1.334(8)	C2_15–F2_15	1.330(13)
C4_10–F8_10	1.340(7)	C2_15–F1_15	1.330(13)
O1_11–C1_11	1.356(13)	C2_15–F3_15	1.356(13)
C1_11–C3_11	1.540(12)	C3_15–F5_15	1.326(13)
C1_11–C4_11	1.552(13)	C3_15–F6_15	1.327(13)
C1_11–C2_11	1.553(12)	C3_15–F4_15	1.328(13)
C2_11–F3_11	1.298(13)	C4_15–F8_15	1.325(13)
C2_11–F1_11	1.324(13)	C4_15–F7_15	1.330(13)
C2_11–F2_11	1.336(12)	C4_15–F9_15	1.333(13)
C3_11–F4_11	1.316(13)	O1_16–C1_16	1.348(7)
C3_11–F5_11	1.320(13)	C1_16–C4_16	1.551(8)
C3_11–F6_11	1.341(12)	C1_16–C2_16	1.552(8)
C4_11–F8_11	1.308(13)	C1_16–C3_16	1.562(8)
C4_11–F7_11	1.322(13)	C2_16–F2_16	1.318(7)
C4_11–F9_11	1.328(13)	C2_16–F3_16	1.327(8)
O1_12–C1_12	1.350(6)	C2_16–F1_16	1.345(8)
C1_12–C3_12	1.545(7)	C3_16–F4_16	1.323(8)
C1_12–C4_12	1.550(7)	C3_16–F6_16	1.326(7)
C1_12–C2_12	1.563(7)	C3_16–F5_16	1.341(8)
C2_12–F3_12	1.308(6)	C4_16–F9_16	1.303(8)
C2_12–F1_12	1.333(7)	C4_16–F8_16	1.319(7)
C2_12–F2_12	1.341(6)	C4_16–F7_16	1.340(8)
C3_12–F6_12	1.321(7)	O1_17–C1_17	1.360(13)
C3_12–F4_12	1.337(7)	C1_17–C3_17	1.542(12)
C3_12–F5_12	1.347(7)	C1_17–C4_17	1.545(12)
C4_12–F9_12	1.317(7)	C1_17–C2_17	1.547(12)

C2_17–F1_17	1.318(13)
C2_17–F3_17	1.319(13)
C2_17–F2_17	1.330(12)
C3_17–F6_17	1.319(12)
C3_17–F5_17	1.327(13)
C3_17–F4_17	1.333(12)
C4_17–F8_17	1.316(13)
C4_17–F9_17	1.318(13)
C4_17–F7_17	1.318(13)

Table S 8: Bond angles for [DAPP^{tBu}][Al(OR^{F})$_4$] **1**.

Atom–Atom–Atom	Angle [°]
N1–P1–C1	97.77(19)
N1–P1–C2_6	109.9(2)
C1–P1–C2_6	110.1(2)
N1–P1–C2_7	109.75(19)
C1–P1–C2_7	110.1(2)
C2_6–P1–C2_7	117.4(2)
O1–P2–C2_9	110.3(2)
O1–P2–C2	112.1(2)
C2_9–P2–C2	106.5(2)
O1–P2–C2_8	111.1(2)
C2_9–P2–C2_8	114.75(19)
C2–P2–C2_8	101.7(2)
O1_11–Al1–O1_13	113.2(9)
O1_14–Al1–O1_16	113.8(3)
O1_14–Al1–O1_10	106.6(2)
O1_16–Al1–O1_10	106.1(3)
O1_11–Al1–O1_17	116.8(9)
O1_13–Al1–O1_17	99.1(10)
O1_14–Al1–O1_12	108.8(2)
O1_16–Al1–O1_12	112.2(3)
O1_10–Al1–O1_12	109.1(2)
O1_11–Al1–O1_15	109.8(8)
O1_13–Al1–O1_15	114.6(10)
O1_17–Al1–O1_15	102.6(9)
N1_5–N1–P1	107.9(3)
C1_5–C1–P1	102.4(3)
C1_5–C1–H1A	111.3
P1–C1–H1A	111.3
C1_5–C1–H1B	111.3
P1–C1–H1B	111.3
H1A–C1–H1B	109.2
C5_5–C2–P2	116.4(3)
C5_5–C2–H2A	108.2
P2–C2–H2A	108.2
C5_5–C2–H2B	108.2
P2–C2–H2B	108.2
H2A–C2–H2B	107.3
C5_5–N1_5–C1_5	121.2(3)
C5_5–N1_5–N1	119.7(3)
C1_5–N1_5–N1	119.1(3)
N1_5–C1_5–C2_5	120.5(4)
N1_5–C1_5–C1	112.2(3)
C2_5–C1_5–C1	127.2(4)
C1_5–C2_5–C3_5	119.6(4)
C1_5–C2_5–H2_5	120.2
C3_5–C2_5–H2_5	120.2
C4_5–C3_5–C2_5	119.0(4)
C4_5–C3_5–H3_5	120.5
C2_5–C3_5–H3_5	120.5
C3_5–C4_5–C5_5	121.3(4)
C3_5–C4_5–H4_5	119.4
C5_5–C4_5–H4_5	119.4
N1_5–C5_5–C4_5	118.3(4)
N1_5–C5_5–C2	117.6(4)
C4_5–C5_5–C2	124.0(4)
C2_6–C1_6–H1A_6	109.5
C2_6–C1_6–H1B_6	109.5
H1A_6–C1_6–H1B_6	109.5
C2_6–C1_6–H1C_6	109.5
H1A_6–C1_6–H1C_6	109.5
H1B_6–C1_6–H1C_6	109.5
C1_6–C2_6–C4_6	110.3(5)
C1_6–C2_6–C3_6	109.2(4)
C4_6–C2_6–C3_6	109.8(4)
C1_6–C2_6–P1	109.9(3)
C4_6–C2_6–P1	112.2(4)
C3_6–C2_6–P1	105.4(3)
C2_6–C3_6–H3A_6	109.5
C2_6–C3_6–H3B_6	109.5
H3A_6–C3_6–H3B_6	109.5
C2_6–C3_6–H3C_6	109.5
H3A_6–C3_6–H3C_6	109.5
H3B_6–C3_6–H3C_6	109.5
C2_6–C4_6–H4A_6	109.5
C2_6–C4_6–H4B_6	109.5
H4A_6–C4_6–H4B_6	109.5
C2_6–C4_6–H4C_6	109.5
H4A_6–C4_6–H4C_6	109.5
H4B_6–C4_6–H4C_6	109.5
C2_7–C1_7–H1A_7	109.5
C2_7–C1_7–H1B_7	109.5
H1A_7–C1_7–H1B_7	109.5
C2_7–C1_7–H1C_7	109.5
H1A_7–C1_7–H1C_7	109.5
H1B_7–C1_7–H1C_7	109.5
C1_7–C2_7–C4_7	109.6(4)
C1_7–C2_7–C3_7	109.4(4)
C4_7–C2_7–C3_7	109.9(4)
C1_7–C2_7–P1	107.0(3)
C4_7–C2_7–P1	111.6(3)
C3_7–C2_7–P1	109.3(3)
C2_7–C3_7–H3A_7	109.5
C2_7–C3_7–H3B_7	109.5
H3A_7–C3_7–H3B_7	109.5
C2_7–C3_7–H3C_7	109.5
H3A_7–C3_7–H3C_7	109.5
H3B_7–C3_7–H3C_7	109.5
C2_7–C4_7–H4A_7	109.5
C2_7–C4_7–H4B_7	109.5
H4A_7–C4_7–H4B_7	109.5
C2_7–C4_7–H4C_7	109.5
H4A_7–C4_7–H4C_7	109.5

H4B_7–C4_7–H4C_7	109.5	F1_10–C2_10–F2_10	108.2(6)
C2_8–C1_8–H1A_8	109.5	F3_10–C2_10–C1_10	112.0(5)
C2_8–C1_8–H1B_8	109.5	F1_10–C2_10–C1_10	110.7(5)
H1A_8–C1_8–H1B_8	109.5	F2_10–C2_10–C1_10	111.5(5)
C2_8–C1_8–H1C_8	109.5	F5_10–C3_10–F4_10	106.8(4)
H1A_8–C1_8–H1C_8	109.5	F5_10–C3_10–F6_10	107.8(5)
H1B_8–C1_8–H1C_8	109.5	F4_10–C3_10–F6_10	105.9(5)
C1_8–C2_8–C4_8	110.7(4)	F5_10–C3_10–C1_10	111.3(4)
C1_8–C2_8–C3_8	107.3(4)	F4_10–C3_10–C1_10	111.3(4)
C4_8–C2_8–C3_8	109.4(4)	F6_10–C3_10–C1_10	113.3(4)
C1_8–C2_8–P2	110.0(3)	F9_10–C4_10–F7_10	107.2(6)
C4_8–C2_8–P2	114.0(3)	F9_10–C4_10–F8_10	106.9(5)
C3_8–C2_8–P2	105.0(3)	F7_10–C4_10–F8_10	107.8(6)
C2_8–C3_8–H3A_8	109.5	F9_10–C4_10–C1_10	110.8(6)
C2_8–C3_8–H3B_8	109.5	F7_10–C4_10–C1_10	110.7(5)
H3A_8–C3_8–H3B_8	109.5	F8_10–C4_10–C1_10	113.2(5)
C2_8–C3_8–H3C_8	109.5	C1_11–O1_11–Al1	154.1(12)
H3A_8–C3_8–H3C_8	109.5	O1_11–C1_11–C3_11	112.6(11)
H3B_8–C3_8–H3C_8	109.5	O1_11–C1_11–C4_11	106.4(12)
C2_8–C4_8–H4A_8	109.5	C3_11–C1_11–C4_11	108.9(10)
C2_8–C4_8–H4B_8	109.5	O1_11–C1_11–C2_11	111.6(11)
H4A_8–C4_8–H4B_8	109.5	C3_11–C1_11–C2_11	109.5(10)
C2_8–C4_8–H4C_8	109.5	C4_11–C1_11–C2_11	107.5(10)
H4A_8–C4_8–H4C_8	109.5	F3_11–C2_11–F1_11	98.5(13)
H4B_8–C4_8–H4C_8	109.5	F3_11–C2_11–F2_11	115.2(15)
C2_9–C1_9–H1A_9	109.5	F1_11–C2_11–F2_11	110.1(14)
C2_9–C1_9–H1B_9	109.5	F3_11–C2_11–C1_11	108.1(11)
H1A_9–C1_9–H1B_9	109.5	F1_11–C2_11–C1_11	112.2(12)
C2_9–C1_9–H1C_9	109.5	F2_11–C2_11–C1_11	112.0(12)
H1A_9–C1_9–H1C_9	109.5	F4_11–C3_11–F5_11	104.2(14)
H1B_9–C1_9–H1C_9	109.5	F4_11–C3_11–F6_11	110.7(14)
C3_9–C2_9–C1_9	109.2(4)	F5_11–C3_11–F6_11	108.5(13)
C3_9–C2_9–C4_9	109.7(4)	F4_11–C3_11–C1_11	110.5(12)
C1_9–C2_9–C4_9	107.5(4)	F5_11–C3_11–C1_11	113.5(12)
C3_9–C2_9–P2	114.5(3)	F6_11–C3_11–C1_11	109.5(12)
C1_9–C2_9–P2	110.3(3)	F8_11–C4_11–F7_11	110.2(13)
C4_9–C2_9–P2	105.3(3)	F8_11–C4_11–F9_11	109.6(14)
C2_9–C3_9–H3A_9	109.5	F7_11–C4_11–F9_11	104.3(13)
C2_9–C3_9–H3B_9	109.5	F8_11–C4_11–C1_11	113.0(12)
H3A_9–C3_9–H3B_9	109.5	F7_11–C4_11–C1_11	110.7(12)
C2_9–C3_9–H3C_9	109.5	F9_11–C4_11–C1_11	108.7(11)
H3A_9–C3_9–H3C_9	109.5	C1_12–O1_12–Al1	147.7(5)
H3B_9–C3_9–H3C_9	109.5	O1_12–C1_12–C3_12	109.7(5)
C2_9–C4_9–H4A_9	109.5	O1_12–C1_12–C4_12	111.2(5)
C2_9–C4_9–H4B_9	109.5	C3_12–C1_12–C4_12	109.8(4)
H4A_9–C4_9–H4B_9	109.5	O1_12–C1_12–C2_12	107.6(4)
C2_9–C4_9–H4C_9	109.5	C3_12–C1_12–C2_12	109.2(4)
H4A_9–C4_9–H4C_9	109.5	C4_12–C1_12–C2_12	109.3(4)
H4B_9–C4_9–H4C_9	109.5	F3_12–C2_12–F1_12	108.7(5)
C1_10–O1_10–Al1	147.9(4)	F3_12–C2_12–F2_12	107.8(4)
O1_10–C1_10–C4_10	112.9(5)	F1_12–C2_12–F2_12	107.5(5)
O1_10–C1_10–C3_10	110.1(4)	F3_12–C2_12–C1_12	111.6(4)
C4_10–C1_10–C3_10	108.6(5)	F1_12–C2_12–C1_12	109.7(4)
O1_10–C1_10–C2_10	107.5(5)	F2_12–C2_12–C1_12	111.3(4)
C4_10–C1_10–C2_10	109.4(5)	F6_12–C3_12–F4_12	108.0(5)
C3_10–C1_10–C2_10	108.2(4)	F6_12–C3_12–F5_12	107.5(5)
F3_10–C2_10–F1_10	107.6(6)	F4_12–C3_12–F5_12	108.3(6)
F3_10–C2_10–F2_10	106.7(5)	F6_12–C3_12–C1_12	113.0(5)

F4_12–C3_12–C1_12	109.7(5)
F5_12–C3_12–C1_12	110.2(5)
F9_12–C4_12–F8_12	109.9(6)
F9_12–C4_12–F7_12	108.4(5)
F8_12–C4_12–F7_12	106.3(6)
F9_12–C4_12–C1_12	111.0(5)
F8_12–C4_12–C1_12	111.4(6)
F7_12–C4_12–C1_12	109.6(5)
C1_13–O1_13–Al1	146.1(17)
O1_13–C1_13–C3_13	110.8(13)
O1_13–C1_13–C4_13	111.0(14)
C3_13–C1_13–C4_13	108.9(11)
O1_13–C1_13–C2_13	110.4(12)
C3_13–C1_13–C2_13	106.7(11)
C4_13–C1_13–C2_13	108.9(11)
F1_13–C2_13–F3_13	111.2(14)
F1_13–C2_13–F2_13	109.0(15)
F3_13–C2_13–F2_13	106.8(14)
F1_13–C2_13–C1_13	110.2(12)
F3_13–C2_13–C1_13	109.3(12)
F2_13–C2_13–C1_13	110.3(13)
F5_13–C3_13–F6_13	109.6(13)
F5_13–C3_13–F4_13	105.1(15)
F6_13–C3_13–F4_13	108.4(14)
F5_13–C3_13–C1_13	110.4(12)
F6_13–C3_13–C1_13	112.7(12)
F4_13–C3_13–C1_13	110.4(13)
F7_13–C4_13–F9_13	110.7(16)
F7_13–C4_13–F8_13	113.8(17)
F9_13–C4_13–F8_13	103.5(15)
F7_13–C4_13–C1_13	109.0(13)
F9_13–C4_13–C1_13	107.8(12)
F8_13–C4_13–C1_13	111.7(17)
C1_14–O1_14–Al1	148.8(4)
O1_14–C1_14–C4_14	111.5(5)
O1_14–C1_14–C3_14	107.8(4)
C4_14–C1_14–C3_14	109.4(5)
O1_14–C1_14–C2_14	109.3(5)
C4_14–C1_14–C2_14	108.9(5)
C3_14–C1_14–C2_14	109.8(5)
F3_14–C2_14–F2_14	108.3(5)
F3_14–C2_14–F1_14	107.6(5)
F2_14–C2_14–F1_14	107.0(5)
F3_14–C2_14–C1_14	110.7(5)
F2_14–C2_14–C1_14	112.1(5)
F1_14–C2_14–C1_14	110.9(5)
F5_14–C3_14–F6_14	107.7(5)
F5_14–C3_14–F4_14	108.4(5)
F6_14–C3_14–F4_14	107.4(5)
F5_14–C3_14–C1_14	110.6(5)
F6_14–C3_14–C1_14	112.2(5)
F4_14–C3_14–C1_14	110.4(4)
F7_14–C4_14–F8_14	108.1(5)
F7_14–C4_14–F9_14	106.9(5)
F8_14–C4_14–F9_14	106.3(5)
F7_14–C4_14–C1_14	111.8(5)
F8_14–C4_14–C1_14	113.0(5)
F9_14–C4_14–C1_14	110.4(4)

C1_15–O1_15–Al1	154.7(15)
O1_15–C1_15–C2_15	110.1(12)
O1_15–C1_15–C4_15	109.6(13)
C2_15–C1_15–C4_15	109.7(11)
O1_15–C1_15–C3_15	109.6(12)
C2_15–C1_15–C3_15	110.4(11)
C4_15–C1_15–C3_15	107.5(11)
F2_15–C2_15–F1_15	110.2(15)
F2_15–C2_15–F3_15	105.3(14)
F1_15–C2_15–F3_15	107.8(15)
F2_15–C2_15–C1_15	111.7(13)
F1_15–C2_15–C1_15	109.8(12)
F3_15–C2_15–C1_15	111.8(13)
F5_15–C3_15–F6_15	107.0(16)
F5_15–C3_15–F4_15	109.9(16)
F6_15–C3_15–F4_15	106.8(15)
F5_15–C3_15–C1_15	111.4(15)
F6_15–C3_15–C1_15	111.4(13)
F4_15–C3_15–C1_15	110.2(12)
F8_15–C4_15–F7_15	109.3(15)
F8_15–C4_15–F9_15	105.7(14)
F7_15–C4_15–F9_15	107.8(16)
F8_15–C4_15–C1_15	112.2(13)
F7_15–C4_15–C1_15	109.9(13)
F9_15–C4_15–C1_15	111.7(14)
C1_16–O1_16–Al1	149.5(5)
O1_16–C1_16–C4_16	109.0(5)
O1_16–C1_16–C2_16	110.1(5)
C4_16–C1_16–C2_16	109.4(5)
O1_16–C1_16–C3_16	111.8(5)
C4_16–C1_16–C3_16	108.8(5)
C2_16–C1_16–C3_16	107.8(5)
F2_16–C2_16–F3_16	108.0(7)
F2_16–C2_16–F1_16	108.1(6)
F3_16–C2_16–F1_16	106.1(6)
F2_16–C2_16–C1_16	113.7(6)
F3_16–C2_16–C1_16	111.1(6)
F1_16–C2_16–C1_16	109.5(6)
F4_16–C3_16–F6_16	108.6(6)
F4_16–C3_16–F5_16	106.6(6)
F6_16–C3_16–F5_16	108.1(6)
F4_16–C3_16–C1_16	110.6(5)
F6_16–C3_16–C1_16	113.2(6)
F5_16–C3_16–C1_16	109.6(6)
F9_16–C4_16–F8_16	109.5(6)
F9_16–C4_16–F7_16	106.9(6)
F8_16–C4_16–F7_16	105.7(7)
F9_16–C4_16–C1_16	112.0(6)
F8_16–C4_16–C1_16	113.3(6)
F7_16–C4_16–C1_16	109.0(7)
C1_17–O1_17–Al1	151.7(12)
O1_17–C1_17–C3_17	107.6(11)
O1_17–C1_17–C4_17	109.3(12)
C3_17–C1_17–C4_17	111.5(11)
O1_17–C1_17–C2_17	110.9(11)
C3_17–C1_17–C2_17	109.1(11)
C4_17–C1_17–C2_17	108.4(11)
F1_17–C2_17–F3_17	107.4(15)

F1_17–C2_17–F2_17	104.9(13)	F5_17–C3_17–C1_17	115.8(13)
F3_17–C2_17–F2_17	107.4(15)	F4_17–C3_17–C1_17	111.7(11)
F1_17–C2_17–C1_17	111.8(12)	F8_17–C4_17–F9_17	107.7(14)
F3_17–C2_17–C1_17	109.1(14)	F8_17–C4_17–F7_17	107.9(16)
F2_17–C2_17–C1_17	115.8(12)	F9_17–C4_17–F7_17	101.4(15)
F6_17–C3_17–F5_17	103.4(13)	F8_17–C4_17–C1_17	114.8(14)
F6_17–C3_17–F4_17	104.3(13)	F9_17–C4_17–C1_17	112.0(13)
F5_17–C3_17–F4_17	107.7(14)	F7_17–C4_17–C1_17	112.2(14)
F6_17–C3_17–C1_17	112.9(11)		

Table S 9: Atomic coordinates and U_{eq} [Å^2] for [PNOP^{tBu}][Al(OR^F)$_4$] **2**.

**Atom**	***x***	***y***	***z***	***U*eq**
P1	0.5316(6)	0.4034(3)	0.1398(4)	0.0656(14)
P2	0.5417(6)	0.2967(2)	−0.1329(5)	0.0609(13)
Al1	0.0945(4)	0.6100(2)	0.6412(3)	0.0335(9)
O1	0.509(2)	0.3888(7)	0.0020(16)	0.063(5)
N1	0.4842(19)	0.3264(8)	−0.0152(15)	0.042(4)
N2	0.511(7)	0.358(3)	−0.075(6)	0.049(9)
O2	0.482(6)	0.350(2)	0.054(5)	0.045(9)
C1	0.697(3)	0.3902(15)	0.205(2)	0.095(7)
H1A	0.727814	0.425339	0.245811	0.113
H1B	0.691025	0.359037	0.263895	0.113
C2	0.716(3)	0.2779(8)	−0.1271(18)	0.074(7)
H2A	0.724713	0.238618	−0.094858	0.089
H2B	0.750012	0.277388	−0.207787	0.089
O1_1	0.2580(12)	0.6002(9)	0.6126(14)	0.086(5)
C1_1	0.3849(12)	0.6043(5)	0.6385(10)	0.044(3)
C2_1	0.4244(19)	0.5700(7)	0.7485(13)	0.082(5)
F1_1	0.3944(18)	0.6014(6)	0.8459(8)	0.099(5)
F2_1	0.5515(18)	0.5533(11)	0.738(2)	0.153(8)
F3_1	0.360(2)	0.5181(5)	0.7409(13)	0.118(6)
C3_1	0.4575(18)	0.5796(7)	0.5302(14)	0.084(5)
F4_1	0.399(2)	0.6011(9)	0.4320(10)	0.130(6)
F5_1	0.4505(19)	0.5206(5)	0.5387(12)	0.099(5)
F6_1	0.5790(16)	0.6026(9)	0.5282(18)	0.138(7)
C4_1	0.4239(17)	0.6678(7)	0.6578(13)	0.072(4)
F7_1	0.427(2)	0.6934(6)	0.5531(12)	0.119(7)
F8_1	0.5442(14)	0.6686(7)	0.7108(19)	0.124(7)
F9_1	0.3425(14)	0.6900(5)	0.7388(13)	0.082(4)
N1_5	0.7412(18)	0.3306(7)	0.0471(12)	0.065(4)
C1_5	0.7985(16)	0.3179(8)	−0.0547(14)	0.056(4)
C2_5	0.9100(17)	0.3430(9)	−0.089(2)	0.080(6)
H2_5	0.949373	0.332125	−0.160629	0.096
C3_5	0.967(2)	0.3839(13)	−0.021(2)	0.103(9)
H3_5	1.046717	0.401478	−0.043401	0.124
C4_5	0.908(2)	0.3991(13)	0.078(2)	0.099(8)
H4_5	0.945856	0.428557	0.125197	0.119
C5_5	0.797(2)	0.3732(11)	0.1109(19)	0.084(6)
C1_6	0.632(2)	0.3863(10)	−0.260(2)	0.083(6)
H1A_6	0.632500	0.408518	−0.333106	0.125
H1B_6	0.716968	0.366801	−0.250048	0.125
H1C_6	0.617688	0.412236	−0.194049	0.125
C2_6	0.522(2)	0.3417(9)	−0.266(2)	0.079(5)
C3_6	0.530(3)	0.3049(14)	−0.375(2)	0.102(8)
H3A_6	0.616686	0.286507	−0.377523	0.153

H3B_6	0.461463	0.275271	−0.372716	0.153
H3C_6	0.517180	0.329003	−0.444090	0.153
C4_6	0.389(2)	0.3713(12)	−0.257(3)	0.095(7)
H4A_6	0.379877	0.400023	−0.319164	0.143
H4B_6	0.381995	0.390219	−0.180546	0.143
H4C_6	0.318294	0.342720	−0.265142	0.143
C1_7	0.306(2)	0.2433(19)	−0.153(3)	0.135(11)
H1A_7	0.287195	0.237915	−0.236399	0.203
H1B_7	0.290985	0.283542	−0.131658	0.203
H1C_7	0.248904	0.218458	−0.106877	0.203
C2_7	0.448(2)	0.2281(11)	−0.128(2)	0.103(7)
C3_7	0.501(4)	0.1902(13)	−0.225(3)	0.131(11)
H3A_7	0.481034	0.207816	−0.301147	0.196
H3B_7	0.596448	0.186196	−0.215411	0.196
H3C_7	0.459574	0.152241	−0.221169	0.196
C4_7	0.474(3)	0.2040(13)	−0.008(2)	0.112(8)
H4A_7	0.549980	0.177882	−0.011464	0.168
H4B_7	0.396866	0.182836	0.018182	0.168
H4C_7	0.493785	0.235384	0.046218	0.168
C1_8	0.290(3)	0.3669(11)	0.178(2)	0.101(7)
H1A_8	0.269177	0.405040	0.146298	0.151
H1B_8	0.219920	0.354907	0.231014	0.151
H1C_8	0.295926	0.339279	0.113365	0.151
C2_8	0.420(3)	0.3691(10)	0.2440(17)	0.101(7)
C3_8	0.473(4)	0.3100(11)	0.267(2)	0.119(10)
H3A_8	0.406841	0.287011	0.307186	0.179
H3B_8	0.552589	0.312809	0.316362	0.179
H3C_8	0.495284	0.291658	0.192632	0.179
C4_8	0.411(4)	0.4033(16)	0.356(3)	0.134(11)
H4A_8	0.495607	0.401255	0.398495	0.200
H4B_8	0.341648	0.387316	0.405120	0.200
H4C_8	0.390934	0.443447	0.337682	0.200
C1_9	0.615(3)	0.4942(15)	0.029(3)	0.132(11)
H1A_9	0.580649	0.480108	−0.046187	0.198
H1B_9	0.698990	0.475144	0.046929	0.198
H1C_9	0.629126	0.535808	0.024191	0.198
C2_9	0.5171(19)	0.4812(11)	0.125(2)	0.092(6)
C3_9	0.378(2)	0.4945(12)	0.084(3)	0.105(9)
H3A_9	0.356865	0.471044	0.014993	0.158
H3B_9	0.371841	0.535266	0.063334	0.158
H3C_9	0.316643	0.485754	0.146606	0.158
C4_9	0.560(4)	0.517(2)	0.230(4)	0.166(15)
H4A_9	0.588308	0.491970	0.294018	0.249
H4B_9	0.485415	0.540786	0.256110	0.249
H4C_9	0.632637	0.542465	0.208004	0.249
O1_10	0.057(4)	0.6759(12)	0.581(3)	0.063(6)
C1_10	0.008(2)	0.7117(9)	0.501(2)	0.065(4)
C2_10	0.069(3)	0.6959(10)	0.383(2)	0.076(5)
F1_10	0.198(3)	0.7118(16)	0.390(4)	0.084(7)
F2_10	0.015(3)	0.7239(12)	0.291(2)	0.091(7)
F3_10	0.067(3)	0.6389(9)	0.360(2)	0.081(6)
C3_10	−0.142(2)	0.7076(12)	0.490(3)	0.088(6)
F4_10	−0.198(3)	0.7071(18)	0.596(3)	0.111(8)
F5_10	−0.181(4)	0.6610(14)	0.429(4)	0.121(10)
F6_10	−0.195(4)	0.7536(15)	0.436(3)	0.115(9)

C4_10	0.049(3)	0.7735(9)	0.534(2)	0.081(5)
F7_10	−0.030(4)	0.7930(14)	0.619(3)	0.103(7)
F8_10	0.044(4)	0.8117(10)	0.445(2)	0.114(8)
F9_10	0.173(3)	0.7755(14)	0.579(4)	0.100(7)
O1_11	0.024(4)	0.6676(11)	0.570(3)	0.069(6)
C1_11	0.007(2)	0.7206(10)	0.5326(18)	0.069(4)
C2_11	−0.139(2)	0.7383(12)	0.542(2)	0.088(5)
F1_11	−0.172(3)	0.7431(13)	0.656(2)	0.090(7)
F2_11	−0.157(4)	0.7886(13)	0.483(3)	0.111(8)
F3_11	−0.217(3)	0.6990(14)	0.492(3)	0.109(8)
C3_11	0.048(3)	0.7257(11)	0.404(2)	0.080(5)
F4_11	0.164(3)	0.6991(13)	0.386(3)	0.084(7)
F5_11	−0.038(3)	0.6998(16)	0.331(3)	0.102(8)
F6_11	0.065(4)	0.7814(10)	0.376(3)	0.111(8)
C4_11	0.086(3)	0.7633(10)	0.611(2)	0.079(5)
F7_11	0.216(2)	0.7614(14)	0.584(3)	0.098(8)
F8_11	0.039(3)	0.8173(9)	0.607(2)	0.089(7)
F9_11	0.081(3)	0.7479(10)	0.7248(18)	0.093(7)
O1_12	0.085(4)	0.612(2)	0.795(3)	0.058(6)
C1_12	0.005(2)	0.6062(9)	0.8855(19)	0.059(4)
C2_12	0.068(3)	0.6375(12)	0.989(2)	0.078(5)
F1_12	0.168(3)	0.6058(17)	1.035(2)	0.090(7)
F2_12	−0.020(3)	0.6425(17)	1.078(2)	0.109(8)
F3_12	0.110(4)	0.6907(12)	0.962(3)	0.106(8)
C3_12	−0.132(2)	0.6301(11)	0.859(2)	0.072(5)
F4_12	−0.169(3)	0.6191(18)	0.746(2)	0.070(7)
F5_12	−0.138(3)	0.6876(10)	0.880(3)	0.082(6)
F6_12	−0.223(3)	0.6045(14)	0.928(2)	0.079(7)
C4_12	−0.002(2)	0.5412(10)	0.913(2)	0.071(5)
F7_12	−0.094(3)	0.5163(13)	0.843(2)	0.085(7)
F8_12	−0.030(4)	0.5283(14)	1.0249(19)	0.098(7)
F9_12	0.117(3)	0.5172(13)	0.889(2)	0.083(7)
O1_13	0.065(4)	0.6105(18)	0.785(2)	0.068(6)
C1_13	0.006(2)	0.6220(8)	0.8843(18)	0.061(3)
C2_13	0.077(2)	0.5931(10)	0.987(2)	0.080(5)
F1_13	0.186(3)	0.6234(14)	1.015(3)	0.106(8)
F2_13	−0.003(3)	0.5914(15)	1.0812(19)	0.109(7)
F3_13	0.109(3)	0.5385(10)	0.961(2)	0.095(6)
C3_13	−0.136(2)	0.5987(10)	0.874(2)	0.080(5)
F4_13	−0.190(4)	0.6122(18)	0.768(3)	0.099(9)
F5_13	−0.136(3)	0.5407(9)	0.885(2)	0.088(6)
F6_13	−0.208(3)	0.6219(16)	0.960(3)	0.108(8)
C4_13	0.005(2)	0.6873(9)	0.9015(18)	0.067(4)
F7_13	−0.079(2)	0.7141(12)	0.8242(19)	0.085(6)
F8_13	−0.020(3)	0.7053(11)	1.0107(16)	0.086(6)
F9_13	0.124(2)	0.7095(10)	0.874(2)	0.078(6)
O1_14	0.010(4)	0.5569(11)	0.569(3)	0.073(6)
C1_14	−0.008(2)	0.5040(9)	0.5289(17)	0.068(4)
C2_14	−0.152(2)	0.4868(12)	0.551(2)	0.092(5)
F1_14	−0.171(3)	0.4781(16)	0.667(2)	0.121(8)
F2_14	−0.185(3)	0.4376(12)	0.495(3)	0.096(8)
F3_14	−0.232(3)	0.5287(11)	0.511(3)	0.103(7)
C3_14	0.016(3)	0.4983(11)	0.396(2)	0.088(5)
F4_14	0.132(3)	0.5222(13)	0.373(2)	0.094(6)
F5_14	−0.082(3)	0.5240(16)	0.335(3)	0.118(8)

F6_14	0.023(4)	0.4428(11)	0.364(3)	0.109(8)
C4_14	0.080(3)	0.4620(10)	0.598(2)	0.086(5)
F7_14	0.201(3)	0.4607(14)	0.550(3)	0.097(7)
F8_14	0.026(4)	0.4087(10)	0.598(3)	0.123(8)
F9_14	0.092(3)	0.4774(13)	0.7117(19)	0.101(7)
O1_15	0.018(5)	0.5503(16)	0.591(3)	0.074(7)
C1_15	−0.013(2)	0.5121(10)	0.510(2)	0.076(4)
C2_15	0.103(3)	0.4714(12)	0.486(3)	0.091(5)
F1_15	0.191(4)	0.4994(15)	0.420(4)	0.109(9)
F2_15	0.063(4)	0.4243(14)	0.426(4)	0.119(10)
F3_15	0.157(4)	0.453(2)	0.588(3)	0.115(9)
C3_15	−0.132(3)	0.4757(12)	0.546(3)	0.086(6)
F4_15	−0.227(3)	0.5086(15)	0.594(3)	0.093(8)
F5_15	−0.091(4)	0.4343(14)	0.621(3)	0.110(8)
F6_15	−0.191(5)	0.4508(19)	0.451(3)	0.113(10)
C4_15	−0.048(3)	0.5440(12)	0.394(2)	0.091(6)
F7_15	−0.166(3)	0.5702(17)	0.404(3)	0.114(9)
F8_15	−0.039(5)	0.5064(16)	0.303(3)	0.116(9)
F9_15	0.046(3)	0.5828(13)	0.368(3)	0.103(8)

Table S 10: Bond lengths for $[PNOP^{tBu}][Al(OR^F)_4]$ **2**.

Atom–Atom	Length [Å]
P1–O1	1.625(19)
P1–O2	1.66(5)
P1–C2_9	1.82(3)
P1–C2_8	1.84(2)
P1–C1	1.86(3)
P2–N2	1.59(6)
P2–N1	1.627(18)
P2–C2	1.82(3)
P2–C2_6	1.85(2)
P2–C2_7	1.86(3)
Al1–O1_13	1.68(2)
Al1–O1_15	1.69(3)
Al1–O1_1	1.712(13)
Al1–O1_14	1.71(3)
Al1–O1_11	1.72(3)
Al1–O1_10	1.72(2)
Al1–O1_12	1.76(3)
O1–N1	1.49(2)
N2–O2	1.52(8)
C1–C5_5	1.54(3)
C1–H1A	0.9900
C1–H1B	0.9900
C2–C1_5	1.50(3)
C2–H2A	0.9900
C2–H2B	0.9900
O1_1–C1_1	1.323(16)
C1_1–C2_1	1.538(17)
C1_1–C4_1	1.545(17)
C1_1–C3_1	1.558(17)
C2_1–F2_1	1.356(18)
C2_1–F1_1	1.369(17)
C2_1–F3_1	1.377(17)
C3_1–F6_1	1.345(18)
C3_1–F4_1	1.361(18)
C3_1–F5_1	1.380(17)
C4_1–F7_1	1.338(16)
C4_1–F9_1	1.351(16)
C4_1–F8_1	1.358(18)
N1_5–C1_5	1.340(19)
N1_5–C5_5	1.35(2)
C1_5–C2_5	1.34(2)
C2_5–C3_5	1.35(2)
C2_5–H2_5	0.9500
C3_5–C4_5	1.33(2)
C3_5–H3_5	0.9500
C4_5–C5_5	1.34(2)
C4_5–H4_5	0.9500
C1_6–C2_6	1.53(2)
C1_6–H1A_6	0.9800
C1_6–H1B_6	0.9800
C1_6–H1C_6	0.9800
C2_6–C3_6	1.51(2)
C2_6–C4_6	1.52(2)
C3_6–H3A_6	0.9800
C3_6–H3B_6	0.9800
C3_6–H3C_6	0.9800
C4_6–H4A_6	0.9800
C4_6–H4B_6	0.9800
C4_6–H4C_6	0.9800
C1_7–C2_7	1.50(2)
C1_7–H1A_7	0.9800
C1_7–H1B_7	0.9800
C1_7–H1C_7	0.9800
C2_7–C4_7	1.50(2)
C2_7–C3_7	1.52(2)
C3_7–H3A_7	0.9800
C3_7–H3B_7	0.9800
C3_7–H3C_7	0.9800
C4_7–H4A_7	0.9800

C4_7–H4B_7	0.9800
C4_7–H4C_7	0.9800
C1_8–C2_8	1.52(2)
C1_8–H1A_8	0.9800
C1_8–H1B_8	0.9800
C1_8–H1C_8	0.9800
C2_8–C3_8	1.50(2)
C2_8–C4_8	1.51(2)
C3_8–H3A_8	0.9800
C3_8–H3B_8	0.9800
C3_8–H3C_8	0.9800
C4_8–H4A_8	0.9800
C4_8–H4B_8	0.9800
C4_8–H4C_8	0.9800
C1_9–C2_9	1.52(2)
C1_9–H1A_9	0.9800
C1_9–H1B_9	0.9800
C1_9–H1C_9	0.9800
C2_9–C3_9	1.51(2)
C2_9–C4_9	1.53(2)
C3_9–H3A_9	0.9800
C3_9–H3B_9	0.9800
C3_9–H3C_9	0.9800
C4_9–H4A_9	0.9800
C4_9–H4B_9	0.9800
C4_9–H4C_9	0.9800
O1_10–C1_10	1.33(2)
C1_10–C3_10	1.53(2)
C1_10–C2_10	1.53(2)
C1_10–C4_10	1.54(2)
C2_10–F3_10	1.35(2)
C2_10–F2_10	1.35(2)
C2_10–F1_10	1.36(2)
C3_10–F4_10	1.34(2)
C3_10–F5_10	1.35(2)
C3_10–F6_10	1.35(2)
C4_10–F7_10	1.35(2)
C4_10–F9_10	1.35(2)
C4_10–F8_10	1.35(2)
O1_11–C1_11	1.32(2)
C1_11–C3_11	1.53(2)
C1_11–C2_11	1.54(2)
C1_11–C4_11	1.55(2)
C2_11–F3_11	1.34(2)
C2_11–F1_11	1.35(2)
C2_11–F2_11	1.36(2)
C3_11–F5_11	1.35(2)
C3_11–F6_11	1.35(2)
C3_11–F4_11	1.35(2)
C4_11–F8_11	1.345(19)
C4_11–F9_11	1.36(2)
C4_11–F7_11	1.36(2)
O1_12–C1_12	1.33(2)
C1_12–C2_12	1.53(2)
C1_12–C3_12	1.53(2)
C1_12–C4_12	1.55(2)
C2_12–F3_12	1.35(2)
C2_12–F1_12	1.36(2)
C2_12–F2_12	1.37(2)
C3_12–F4_12	1.36(2)
C3_12–F6_12	1.36(2)
C3_12–F5_12	1.36(2)
C4_12–F8_12	1.348(19)
C4_12–F7_12	1.35(2)
C4_12–F9_12	1.36(2)
O1_13–C1_13	1.31(2)
C1_13–C2_13	1.53(2)
C1_13–C4_13	1.532(19)
C1_13–C3_13	1.54(2)
C2_13–F3_13	1.35(2)
C2_13–F1_13	1.35(2)
C2_13–F2_13	1.357(19)
C3_13–F6_13	1.350(19)
C3_13–F5_13	1.36(2)
C3_13–F4_13	1.36(2)
C4_13–F8_13	1.343(19)
C4_13–F9_13	1.36(2)
C4_13–F7_13	1.370(19)
O1_14–C1_14	1.33(2)
C1_14–C4_14	1.53(2)
C1_14–C2_14	1.54(2)
C1_14–C3_14	1.55(2)
C2_14–F3_14	1.35(2)
C2_14–F2_14	1.35(2)
C2_14–F1_14	1.36(2)
C3_14–F4_14	1.34(2)
C3_14–F6_14	1.34(2)
C3_14–F5_14	1.35(2)
C4_14–F7_14	1.35(2)
C4_14–F8_14	1.36(2)
C4_14–F9_14	1.36(2)
O1_15–C1_15	1.32(2)
C1_15–C3_15	1.54(2)
C1_15–C2_15	1.54(2)
C1_15–C4_15	1.55(2)
C2_15–F1_15	1.35(2)
C2_15–F2_15	1.35(2)
C2_15–F3_15	1.36(2)
C3_15–F4_15	1.35(2)
C3_15–F6_15	1.35(2)
C3_15–F5_15	1.36(2)
C4_15–F9_15	1.35(2)
C4_15–F7_15	1.35(2)
C4_15–F8_15	1.36(2)

Table S 11: Bond angles for $[PNOP^{tBu}][Al(OR^F)_4]$ **2**.

**Atom–Atom–Atom**	**Angle [°]**
O1–P1–C2_9	96.1(11)
O2–P1–C2_9	131(2)
O1–P1–C2_8	117.0(11)
O2–P1–C2_8	83(2)
C2_9–P1–C2_8	116.2(10)
O1–P1–C1	118.3(10)
O2–P1–C1	113(2)

C2_9–P1–C1	105.9(13)
C2_8–P1–C1	103.3(13)
N2–P2–C2	113(3)
N1–P2–C2	115.5(10)
N2–P2–C2_6	79(3)
N1–P2–C2_6	113.6(9)
C2–P2–C2_6	105.2(10)
N2–P2–C2_7	130(3)
N1–P2–C2_7	98.6(10)
C2–P2–C2_7	106.9(9)
C2_6–P2–C2_7	117.3(10)
O1_13–Al1–O1_15	105(2)
O1_13–Al1–O1_1	112.1(15)
O1_15–Al1–O1_1	105.4(19)
O1_1–Al1–O1_14	107.1(15)
O1_13–Al1–O1_11	112(2)
O1_15–Al1–O1_11	107(2)
O1_1–Al1–O1_11	114.6(15)
O1_1–Al1–O1_10	104.7(16)
O1_14–Al1–O1_10	109.9(17)
O1_1–Al1–O1_12	104.9(14)
O1_14–Al1–O1_12	118.2(19)
O1_10–Al1–O1_12	111(2)
N1–O1–P1	110.8(11)
O1–N1–P2	117.6(11)
O2–N2–P2	110(5)
N2–O2–P1	116(4)
C5_5–C1–P1	111.2(17)
C5_5–C1–H1A	109.4
P1–C1–H1A	109.4
C5_5–C1–H1B	109.4
P1–C1–H1B	109.4
H1A–C1–H1B	108.0
C1_5–C2–P2	114.2(13)
C1_5–C2–H2A	108.7
P2–C2–H2A	108.7
C1_5–C2–H2B	108.7
P2–C2–H2B	108.7
H2A–C2–H2B	107.6
C1_1–O1_1–Al1	153.3(12)
O1_1–C1_1–C2_1	112.9(13)
O1_1–C1_1–C4_1	110.4(13)
C2_1–C1_1–C4_1	108.4(11)
O1_1–C1_1–C3_1	105.4(12)
C2_1–C1_1–C3_1	109.7(11)
C4_1–C1_1–C3_1	110.1(10)
F2_1–C2_1–F1_1	116.6(18)
F2_1–C2_1–F3_1	101.4(17)
F1_1–C2_1–F3_1	114.1(15)
F2_1–C2_1–C1_1	108.3(14)
F1_1–C2_1–C1_1	109.2(12)
F3_1–C2_1–C1_1	106.6(13)
F6_1–C3_1–F4_1	103.6(17)
F6_1–C3_1–F5_1	116.4(17)
F4_1–C3_1–F5_1	113.7(16)
F6_1–C3_1–C1_1	108.0(13)
F4_1–C3_1–C1_1	108.2(13)
F5_1–C3_1–C1_1	106.6(12)
F7_1–C4_1–F9_1	117.4(16)
F7_1–C4_1–F8_1	111.4(18)
F9_1–C4_1–F8_1	104.2(14)
F7_1–C4_1–C1_1	107.8(13)
F9_1–C4_1–C1_1	107.9(12)
F8_1–C4_1–C1_1	107.7(13)
C1_5–N1_5–C5_5	116.5(19)
C2_5–C1_5–N1_5	122.5(16)
C2_5–C1_5–C2	125.6(16)
N1_5–C1_5–C2	111.7(17)
C1_5–C2_5–C3_5	119.6(17)
C1_5–C2_5–H2_5	120.2
C3_5–C2_5–H2_5	120.2
C4_5–C3_5–C2_5	119.1(19)
C4_5–C3_5–H3_5	120.5
C2_5–C3_5–H3_5	120.5
C3_5–C4_5–C5_5	120.3(18)
C3_5–C4_5–H4_5	119.8
C5_5–C4_5–H4_5	119.8
C4_5–C5_5–N1_5	121.9(16)
C4_5–C5_5–C1	130(2)
N1_5–C5_5–C1	107(2)
C2_6–C1_6–H1A_6	109.5
C2_6–C1_6–H1B_6	109.5
H1A_6–C1_6–H1B_6	109.5
C2_6–C1_6–H1C_6	109.5
H1A_6–C1_6–H1C_6	109.5
H1B_6–C1_6–H1C_6	109.5
C3_6–C2_6–C4_6	112(2)
C3_6–C2_6–C1_6	111.7(18)
C4_6–C2_6–C1_6	109.9(18)
C3_6–C2_6–P2	110.3(17)
C4_6–C2_6–P2	106.9(16)
C1_6–C2_6–P2	106.1(15)
C2_6–C3_6–H3A_6	109.5
C2_6–C3_6–H3B_6	109.5
H3A_6–C3_6–H3B_6	109.5
C2_6–C3_6–H3C_6	109.5
H3A_6–C3_6–H3C_6	109.5
H3B_6–C3_6–H3C_6	109.5
C2_6–C4_6–H4A_6	109.5
C2_6–C4_6–H4B_6	109.5
H4A_6–C4_6–H4B_6	109.5
C2_6–C4_6–H4C_6	109.5
H4A_6–C4_6–H4C_6	109.5
H4B_6–C4_6–H4C_6	109.5
C2_7–C1_7–H1A_7	109.5
C2_7–C1_7–H1B_7	109.5
H1A_7–C1_7–H1B_7	109.5
C2_7–C1_7–H1C_7	109.5
H1A_7–C1_7–H1C_7	109.5
H1B_7–C1_7–H1C_7	109.5
C4_7–C2_7–C1_7	115(2)
C4_7–C2_7–C3_7	113(2)
C1_7–C2_7–C3_7	110(2)
C4_7–C2_7–P2	105.2(17)
C1_7–C2_7–P2	106(2)
C3_7–C2_7–P2	106.7(17)

C2_7–C3_7–H3A_7	109.5
C2_7–C3_7–H3B_7	109.5
H3A_7–C3_7–H3B_7	109.5
C2_7–C3_7–H3C_7	109.5
H3A_7–C3_7–H3C_7	109.5
H3B_7–C3_7–H3C_7	109.5
C2_7–C4_7–H4A_7	109.5
C2_7–C4_7–H4B_7	109.5
H4A_7–C4_7–H4B_7	109.5
C2_7–C4_7–H4C_7	109.5
H4A_7–C4_7–H4C_7	109.5
H4B_7–C4_7–H4C_7	109.5
C2_8–C1_8–H1A_8	109.5
C2_8–C1_8–H1B_8	109.5
H1A_8–C1_8–H1B_8	109.5
C2_8–C1_8–H1C_8	109.5
H1A_8–C1_8–H1C_8	109.5
H1B_8–C1_8–H1C_8	109.5
C3_8–C2_8–C4_8	111.0(19)
C3_8–C2_8–C1_8	112(2)
C4_8–C2_8–C1_8	112(2)
C3_8–C2_8–P1	106.7(17)
C4_8–C2_8–P1	111(2)
C1_8–C2_8–P1	103.5(16)
C2_8–C3_8–H3A_8	109.5
C2_8–C3_8–H3B_8	109.5
H3A_8–C3_8–H3B_8	109.5
C2_8–C3_8–H3C_8	109.5
H3A_8–C3_8–H3C_8	109.5
H3B_8–C3_8–H3C_8	109.5
C2_8–C4_8–H4A_8	109.5
C2_8–C4_8–H4B_8	109.5
H4A_8–C4_8–H4B_8	109.5
C2_8–C4_8–H4C_8	109.5
H4A_8–C4_8–H4C_8	109.5
H4B_8–C4_8–H4C_8	109.5
C2_9–C1_9–H1A_9	109.5
C2_9–C1_9–H1B_9	109.5
H1A_9–C1_9–H1B_9	109.5
C2_9–C1_9–H1C_9	109.5
H1A_9–C1_9–H1C_9	109.5
H1B_9–C1_9–H1C_9	109.5
C3_9–C2_9–C1_9	111(2)
C3_9–C2_9–C4_9	113(2)
C1_9–C2_9–C4_9	106(2)
C3_9–C2_9–P1	107.9(17)
C1_9–C2_9–P1	102.3(18)
C4_9–C2_9–P1	117(3)
C2_9–C3_9–H3A_9	109.5
C2_9–C3_9–H3B_9	109.5
H3A_9–C3_9–H3B_9	109.5
C2_9–C3_9–H3C_9	109.5
H3A_9–C3_9–H3C_9	109.5
H3B_9–C3_9–H3C_9	109.5
C2_9–C4_9–H4A_9	109.5
C2_9–C4_9–H4B_9	109.5
H4A_9–C4_9–H4B_9	109.5
C2_9–C4_9–H4C_9	109.5
H4A_9–C4_9–H4C_9	109.5
H4B_9–C4_9–H4C_9	109.5
C1_10–O1_10–Al1	156(3)
O1_10–C1_10–C3_10	112(2)
O1_10–C1_10–C2_10	107(2)
C3_10–C1_10–C2_10	109.4(17)
O1_10–C1_10–C4_10	108.4(19)
C3_10–C1_10–C4_10	110.2(17)
C2_10–C1_10–C4_10	109.2(16)
F3_10–C2_10–F2_10	108(2)
F3_10–C2_10–F1_10	107(2)
F2_10–C2_10–F1_10	107(2)
F3_10–C2_10–C1_10	113.4(19)
F2_10–C2_10–C1_10	114(2)
F1_10–C2_10–C1_10	106(2)
F4_10–C3_10–F5_10	110(3)
F4_10–C3_10–F6_10	105(2)
F5_10–C3_10–F6_10	106(2)
F4_10–C3_10–C1_10	111(2)
F5_10–C3_10–C1_10	112(2)
F6_10–C3_10–C1_10	112(2)
F7_10–C4_10–F9_10	106(2)
F7_10–C4_10–F8_10	108(2)
F9_10–C4_10–F8_10	107(2)
F7_10–C4_10–C1_10	109(2)
F9_10–C4_10–C1_10	112(2)
F8_10–C4_10–C1_10	115(2)
C1_11–O1_11–Al1	160(3)
O1_11–C1_11–C3_11	111(2)
O1_11–C1_11–C2_11	110(2)
C3_11–C1_11–C2_11	108.4(17)
O1_11–C1_11–C4_11	110(2)
C3_11–C1_11–C4_11	110.9(16)
C2_11–C1_11–C4_11	106.2(16)
F3_11–C2_11–F1_11	109(2)
F3_11–C2_11–F2_11	108(2)
F1_11–C2_11–F2_11	112(2)
F3_11–C2_11–C1_11	111(2)
F1_11–C2_11–C1_11	109.7(19)
F2_11–C2_11–C1_11	108(2)
F5_11–C3_11–F6_11	111(2)
F5_11–C3_11–F4_11	105(2)
F6_11–C3_11–F4_11	106(2)
F5_11–C3_11–C1_11	113(2)
F6_11–C3_11–C1_11	110(2)
F4_11–C3_11–C1_11	111(2)
F8_11–C4_11–F9_11	105(2)
F8_11–C4_11–F7_11	112(2)
F9_11–C4_11–F7_11	105(2)
F8_11–C4_11–C1_11	114(2)
F9_11–C4_11–C1_11	111.2(19)
F7_11–C4_11–C1_11	110(2)
C1_12–O1_12–Al1	144(3)
O1_12–C1_12–C2_12	107(2)
O1_12–C1_12–C3_12	112(2)
C2_12–C1_12–C3_12	110.9(17)
O1_12–C1_12–C4_12	107(2)
C2_12–C1_12–C4_12	109.3(17)

C3_12–C1_12–C4_12	110.5(17)
F3_12–C2_12–F1_12	111(3)
F3_12–C2_12–F2_12	107(2)
F1_12–C2_12–F2_12	105(2)
F3_12–C2_12–C1_12	113(2)
F1_12–C2_12–C1_12	110(2)
F2_12–C2_12–C1_12	110(2)
F4_12–C3_12–F6_12	107(2)
F4_12–C3_12–F5_12	110(2)
F6_12–C3_12–F5_12	107(2)
F4_12–C3_12–C1_12	111(2)
F6_12–C3_12–C1_12	110(2)
F5_12–C3_12–C1_12	110.9(19)
F8_12–C4_12–F7_12	108(2)
F8_12–C4_12–F9_12	108(2)
F7_12–C4_12–F9_12	108(2)
F8_12–C4_12–C1_12	115(2)
F7_12–C4_12–C1_12	109.5(19)
F9_12–C4_12–C1_12	108.4(19)
C1_13–O1_13–Al1	159(3)
O1_13–C1_13–C2_13	111(2)
O1_13–C1_13–C4_13	108(2)
C2_13–C1_13–C4_13	109.9(16)
O1_13–C1_13–C3_13	107(2)
C2_13–C1_13–C3_13	109.8(16)
C4_13–C1_13–C3_13	110.8(16)
F3_13–C2_13–F1_13	110(2)
F3_13–C2_13–F2_13	107(2)
F1_13–C2_13–F2_13	109(2)
F3_13–C2_13–C1_13	111.0(19)
F1_13–C2_13–C1_13	109.5(19)
F2_13–C2_13–C1_13	109.7(19)
F6_13–C3_13–F5_13	109(2)
F6_13–C3_13–F4_13	110(2)
F5_13–C3_13–F4_13	108(2)
F6_13–C3_13–C1_13	109(2)
F5_13–C3_13–C1_13	110.4(19)
F4_13–C3_13–C1_13	111(2)
F8_13–C4_13–F9_13	105.7(19)
F8_13–C4_13–F7_13	110(2)
F9_13–C4_13–F7_13	103(2)
F8_13–C4_13–C1_13	115.5(19)
F9_13–C4_13–C1_13	110.1(17)
F7_13–C4_13–C1_13	111.9(18)
C1_14–O1_14–Al1	155(3)
O1_14–C1_14–C4_14	110(2)
O1_14–C1_14–C2_14	108(2)
C4_14–C1_14–C2_14	107.5(17)
O1_14–C1_14–C3_14	113(2)
C4_14–C1_14–C3_14	110.5(16)
C2_14–C1_14–C3_14	107.4(16)
F3_14–C2_14–F2_14	108(2)
F3_14–C2_14–F1_14	110(2)
F2_14–C2_14–F1_14	107(2)
F3_14–C2_14–C1_14	109.3(19)
F2_14–C2_14–C1_14	112(2)
F1_14–C2_14–C1_14	110(2)
F4_14–C3_14–F6_14	107(2)
F4_14–C3_14–F5_14	111(2)
F6_14–C3_14–F5_14	109(2)
F4_14–C3_14–C1_14	108.0(18)
F6_14–C3_14–C1_14	110.7(19)
F5_14–C3_14–C1_14	110(2)
F7_14–C4_14–F8_14	110(2)
F7_14–C4_14–F9_14	109(2)
F8_14–C4_14–F9_14	105(2)
F7_14–C4_14–C1_14	109.8(19)
F8_14–C4_14–C1_14	111(2)
F9_14–C4_14–C1_14	112(2)
C1_15–O1_15–Al1	154(4)
O1_15–C1_15–C3_15	112(2)
O1_15–C1_15–C2_15	111(2)
C3_15–C1_15–C2_15	108.4(18)
O1_15–C1_15–C4_15	109(2)
C3_15–C1_15–C4_15	108.8(18)
C2_15–C1_15–C4_15	108.2(18)
F1_15–C2_15–F2_15	108(3)
F1_15–C2_15–F3_15	112(3)
F2_15–C2_15–F3_15	107(3)
F1_15–C2_15–C1_15	109(2)
F2_15–C2_15–C1_15	111(2)
F3_15–C2_15–C1_15	110(2)
F4_15–C3_15–F6_15	105(3)
F4_15–C3_15–F5_15	111(3)
F6_15–C3_15–F5_15	109(3)
F4_15–C3_15–C1_15	111(2)
F6_15–C3_15–C1_15	111(2)
F5_15–C3_15–C1_15	109(2)
F9_15–C4_15–F7_15	110(3)
F9_15–C4_15–F8_15	102(2)
F7_15–C4_15–F8_15	115(3)
F9_15–C4_15–C1_15	111(2)
F7_15–C4_15–C1_15	110(2)
F8_15–C4_15–C1_15	109(2)

6. Computational details

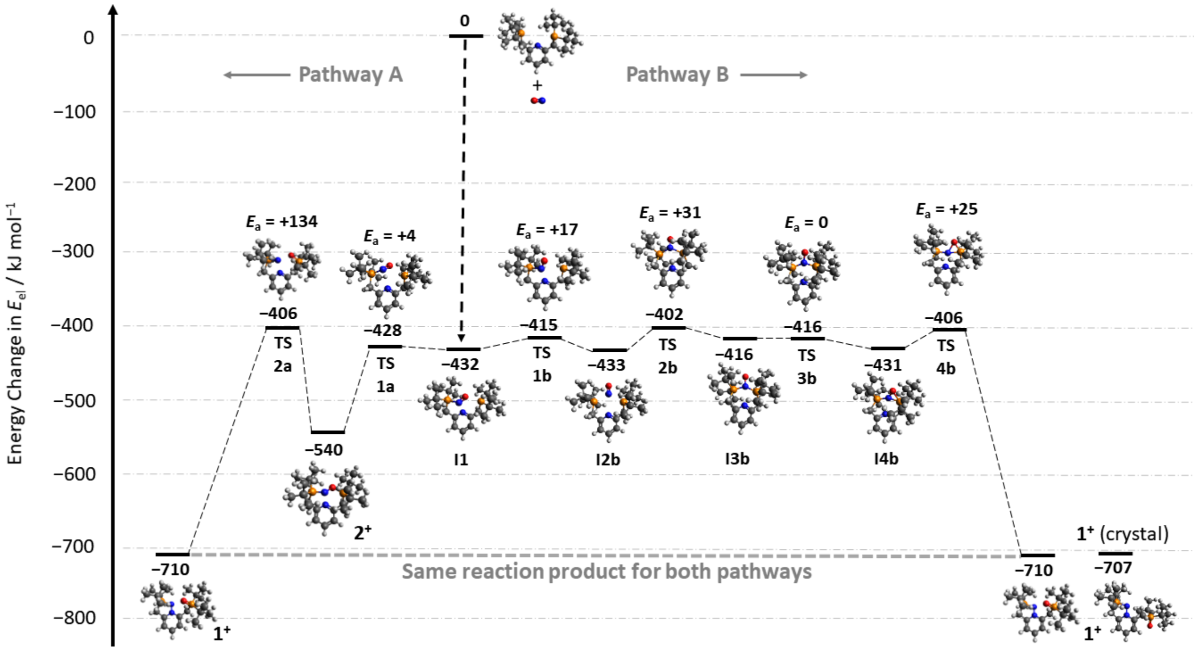

Figure S 36: Plot of the mechanistic analysis of the formation of $[DAPP^{tBu}]^+$ in the gas phase in units of E_{el} (electronic energy) at the RI-r2SCAN-3c(D4)/def2-mTZVPP level of theory.

Table S 12: Summary of computed thermodynamic data for molecular compounds in the gas phase at the RI-r2SCAN-3c(D4)/def2-mTZVPP level of theory. E_{el} = electronic energy, $H°$ = standard enthalpy, $G°$ = standard Gibbs energy. Intermediates in the mechanism are abbreviated as Inx (n = running number, x = a or b indicating the two possible mechanisms).

Molecule	E_{el} / kJ mol^{-1}	$H°$ / kJ mol^{-1}	$G°$ / kJ mol^{-1}
NO^+	-340063.2843	-340039.8764	-340098.9239
PNP^{tBu}	-4304438.277	-4302739.620	-4302977.565
I1	-4644933.599	-4643205.828	-4643454.876
I2a ($[PNOP]^+$ **2**$^+$)	-4645041.971	-4643309.481	-4643546.197
I2b	-4644934.989	-4643207.185	-4643456.291
I3b	-4644917.995	-4643184.923	-4643422.367
I4b	-4644932.928	-4643199.740	-4643433.285
$[DAPP]^+$ **1**$^+$	-4645211.723	-4643476.600	-4643715.005
$[DAPP]^+$ **1**$^+$ (crystal)	-4645208.407	-4643473.703	-4643713.450

Table S 13: Summary of computed thermodynamic data for transition states in the gas phase at the RI-r2SCAN-3c(D4)/def2-mTZVPP level of theory. E_{el} = electronic energy, $H°$ = standard enthalpy, $G°$ = standard Gibbs energy. Transition states are abbreviated as TSnx (n = running number, x = a or b indicating the two possible mechanisms).

Transition State	E_{el} / kJ mol^{-1}	$H°$ / kJ mol^{-1}	$G°$ / kJ mol^{-1}
TS1a	-4644929.219	-4643204.39	-4643446.853
TS2a	-4644908.089	-4643182.503	-4643422.398
TS1b	-4644916.459	-4643191.161	-4643437.273
TS2b	-4644903.553	-4643175.713	-4643410.466
TS3b	-4644917.914	-4643187.298	-4643417.946
TS4b	-4644908.051	-4643179.909	-4643414.077

Coordinates of computed structures at the RI-r2SCAN-3c(D4)/def2-mTZVPP level of theory in Angstrom

NO$^+$

N	2.271205	-0.137316	0.839674
O	1.211005	-0.094494	0.845626

PNP^{tBu}

C	15.040959	11.136965	3.913146
N	13.267121	10.078843	5.163323
C	14.342896	10.873304	5.216402
C	14.770307	11.462562	6.408145
H	15.644693	12.105313	6.421110
C	14.052172	11.209735	7.570477
H	14.361083	11.649563	8.513972
C	12.928286	10.398457	7.508775
H	12.335600	10.196550	8.395459
C	12.559997	9.855152	6.273622
P	13.868364	11.983815	2.688094
H	15.390987	10.192607	3.484205
C	13.769132	10.720959	1.254847
C	14.945018	13.485690	2.177124
C	13.300792	9.392594	1.873437
C	15.054019	10.482602	0.459792
C	12.651046	11.197931	0.311693
H	15.900392	10.237121	1.110064
H	15.325214	11.350470	-0.147385
H	14.908128	9.636890	-0.225590
H	11.710339	11.321315	0.857560
H	12.493832	10.446133	-0.472202
H	12.887522	12.144203	-0.180302
H	14.079051	8.910272	2.471133
H	13.033953	8.705519	1.059919
H	12.427531	9.527415	2.518873
C	14.847155	14.428361	3.391685
C	14.288690	14.183163	0.978071
C	16.426728	13.246556	1.862694
H	13.211890	14.316915	1.128336
H	14.442162	13.624764	0.050346
H	14.736770	15.175663	0.840948
H	16.965698	12.787666	2.696333
H	16.910479	14.211957	1.662175
H	16.569257	12.618939	0.982533
H	13.806906	14.689242	3.609140
H	15.401670	15.352638	3.184956
H	15.275738	13.981082	4.295448
C	11.339234	8.985947	6.139089
P	10.245331	9.403270	4.660607
H	10.778254	8.972183	7.080315
H	11.676864	7.958484	5.953179
C	9.212359	10.872089	5.311245
C	9.183748	7.807739	4.634574
C	10.235526	11.958252	5.690882
C	8.296859	10.609347	6.507949
C	8.388953	11.416967	4.131762
H	9.033760	11.630308	3.272974
H	7.902873	12.353657	4.432805
H	7.606499	10.727620	3.808065
H	8.842811	10.182126	7.355512
H	7.470627	9.939068	6.256339
H	7.857285	11.556888	6.847604
H	10.760579	11.722115	6.619707
H	9.706962	12.908244	5.840544
H	10.985260	12.106704	4.904663
C	10.049018	6.791194	3.865056
C	7.910749	8.050450	3.813886
C	8.804492	7.204621	5.992334
H	8.133664	8.541500	2.860440
H	7.182683	8.659041	4.356788
H	7.433010	7.087784	3.591372
H	9.684487	6.897190	6.564050
H	8.193387	6.306495	5.831530
H	8.222260	7.894349	6.606848
H	10.271107	7.146831	2.854306
H	9.511370	5.837460	3.787222
H	11.002530	6.590396	4.365045
H	15.919330	11.762228	4.084924

I1

P	9.744960	6.854604	7.464126
P	9.773188	10.022581	3.854184
O	10.657284	9.341441	6.423966
N	9.835003	9.995477	5.903689
C	8.010149	6.400930	6.924532
H	8.125359	5.613992	6.167722
H	7.434121	5.961326	7.745937
C	7.956244	9.899783	3.569357
H	7.551216	10.904309	3.423058
H	7.799967	9.364100	2.623019
N	7.883012	8.248389	5.350399
C	7.244308	7.522084	6.281546
C	5.901774	7.753673	6.586529
H	5.404094	7.155846	7.342344
C	5.217381	8.753717	5.910643
H	4.176572	8.957473	6.139458
C	5.876106	9.485139	4.930332
H	5.364300	10.263084	4.374261
C	7.212854	9.188611	4.678752
C	9.939205	7.369558	3.059757
H	8.953453	7.393010	2.586642
H	9.812519	7.102156	4.109898
H	10.524050	6.586227	2.564646
C	10.704519	8.690643	2.898170
C	10.822927	9.041784	1.409973
H	11.279303	8.187451	0.896210
H	11.464919	9.907962	1.236892
H	9.848517	9.221161	0.944456
C	12.098024	8.532350	3.533789
H	12.620271	7.726182	3.006670
H	12.032155	8.255152	4.589116
H	12.709908	9.431331	3.443563
C	9.558183	12.677729	4.551887

H	9.733753	13.727363	4.293416
H	9.981117	12.500671	5.542970
H	8.475279	12.533661	4.611030
C	10.227045	11.808834	3.467223
C	11.747929	11.995418	3.554246
H	12.274832	11.503231	2.733699
H	12.154879	11.636127	4.504866
H	11.968191	13.066169	3.486444
C	9.717507	12.245798	2.085282
H	10.182581	11.691887	1.269051
H	9.969062	13.304151	1.952074
H	8.631599	12.159576	1.991054
C	10.934268	4.912560	5.930079
H	11.570670	5.703559	5.521046
H	10.033254	4.840448	5.311227
H	11.469676	3.960803	5.833965
C	10.611449	5.158759	7.414771
C	11.934865	5.244301	8.185560
H	12.521133	4.339488	7.988180
H	11.780229	5.301632	9.265538
H	12.534925	6.105387	7.872654
C	9.774510	3.986162	7.941630
H	8.845801	3.852441	7.380205
H	9.526596	4.089740	8.998777
H	10.354520	3.061992	7.827742
C	9.058302	6.433848	10.258268
H	8.158131	5.896930	9.943563
H	8.824832	6.931726	11.207657
H	9.845653	5.704329	10.461120
C	9.495849	7.490024	9.239838
C	10.803196	8.152825	9.703137
H	11.620756	7.437605	9.810786
H	10.638661	8.613378	10.684263
H	11.120525	8.937565	9.011118
C	8.412113	8.578623	9.151370
H	8.357153	9.098224	10.114879
H	7.422696	8.160238	8.949153
H	8.636400	9.326198	8.383463

I2a ([PNOP]$^+$ 2$^+$)

P	9.584833	6.937864	7.172434
P	9.709338	9.537752	4.123973
O	10.216187	7.658240	5.889922
N	9.947642	9.139911	5.708353
C	7.818963	6.377885	6.980757
H	7.851588	5.438831	6.417504
H	7.425463	6.156772	7.979012
C	7.960859	9.350819	3.385848
H	7.619504	10.300383	2.971158
H	8.021148	8.627867	2.567897
N	7.548482	7.787526	5.103588
C	6.984455	7.386095	6.237372
C	5.790484	7.924487	6.710829
H	5.334372	7.565202	7.627269
C	5.211827	8.957941	5.980396
H	4.274914	9.397267	6.306432
C	5.867736	9.460155	4.860567
H	5.470767	10.310766	4.316786
C	7.051896	8.843366	4.458443
C	10.235520	7.215920	2.682802
H	9.408327	7.242508	1.969167
H	9.883170	6.741136	3.600186
H	11.016119	6.585477	2.242727
C	10.836896	8.614785	2.929556
C	10.961272	9.344698	1.586129
H	11.500411	8.693899	0.888256
H	11.529255	10.274302	1.672850
H	9.986782	9.564258	1.137254
C	12.223521	8.463169	3.574455
H	12.871516	7.927050	2.872074
H	12.174611	7.893620	4.504316
H	12.690533	9.425250	3.789171
C	9.100258	11.882099	5.443554
H	9.145681	12.976811	5.472673
H	9.466885	11.492874	6.394424
H	8.049126	11.594771	5.331581
C	9.965397	11.399609	4.262725
C	11.440455	11.715337	4.550309
H	12.073325	11.538158	3.676606
H	11.820151	11.133884	5.393276
H	11.523139	12.778776	4.800575
C	9.524555	12.154521	2.997744
H	10.002834	11.794032	2.086041
H	9.814153	13.203252	3.124392
H	8.441773	12.143748	2.855034
C	10.637926	4.821432	5.769656
H	11.209318	5.471304	5.105311
H	9.642366	4.673301	5.340330
H	11.130353	3.843749	5.801311
C	10.600756	5.372440	7.209139
C	12.038931	5.668158	7.659826
H	12.634178	4.761091	7.511563
H	12.099409	5.927631	8.719089
H	12.494522	6.466916	7.068518
C	9.958503	4.324254	8.132961
H	8.970375	4.009080	7.787799
H	9.879516	4.656229	9.169463
H	10.599650	3.436695	8.123250
C	9.561888	7.228002	9.958650
H	8.653311	6.618193	9.994016
H	9.507238	7.933155	10.794850
H	10.428517	6.586258	10.130730
C	9.681976	8.051745	8.665980
C	11.013205	8.821953	8.653119
H	11.879107	8.164189	8.744290
H	11.015611	9.490975	9.520470
H	11.113324	9.423611	7.747787
C	8.506546	9.046352	8.604083
H	8.649334	9.777457	9.407395
H	7.546298	8.554653	8.777262
H	8.471337	9.578415	7.651164

I2b

P	0.035262	-1.272605	1.654893
P	0.230523	1.377937	-1.591211
O	2.356338	0.410520	0.432253
N	1.239594	0.666124	0.584840
C	-1.564868	-2.065744	1.109044
H	-1.292714	-2.897708	0.446861
H	-2.090829	-2.507233	1.963302
C	-1.587492	1.789455	-1.685769
H	-1.733737	2.741819	-1.161856
H	-1.878889	1.966789	-2.728752
N	-1.797095	-0.134518	-0.279816
C	-2.446644	-1.115362	0.341763
C	-3.827608	-1.261548	0.233024
H	-4.347279	-2.064469	0.745321
C	-4.520935	-0.352975	-0.561807
H	-5.597248	-0.437578	-0.670326
C	-3.831942	0.659729	-1.221466
H	-4.355256	1.374492	-1.847971
C	-2.450724	0.731528	-1.048010
C	-0.423271	-0.895917	-3.054835
H	-1.447705	-0.555410	-3.231583
H	-0.397964	-1.436798	-2.105250
H	-0.163492	-1.595493	-3.857247
C	0.594715	0.254994	-3.078118
C	0.503793	0.977541	-4.426788
H	0.640961	0.237404	-5.224539
H	1.282308	1.733331	-4.548331
H	-0.472039	1.446688	-4.584095
C	1.997626	-0.344421	-2.889542
H	2.201350	-1.029589	-3.720212
H	2.061276	-0.920399	-1.962294
H	2.786356	0.409773	-2.883434
C	0.874793	3.737902	-0.329944
H	1.241795	4.769196	-0.378052
H	1.458230	3.214613	0.431596
H	-0.166466	3.780305	0.005520
C	1.030749	3.096989	-1.723704
C	2.527970	2.977888	-2.040048
H	2.708279	2.639551	-3.062963
H	3.048609	2.306790	-1.351032
H	2.984824	3.969103	-1.941095
C	0.357349	4.008359	-2.761563
H	0.398889	3.602204	-3.773021
H	0.888412	4.967806	-2.769673
H	-0.685648	4.221948	-2.514897
C	1.670731	-3.063966	0.400292
H	2.248063	-2.236269	-0.021309
H	0.838403	-3.286891	-0.275572
H	2.319697	-3.946926	0.419510
C	1.204767	-2.754257	1.835606
C	2.430537	-2.373155	2.677414
H	3.165014	-3.183689	2.609468
H	2.180981	-2.249863	3.733800
H	2.914431	-1.459777	2.320180
C	0.537167	-4.000719	2.433276
H	-0.280750	-4.376027	1.813087
H	0.157873	-3.830428	3.442338
H	1.288115	-4.797939	2.493305
C	-0.744170	-1.416508	4.411774
H	-1.540245	-2.109411	4.123912
H	-1.104193	-0.850739	5.279491
H	0.123351	-1.995105	4.737244
C	-0.393294	-0.425600	3.295522
C	0.777029	0.466518	3.741959
H	1.697733	-0.092135	3.916241
H	0.502212	0.949200	4.686569
H	0.979719	1.258326	3.016302
C	-1.606539	0.480358	3.019723
H	-1.799481	1.079398	3.916718
H	-2.513328	-0.091898	2.804869
H	-1.424074	1.165126	2.185999

I3b

P	12.235866	10.958185	2.954057
P	10.022192	10.365547	5.251014
C	13.834643	10.064004	3.470129
H	13.785386	9.026582	3.119555
H	14.683855	10.533553	2.963527
C	10.938788	10.903080	6.815851
H	10.825519	11.991789	6.894942
H	10.448162	10.464429	7.688723
N	12.705347	10.508279	5.402254
C	13.887266	10.110681	4.960898
C	14.890798	9.750488	5.854290
H	15.864031	9.414138	5.515467
C	14.590311	9.828568	7.215662
H	15.349369	9.565318	7.944911
C	13.320942	10.213401	7.655626
H	13.081699	10.242521	8.712779
C	12.380367	10.538009	6.684842
C	8.019834	11.470520	6.918818
H	7.925045	10.512262	7.432786
H	7.035834	11.949054	6.951671
H	8.700666	12.111117	7.483129
C	8.401988	11.346442	5.427111
C	8.650494	12.756875	4.875456
H	9.525103	13.240702	5.324981
H	7.779201	13.372987	5.122432
H	8.762807	12.716178	3.791519
C	7.220792	10.716151	4.675266
H	7.466638	10.533925	3.629795
H	6.386728	11.425006	4.722177
H	6.879512	9.791344	5.146795
C	8.982426	8.143289	6.664076
H	8.937574	7.051005	6.734610
H	7.953633	8.504015	6.617417
H	9.447488	8.503556	7.586287
C	9.786167	8.493396	5.401860
C	9.065882	7.930188	4.167758
H	9.008573	6.842120	4.287857
H	9.613283	8.150730	3.254629
H	8.053012	8.311696	4.054898
C	11.165912	7.819432	5.490171
H	11.715891	8.082389	6.397303
H	11.783522	8.052603	4.618689

H	11.006557	6.736084	5.503596
C	13.392637	10.066895	0.524151
H	13.195194	9.769958	-0.511136
H	13.971978	9.259423	0.976559
H	14.008652	10.965478	0.486098
C	12.014402	10.267512	1.193657
C	11.356930	8.883014	1.322180
H	11.416068	8.391759	0.344979
H	10.310866	8.994060	1.604901
H	11.869739	8.232470	2.039915
C	11.152323	11.149740	0.278241
H	11.672021	12.063669	-0.019559
H	10.197091	11.394888	0.742329
H	10.958127	10.580350	-0.637634
C	13.131208	13.253090	4.342174
H	14.054277	12.760970	4.659849
H	12.362113	13.086127	5.099866
H	13.327754	14.329561	4.297371
C	12.667271	12.807358	2.946036
C	13.787757	13.123654	1.942444
H	13.482078	12.960819	0.908312
H	14.711166	12.570299	2.135607
H	14.027728	14.187705	2.042496
C	11.414992	13.615868	2.578045
H	11.676497	14.678668	2.626834
H	10.605498	13.431896	3.280941
H	11.051290	13.404830	1.573702
N	10.695434	10.749074	3.726716
O	9.622733	10.740049	2.851014

I4b

P	12.424051	11.035918	3.026858
P	9.859166	10.359971	5.045558
C	14.009471	10.155481	3.589403
H	14.070236	9.149391	3.163316
H	14.867305	10.724864	3.209734
C	10.783570	11.076383	6.597340
H	10.746505	12.163080	6.451334
H	10.258224	10.838015	7.521899
N	12.789945	10.773206	5.428980
C	13.886540	10.110345	5.082617
C	14.606136	9.376030	6.018007
H	15.509622	8.840114	5.750551
C	14.094651	9.330986	7.316905
H	14.633092	8.787362	8.086251
C	12.859952	9.905388	7.615091
H	12.413713	9.784344	8.596318
C	12.195948	10.609769	6.608291
C	7.831608	11.590363	6.673437
H	7.723158	10.665967	7.246319
H	6.850925	12.078109	6.664396
H	8.514575	12.257058	7.203235
C	8.242858	11.347295	5.212431
C	8.488093	12.706870	4.539777
H	9.272557	13.281383	5.041061
H	7.565020	13.292125	4.613521
H	8.739325	12.593745	3.483139
C	7.086117	10.641067	4.489965
H	7.361563	10.300468	3.490532
H	6.263629	11.357178	4.386700
H	6.704014	9.797643	5.068032
C	9.499333	8.238094	6.891875
H	9.432445	7.154469	7.036039
H	8.543783	8.662878	7.209814
H	10.289682	8.602855	7.551331
C	9.792914	8.498797	5.406597
C	8.723421	7.786648	4.562790
H	8.842398	6.708111	4.713167
H	8.831776	7.996988	3.498036
H	7.710389	8.043681	4.871178
C	11.160425	7.889920	5.052618
H	11.965617	8.253296	5.692754
H	11.420433	8.068918	4.008289
H	11.093301	6.805882	5.196132
C	13.426473	10.303945	0.451759
H	13.193982	9.762564	-0.471631
H	14.242277	9.761198	0.936843
H	13.784017	11.289320	0.162648
C	12.134395	10.326653	1.291703
C	11.693735	8.856448	1.456530
H	11.538294	8.448516	0.452052
H	10.759691	8.764791	2.008452
H	12.467045	8.243490	1.929739
C	11.046172	11.109142	0.541429
H	11.379440	12.114059	0.271986
H	10.118056	11.174197	1.113268
H	10.828507	10.579834	-0.392773
C	13.548562	13.333202	4.303238
H	14.486883	12.802092	4.486098
H	12.907253	13.217776	5.176992
H	13.789389	14.396538	4.198609
C	12.851303	12.893364	3.006733
C	13.793874	13.209908	1.833787
H	13.296231	13.125486	0.867160
H	14.699373	12.596144	1.826479
H	14.114760	14.250976	1.940750
C	11.561472	13.707967	2.855617
H	11.837860	14.760630	2.732952
H	10.939892	13.621837	3.745223
H	10.963566	13.416092	1.990281
N	10.926930	10.842700	3.803943
O	9.703406	10.211247	3.234447

$[DAPP]^+$ 1^+

P	13.141242	11.670518	2.857815
P	9.431803	9.666624	5.170080
C	14.681413	10.889862	3.518660
H	15.152344	10.206822	2.808052
H	15.424552	11.638408	3.812610
C	10.765548	10.394356	6.309409
H	10.700508	11.478076	6.170380
H	10.498912	10.165575	7.345525
N	12.831524	10.498245	4.989970
C	14.148559	10.169924	4.725564
C	14.834846	9.309558	5.547189
H	15.866101	9.069258	5.315944

C	14.198711	8.781123	6.668506
H	14.727996	8.116123	7.340179
C	12.882478	9.128797	6.914663
H	12.374830	8.749924	7.792635
C	12.168476	9.970425	6.066399
C	7.869578	11.630654	6.556799
H	7.519995	10.891344	7.279333
H	7.083516	12.388063	6.459258
H	8.746620	12.134092	6.973391
C	8.138832	11.029642	5.169652
C	8.686722	12.123938	4.237539
H	9.652808	12.512924	4.570746
H	7.972684	12.955047	4.218126
H	8.809325	11.745500	3.220345
C	6.828794	10.496361	4.568337
H	6.999703	9.982592	3.617735
H	6.162058	11.344473	4.376015
H	6.306139	9.820206	5.249279
C	8.067070	8.255609	7.282516
H	7.877971	7.268531	7.720063
H	7.095803	8.727977	7.118849
H	8.618268	8.838478	8.028319
C	8.849659	8.072856	5.979158
C	7.997997	7.315323	4.944446
H	7.768130	6.322886	5.347849
H	8.541426	7.191931	4.004763
H	7.050114	7.812422	4.733772
C	10.109207	7.233953	6.256065
H	10.665517	7.612282	7.115949
H	10.773178	7.192045	5.386804
H	9.803735	6.209721	6.496000
C	13.608376	10.731236	0.233546
H	13.338690	9.963060	-0.499053
H	14.636604	10.522348	0.548760
H	13.589810	11.693331	-0.278535
C	12.601045	10.646326	1.389602
C	12.525748	9.195962	1.904261
H	12.136359	8.578798	1.087273
H	11.831560	9.099654	2.743550
H	13.509167	8.793430	2.166912
C	11.201725	11.092270	0.946327
H	11.200867	12.101940	0.526363
H	10.491497	11.025553	1.775542
H	10.858042	10.410403	0.161182
C	14.166183	14.069306	3.733469
H	15.218568	13.832629	3.552270
H	13.877890	13.708114	4.725716
H	14.075300	15.160378	3.738104
C	13.249225	13.519405	2.628761
C	13.796271	13.912946	1.252809
H	13.098727	13.665651	0.449833
H	14.765662	13.452529	1.037847
H	13.938287	14.998720	1.241003
C	11.831967	14.090483	2.827212
H	11.880875	15.171187	2.657215
H	11.476722	13.914999	3.844062
H	11.103204	13.673738	2.128479
N	12.207987	11.423787	4.207178
O	9.942501	9.366139	3.786449

[DAPP]$^+$ 1$^+$ (crystal)

P	12.968690	11.263669	2.698270
P	9.863224	10.065190	7.536685
O	10.672788	10.210975	8.796388
N	12.037237	10.682331	3.935657
C	14.505624	11.560885	3.678850
H	15.381189	11.076602	3.237811
H	14.730860	12.626735	3.780081
C	10.928948	9.385253	6.134087
H	10.958058	8.305728	6.323116
H	10.460184	9.548036	5.163351
N	12.839970	10.493704	5.035887
C	14.140858	10.950569	5.003137
C	14.956139	10.820665	6.102221
H	15.973871	11.190009	6.049169
C	14.459944	10.214165	7.254316
H	15.086991	10.104886	8.131081
C	13.156245	9.752308	7.264148
H	12.722859	9.300792	8.148166
C	12.325943	9.890319	6.152890
C	7.993200	8.245745	6.358827
H	7.532358	9.037008	5.763896
H	7.221217	7.493115	6.555576
H	8.765022	7.761251	5.753594
C	8.535102	8.750229	7.702989
C	9.199543	7.587371	8.461705
H	10.023216	7.133840	7.900239
H	8.449373	6.805322	8.623146
H	9.583427	7.913179	9.430735
C	7.385789	9.294783	8.565288
H	7.753813	9.730013	9.499099
H	6.716714	8.466284	8.822830
H	6.790193	10.042173	8.035071
C	8.240165	11.680898	5.805465
H	8.025831	12.703541	5.472493
H	7.288750	11.232763	6.102074
H	8.632380	11.130827	4.942911
C	9.241373	11.739227	6.962283
C	8.630546	12.458119	8.176953
H	8.409433	13.493350	7.893381
H	9.329430	12.469702	9.017086
H	7.697200	12.000117	8.508102
C	10.485908	12.525149	6.514237
H	10.882458	12.135190	5.571779
H	11.268732	12.513879	7.279573
H	10.196125	13.568946	6.344469
C	14.466868	10.332544	0.486040
H	14.701867	9.471270	-0.148573
H	15.387875	10.600723	1.013126
H	14.190054	11.157164	-0.171957
C	13.335267	9.927367	1.440172
C	13.763396	8.699898	2.265592
H	13.978699	7.881446	1.570965
H	12.968311	8.373462	2.940952
H	14.673466	8.876901	2.847745
C	12.059079	9.585917	0.657610

H	11.768590	10.384628	-0.029256
H	11.220197	9.364247	1.324707
H	12.251134	8.691420	0.055764
C	12.379353	13.904515	3.149724
H	13.411299	14.263800	3.190081
H	12.078987	13.553960	4.141770
H	11.748094	14.763056	2.898707
C	12.176461	12.831432	2.066494
C	12.785950	13.306995	0.743560
H	12.553452	12.633966	-0.084516
H	13.871538	13.435313	0.805294
H	12.354241	14.283928	0.501824
C	10.668853	12.561939	1.907538
H	10.193728	13.494644	1.585859
H	10.217957	12.254724	2.854284
H	10.453744	11.803802	1.151961

TS1a

P	9.683566	6.928559	7.418145
P	9.852871	9.989864	4.076054
O	10.937164	8.757176	6.196675
N	10.322924	9.762145	5.881065
C	8.007532	6.291138	6.915747
H	8.183088	5.440359	6.243890
H	7.444524	5.893989	7.768741
C	8.019141	10.171642	3.997777
H	7.790541	11.162749	4.407479
H	7.722000	10.208300	2.941707
N	7.884950	8.266199	5.509887
C	7.201915	7.312885	6.163073
C	5.811334	7.229464	6.097876
H	5.283774	6.455289	6.644397
C	5.120679	8.142035	5.312367
H	4.038857	8.099005	5.242435
C	5.831708	9.103474	4.607066
H	5.322215	9.823360	3.975291
C	7.219282	9.128542	4.738314
C	9.611291	7.414984	3.083529
H	8.550804	7.564699	2.862152
H	9.705197	7.054978	4.110134
H	9.989419	6.642192	2.405005
C	10.444681	8.685859	2.851176
C	10.269736	9.151481	1.399301
H	10.532435	8.313391	0.743260
H	10.930745	9.982685	1.145226
H	9.237916	9.433104	1.167736
C	11.924058	8.361763	3.128985
H	12.211756	7.533375	2.472145
H	12.086381	8.045890	4.161343
H	12.587151	9.198460	2.909191
C	10.181372	12.570826	5.050231
H	10.442701	13.611343	4.829560
H	10.773094	12.238726	5.903746
H	9.127032	12.555900	5.340935
C	10.475796	11.744398	3.781105
C	11.992814	11.736514	3.546261
H	12.258857	11.303122	2.579678
H	12.530005	11.206048	4.338475
H	12.343707	12.773824	3.546333
C	9.762449	12.397421	2.586369
H	9.895534	11.845744	1.654330
H	10.193128	13.394431	2.441814
H	8.692591	12.533827	2.764236
C	11.068292	5.024612	6.029903
H	11.632060	5.834039	5.556218
H	10.172700	4.831061	5.430011
H	11.681538	4.116629	5.998581
C	10.735737	5.354856	7.496686
C	12.046234	5.648525	8.237894
H	12.728327	4.801933	8.099572
H	11.891267	5.769482	9.312614
H	12.542239	6.544410	7.850837
C	10.026422	4.153851	8.136850
H	9.123844	3.863894	7.591547
H	9.757641	4.333856	9.178809
H	10.706631	3.293538	8.113937
C	9.031597	6.758277	10.217667
H	8.176124	6.126080	9.960077
H	8.758142	7.326345	11.115388
H	9.873411	6.114887	10.484293
C	9.391497	7.745630	9.102853
C	10.640175	8.550584	9.496606
H	11.513886	7.917214	9.659199
H	10.433910	9.076031	10.436096
H	10.890612	9.295344	8.737688
C	8.230112	8.735560	8.898790
H	8.107157	9.317899	9.818967
H	7.279490	8.230878	8.702339
H	8.431877	9.433744	8.080772

TS2a

P	10.126834	6.885392	7.356026
P	9.365715	9.904868	3.878837
O	10.798546	7.678196	6.242335
N	9.733378	9.734152	5.449636
C	8.410777	6.387575	6.837990
H	8.580760	5.757709	5.957981
H	7.950947	5.763597	7.608961
C	7.518816	9.884617	3.662003
H	7.123115	10.902720	3.646914
H	7.218043	9.403730	2.723217
N	7.839301	8.266722	5.406748
C	7.470852	7.473556	6.422202
C	6.191609	7.597502	6.971375
H	5.900474	6.966990	7.804604
C	5.301822	8.509067	6.423485
H	4.304882	8.615462	6.837840
C	5.686757	9.269276	5.323040
H	4.998677	9.955937	4.842718
C	6.983413	9.122892	4.845698
C	9.420990	7.343249	2.869377
H	8.348937	7.393939	2.657226
H	9.569835	6.971401	3.885157
H	9.859760	6.624595	2.168343
C	10.145248	8.684989	2.667361
C	9.981074	9.156984	1.218909

H	10.324997	8.356287	0.554972
H	10.587739	10.039331	1.001910
H	8.939012	9.369088	0.955665
C	11.622479	8.494057	3.035681
H	12.011855	7.662001	2.438331
H	11.734606	8.246107	4.094090
H	12.229607	9.370378	2.807226
C	9.441876	12.489116	4.968527
H	9.646102	13.556707	4.825256
H	9.970377	12.148868	5.861907
H	8.368316	12.374285	5.142117
C	9.918567	11.747343	3.710243
C	11.440223	11.839220	3.588617
H	11.802004	11.458582	2.630930
H	11.950625	11.319616	4.401928
H	11.711185	12.900307	3.638072
C	9.247840	12.350539	2.468697
H	9.505747	11.833538	1.543494
H	9.616433	13.380433	2.382228
H	8.160643	12.406232	2.554475
C	12.552557	5.556363	7.584203
H	12.838946	6.084178	8.494543
H	12.866379	6.151551	6.723061
H	13.099296	4.607321	7.566909
C	11.047149	5.244614	7.523080
C	10.614703	4.416916	8.737073
H	11.131033	3.450659	8.698244
H	9.540218	4.206759	8.741626
H	10.885190	4.888830	9.684305
C	10.782919	4.441472	6.235984
H	10.953064	5.045038	5.338631
H	9.774871	4.018270	6.204331
H	11.482651	3.599394	6.207410
C	8.888243	7.230468	9.882429
H	7.886397	7.294759	9.447842
H	8.870619	7.806082	10.814494
H	9.091553	6.190320	10.146010
C	9.949845	7.843041	8.957339
C	11.302273	7.924806	9.681752
H	11.612629	6.964605	10.100178
H	11.202833	8.628174	10.515700
H	12.091068	8.300193	9.023341
C	9.524185	9.271041	8.575939
H	9.407734	9.847170	9.501118
H	8.570662	9.297651	8.041337
H	10.271825	9.753418	7.945132

TS1b

P	0.176252	-1.479032	1.676206
P	0.081366	1.703714	-1.899075
O	1.220118	1.785026	0.642552
N	0.154351	1.761123	0.193510
C	-1.594300	-1.884573	1.181768
H	-1.507447	-2.669289	0.419460
H	-2.165424	-2.314219	2.011163
C	-1.718429	1.523791	-2.237547
H	-2.141419	2.503433	-2.472182
H	-1.832572	0.904313	-3.137021
N	-1.771163	-0.075208	-0.434738
C	-2.360043	-0.754298	0.558969
C	-3.669514	-0.454200	0.949175
H	-4.133721	-1.011765	1.755563
C	-4.359266	0.557230	0.299091
H	-5.371616	0.809509	0.596841
C	-3.744527	1.241634	-0.745332
H	-4.264057	2.025303	-1.285962
C	-2.445566	0.878337	-1.078613
C	0.351116	-0.969294	-2.653595
H	-0.565103	-0.949528	-3.250339
H	0.098511	-1.242752	-1.628597
H	1.000925	-1.744551	-3.074736
C	1.124394	0.353641	-2.710331
C	1.432262	0.706677	-4.171906
H	1.966523	-0.143852	-4.610635
H	2.077820	1.581518	-4.268806
H	0.524725	0.861395	-4.763748
C	2.418686	0.202351	-1.892360
H	3.037812	-0.555849	-2.384202
H	2.207257	-0.147339	-0.877654
H	3.006480	1.121471	-1.838746
C	-0.247189	4.377683	-1.320163
H	-0.082817	5.421705	-1.607149
H	0.106805	4.266011	-0.292249
H	-1.327092	4.204560	-1.336130
C	0.527021	3.487709	-2.314481
C	2.032016	3.724599	-2.126053
H	2.635048	3.190623	-2.863237
H	2.376856	3.452326	-1.124224
H	2.227274	4.794319	-2.258115
C	0.103837	3.849592	-3.747177
H	0.648530	3.284598	-4.503939
H	0.324837	4.911440	-3.904296
H	-0.968340	3.715173	-3.914064
C	1.227777	-3.508374	0.153182
H	1.905694	-2.757563	-0.265422
H	0.319133	-3.529824	-0.458988
H	1.705417	-4.490509	0.055054
C	0.931953	-3.234176	1.639172
C	2.270212	-3.213739	2.389812
H	2.810428	-4.146609	2.189601
H	2.135103	-3.143775	3.471731
H	2.906723	-2.383406	2.064516
C	0.053542	-4.369043	2.179562
H	-0.884546	-4.466944	1.625980
H	-0.183903	-4.248628	3.236979
H	0.590883	-5.319237	2.069830
C	-0.491843	-1.921731	4.501964
H	-1.412029	-2.431896	4.200589
H	-0.695865	-1.422165	5.457463
H	0.276626	-2.674871	4.690044
C	-0.042434	-0.879199	3.476020
C	1.290176	-0.253183	3.920180
H	2.095080	-0.987263	3.992097
H	1.164982	0.197009	4.912218
H	1.609124	0.534813	3.230360
C	-1.094003	0.242580	3.424544

H	-1.100659	0.769690	4.385796
H	-2.102158	-0.145987	3.257996
H	-0.876630	0.978974	2.642829

TS2b

P	-0.054919	-1.068902	1.331645
P	0.184948	1.121466	-1.284794
O	2.047532	0.340006	0.377247
N	0.769556	0.451530	0.384122
C	-1.596010	-2.139356	1.018374
H	-1.308475	-2.998761	0.401515
H	-1.990284	-2.539188	1.958656
C	-1.563839	1.803764	-1.306827
H	-1.595562	2.622511	-0.576930
H	-1.774037	2.237317	-2.289129
N	-1.950867	-0.150624	-0.110402
C	-2.553439	-1.271240	0.265804
C	-3.864556	-1.539669	-0.116684
H	-4.365680	-2.452886	0.184271
C	-4.509115	-0.595244	-0.914641
H	-5.534750	-0.765104	-1.225200
C	-3.838438	0.550236	-1.346883
H	-4.322988	1.268199	-1.999443
C	-2.526234	0.725948	-0.922700
C	-0.651764	-1.022866	-2.865708
H	-1.669551	-0.635873	-2.963463
H	-0.593303	-1.635250	-1.961552
H	-0.459282	-1.673346	-3.725580
C	0.410680	0.087710	-2.868814
C	0.250399	0.931435	-4.140587
H	0.295902	0.250781	-4.999108
H	1.056012	1.657360	-4.263001
H	-0.711547	1.449881	-4.192551
C	1.797768	-0.571242	-2.853847
H	1.872103	-1.220060	-3.733969
H	1.935546	-1.187706	-1.967218
H	2.612841	0.149424	-2.899721
C	1.221740	3.303137	0.138850
H	1.625548	4.319566	0.077582
H	1.862046	2.716488	0.797927
H	0.223778	3.376618	0.583327
C	1.204743	2.726867	-1.290107
C	2.652755	2.516018	-1.756171
H	2.715119	2.304921	-2.826496
H	3.151273	1.726829	-1.192558
H	3.194371	3.452575	-1.583038
C	0.534211	3.767924	-2.211074
H	0.368417	3.412402	-3.228950
H	1.212916	4.624823	-2.275605
H	-0.410234	4.141501	-1.808731
C	1.825772	-2.753612	0.202803
H	2.413383	-1.931568	-0.206496
H	1.023506	-3.020323	-0.494589
H	2.478100	-3.629016	0.296302
C	1.301348	-2.387971	1.601152
C	2.484509	-1.916170	2.460458
H	3.250073	-2.699645	2.420942
H	2.210447	-1.791413	3.510209
H	2.924298	-0.992452	2.084506
C	0.704886	-3.657313	2.241912
H	0.016462	-4.183370	1.577328
H	0.203436	-3.471526	3.192591
H	1.537524	-4.341633	2.441120
C	-0.656430	-1.119190	4.103583
H	-1.441024	-1.857350	3.916559
H	-0.968444	-0.538964	4.978682
H	0.263014	-1.639607	4.374036
C	-0.457005	-0.136803	2.937472
C	0.672790	0.843165	3.297898
H	1.642815	0.359520	3.403306
H	0.418433	1.299783	4.260694
H	0.765887	1.638228	2.558436
C	-1.750400	0.677067	2.768488
H	-1.924020	1.218492	3.704848
H	-2.626793	0.049213	2.584590
H	-1.667900	1.412536	1.965331

TS3b

P	12.158104	10.923927	2.891522
P	10.075578	10.303598	5.307000
C	13.666147	9.957887	3.506538
H	13.509481	8.910952	3.216818
H	14.559212	10.306759	2.982382
C	10.894899	10.779997	6.960340
H	10.729776	11.847099	7.146448
H	10.405899	10.231047	7.771702
N	12.560041	10.213017	5.512684
C	13.768706	10.073925	4.991027
C	14.893417	10.117532	5.807599
H	15.892421	9.992751	5.404769
C	14.692155	10.353954	7.169723
H	15.547804	10.396487	7.835562
C	13.412218	10.574160	7.681939
H	13.261982	10.809645	8.729329
C	12.349109	10.493948	6.788769
C	7.953685	11.330434	6.891543
H	7.777855	10.344146	7.320115
H	6.989364	11.849202	6.876424
H	8.603117	11.896729	7.562150
C	8.462094	11.308671	5.432524
C	8.795645	12.759632	5.041108
H	9.654884	13.161971	5.589219
H	7.931828	13.382246	5.298045
H	8.976292	12.824155	3.969163
C	7.329370	10.792702	4.531877
H	7.657456	10.679328	3.498310
H	6.523927	11.535090	4.559967
H	6.908192	9.853325	4.899450
C	8.813266	8.085935	6.574299
H	8.746559	6.993975	6.628524
H	7.801832	8.460258	6.412123
H	9.173697	8.430649	7.547578
C	9.760083	8.432102	5.414174
C	9.134728	7.956571	4.095511
H	8.954195	6.879165	4.177965
H	9.808149	8.123592	3.257979

H	8.184572	8.439943	3.872799
C	11.076737	7.668235	5.621087
H	11.562705	7.905759	6.571274
H	11.783773	7.848469	4.808543
H	10.840674	6.598722	5.627589
C	13.381192	9.918054	0.546197
H	13.229820	9.631688	-0.499523
H	13.868086	9.072752	1.037061
H	14.062909	10.770501	0.547654
C	11.986940	10.236362	1.127488
C	11.185719	8.931781	1.213947
H	11.192934	8.465208	0.222950
H	10.155038	9.146716	1.498660
H	11.627434	8.211392	1.911446
C	11.269903	11.200013	0.171907
H	11.876510	12.078102	-0.063455
H	10.306144	11.511778	0.571840
H	11.103830	10.665425	-0.769881
C	13.020845	13.116936	4.400354
H	13.912752	12.597470	4.758993
H	12.193468	12.922166	5.087830
H	13.230295	14.191357	4.428889
C	12.654361	12.749633	2.951434
C	13.877898	13.003749	2.055203
H	13.655640	12.866352	0.995669
H	14.745048	12.393074	2.322470
H	14.171462	14.050404	2.189327
C	11.505109	13.666566	2.506056
H	11.863667	14.700122	2.578686
H	10.635053	13.553425	3.148133
H	11.190773	13.487017	1.479676
N	10.702720	10.694100	3.735807
O	9.785123	11.132465	2.782592

TS4b

P	12.540884	11.079738	2.941566
P	9.855349	10.379946	5.022770
C	14.157382	10.349456	3.585042
H	14.330760	9.375303	3.116964
H	14.991683	10.998805	3.294696
C	10.830438	10.922155	6.643109
H	10.773383	12.017082	6.622969
H	10.313076	10.578165	7.537923
N	12.850196	10.727559	5.468963
C	13.993179	10.192105	5.073896
C	14.768538	9.488151	5.988205
H	15.721095	9.050272	5.712409
C	14.254255	9.347212	7.281508
H	14.834927	8.814454	8.027342
C	12.983966	9.818079	7.611246
H	12.557412	9.631203	8.590850
C	12.257127	10.500246	6.632300
C	7.924030	11.509870	6.915836
H	7.834616	10.545994	7.419802
H	6.947841	12.002585	6.986959
H	8.630300	12.131314	7.470892
C	8.281649	11.391964	5.425904
C	8.540013	12.810341	4.884257
H	9.430685	13.276248	5.322527
H	7.685808	13.443386	5.149846
H	8.637069	12.805688	3.797228
C	7.078599	10.807760	4.668519
H	7.307976	10.619478	3.616651
H	6.256790	11.532186	4.709150
H	6.716351	9.884532	5.128182
C	8.993816	8.192841	6.642619
H	8.958520	7.103581	6.754375
H	7.964777	8.554052	6.696899
H	9.553245	8.582872	7.496745
C	9.649797	8.512110	5.291081
C	8.780072	7.902723	4.178531
H	8.730773	6.819991	4.338960
H	9.193393	8.083049	3.186584
H	7.759394	8.284668	4.189733
C	11.030346	7.834940	5.248305
H	11.661285	8.119710	6.093075
H	11.565976	8.052536	4.323070
H	10.881538	6.750796	5.303632
C	13.504117	10.164748	0.457146
H	13.259021	9.631942	-0.467940
H	14.310679	9.607029	0.940009
H	13.877969	11.147748	0.172704
C	12.217162	10.215570	1.301995
C	11.791208	8.777358	1.630463
H	11.652475	8.243614	0.684189
H	10.849941	8.763721	2.178325
H	12.550793	8.232676	2.199971
C	11.103923	10.911051	0.504698
H	11.427278	11.876086	0.107991
H	10.197691	11.042540	1.098138
H	10.860565	10.275295	-0.353490
C	13.361010	13.447793	4.147535
H	14.335682	13.016302	4.392326
H	12.675146	13.257898	4.975452
H	13.495228	14.531037	4.060299
C	12.796238	12.939520	2.809414
C	13.770685	13.303607	1.680766
H	13.347122	13.116613	0.692177
H	14.733576	12.788850	1.759602
H	13.974131	14.377669	1.746202
C	11.437195	13.613803	2.574843
H	11.583302	14.698683	2.611794
H	10.724451	13.340083	3.352845
H	11.003470	13.369355	1.605094
N	11.375895	10.732661	4.083807
O	9.748050	10.543963	3.383400

Computed IR spectrum of [DAPP^{tBu}]$^{+}$ (crystal) at the RI-r^{2}SCAN-3c(D4)/def2-mTZVPP level of theory

Mode	freq cm**-1	eps L/(mol*cm)	Int km/mol	T**2 a.u.	TX	TY	TZ
6:	25.58	0.000115	0.58	0.001406	(-0.004040	-0.036292	0.008523)
7:	34.37	0.000125	0.63	0.001131	(0.025493	-0.002757	-0.021762)
8:	40.03	0.000017	0.08	0.000129	(-0.005083	-0.000702	-0.010119)
9:	49.34	0.000074	0.37	0.000469	(-0.010127	0.017657	-0.007409)
10:	70.74	0.000047	0.24	0.000207	(0.012214	0.000096	0.007578)
11:	81.31	0.000048	0.24	0.000183	(0.011717	-0.005793	0.003554)
12:	89.62	0.000046	0.23	0.000161	(0.008164	-0.007310	-0.006398)
13:	90.61	0.000029	0.15	0.000099	(0.000302	-0.009750	-0.001946)
14:	92.79	0.000050	0.25	0.000168	(-0.008110	0.007794	0.006408)
15:	126.99	0.000873	4.41	0.002146	(0.029346	0.006624	-0.035222)
16:	142.53	0.000008	0.04	0.000017	(-0.001395	0.003866	-0.000344)
17:	152.94	0.000176	0.89	0.000360	(-0.001668	-0.001176	-0.018855)
18:	161.25	0.000106	0.53	0.000204	(-0.005611	-0.001687	0.013034)
19:	183.01	0.000049	0.25	0.000083	(-0.002816	0.008673	-0.000167)
20:	200.36	0.000070	0.35	0.000109	(0.006246	0.002307	-0.008031)
21:	202.56	0.000199	1.00	0.000306	(-0.016391	-0.001350	0.005977)
22:	210.17	0.000748	3.78	0.001110	(-0.016511	0.026234	0.012228)
23:	220.29	0.000416	2.10	0.000590	(-0.005458	0.023631	0.001294)
24:	226.95	0.000594	3.00	0.000816	(-0.003706	0.026083	-0.011057)
25:	231.64	0.000032	0.16	0.000043	(0.002126	0.001786	-0.005923)
26:	235.50	0.000766	3.87	0.001014	(-0.017836	-0.019806	0.017437)
27:	246.16	0.000160	0.81	0.000203	(-0.008347	-0.008962	0.007303)
28:	247.79	0.000228	1.15	0.000287	(0.002025	0.014769	0.008038)
29:	248.38	0.000157	0.79	0.000197	(-0.007680	-0.008472	0.008122)
30:	257.08	0.000439	2.22	0.000533	(0.016187	-0.015545	-0.005413)
31:	265.78	0.000206	1.04	0.000242	(-0.010057	-0.007509	0.009167)
32:	269.78	0.000187	0.95	0.000216	(0.005821	0.011273	-0.007442)
33:	271.90	0.001656	8.37	0.001900	(0.030332	-0.026240	-0.017075)
34:	284.20	0.000366	1.85	0.000402	(0.005514	0.004623	-0.018720)
35:	285.10	0.000294	1.49	0.000322	(0.004668	0.013348	-0.011048)
36:	293.22	0.000687	3.47	0.000731	(-0.022358	-0.010195	0.011300)
37:	297.45	0.001401	7.08	0.001470	(-0.003597	0.038040	-0.003119)
38:	306.02	0.000182	0.92	0.000185	(0.004874	0.011488	-0.005453)
39:	312.63	0.000218	1.10	0.000218	(0.008829	-0.010975	-0.004422)
40:	318.98	0.000063	0.32	0.000062	(0.005461	-0.004346	-0.003621)
41:	324.51	0.000064	0.32	0.000061	(-0.007171	-0.001531	0.002749)
42:	326.17	0.000015	0.08	0.000014	(0.003697	-0.000777	-0.000389)
43:	328.79	0.000010	0.05	0.000009	(0.001971	-0.002134	-0.000933)
44:	332.84	0.000029	0.14	0.000027	(0.001798	0.004023	-0.002714)
45:	343.84	0.000037	0.19	0.000034	(-0.000466	0.005485	0.001867)
46:	352.48	0.000255	1.29	0.000226	(-0.007406	0.012639	0.003340)
47:	358.20	0.000204	1.03	0.000178	(-0.009692	-0.007976	0.004531)
48:	382.26	0.001455	7.35	0.001187	(-0.022968	0.012271	0.022568)
49:	392.26	0.000339	1.71	0.000270	(-0.013965	-0.008358	-0.002185)
50:	400.80	0.000180	0.91	0.000140	(-0.000129	0.011504	0.002742)
51:	404.81	0.000084	0.42	0.000064	(0.002973	-0.004033	-0.006267)
52:	405.54	0.000172	0.87	0.000132	(0.009854	-0.005922	-0.000378)
53:	409.83	0.000046	0.23	0.000035	(-0.000903	-0.005253	-0.002564)
54:	414.00	0.000359	1.81	0.000270	(0.013113	-0.003509	0.009283)
55:	439.52	0.002055	10.38	0.001459	(0.010893	-0.035372	-0.009443)
56:	445.32	0.001366	6.90	0.000957	(0.013982	-0.027328	0.003849)
57:	454.90	0.002126	10.74	0.001458	(-0.005480	0.037734	-0.002112)
58:	471.72	0.001052	5.32	0.000696	(0.019569	0.008381	-0.015584)
59:	488.49	0.002464	12.45	0.001574	(-0.036082	-0.012312	0.010995)
60:	504.33	0.006060	30.62	0.003749	(-0.028600	-0.010415	0.053132)
61:	518.78	0.002627	13.27	0.001580	(-0.037875	0.005710	0.010622)
62:	520.94	0.001574	7.96	0.000943	(-0.027703	-0.001687	0.013144)
63:	561.75	0.001199	6.06	0.000666	(0.019948	0.000725	-0.016353)
64:	583.58	0.000992	5.01	0.000530	(0.019204	-0.008463	0.009483)

65:	596.78	0.001656	8.37	0.000866	(0.019423	-0.010269	-0.019575)
66:	610.97	0.002252	11.38	0.001150	(-0.013798	-0.022667	-0.021118)
67:	627.41	0.002212	11.18	0.001100	(-0.029714	-0.010227	0.010612)
68:	651.86	0.012197	61.64	0.005839	(0.019073	0.051136	-0.053482)
69:	682.73	0.009921	50.14	0.004535	(0.043185	-0.023955	-0.045780)
70:	719.73	0.003494	17.65	0.001515	(-0.035704	-0.010521	0.011369)
71:	747.65	0.002363	11.94	0.000986	(-0.023981	0.019542	0.005409)
72:	757.95	0.001682	8.50	0.000693	(-0.025016	0.003165	-0.007533)
73:	788.38	0.004464	22.56	0.001767	(0.014677	-0.036865	-0.013879)
74:	805.42	0.002229	11.26	0.000864	(-0.002619	-0.009527	-0.027677)
75:	807.40	0.001673	8.45	0.000646	(-0.011047	0.017669	-0.014568)
76:	813.09	0.003570	18.04	0.001370	(-0.025555	-0.020127	0.017664)
77:	815.37	0.000631	3.19	0.000241	(0.008798	-0.011115	-0.006363)
78:	825.43	0.001023	5.17	0.000387	(-0.007486	0.011878	-0.013775)
79:	828.40	0.004875	24.64	0.001837	(0.009736	0.039193	-0.014343)
80:	841.26	0.015298	77.31	0.005675	(-0.023417	0.069486	0.017267)
81:	918.82	0.000215	1.09	0.000073	(-0.005525	-0.006508	0.000245)
82:	942.01	0.012334	62.33	0.004086	(0.013177	0.010243	-0.061704)
83:	942.36	0.000552	2.79	0.000183	(-0.001473	-0.005749	-0.012154)
84:	942.91	0.026633	134.59	0.008814	(-0.012046	-0.022250	0.090412)
85:	945.86	0.001858	9.39	0.000613	(-0.010985	0.007531	-0.020868)
86:	946.58	0.002137	10.80	0.000705	(0.004194	-0.009722	0.024343)
87:	949.69	0.000697	3.52	0.000229	(-0.007248	0.001896	0.013155)
88:	950.78	0.000025	0.12	0.000008	(-0.001837	-0.001177	0.001826)
89:	951.72	0.001170	5.91	0.000384	(-0.009650	0.003786	-0.016621)
90:	955.18	0.000406	2.05	0.000133	(0.001825	-0.002598	0.011068)
91:	968.06	0.000085	0.43	0.000027	(0.000462	0.001905	0.004840)
92:	971.40	0.000304	1.54	0.000098	(0.000904	-0.003816	0.009073)
93:	972.76	0.000122	0.62	0.000039	(0.005777	-0.001580	0.001806)
94:	976.58	0.000035	0.18	0.000011	(0.003093	-0.000565	-0.001163)
95:	983.55	0.011337	57.29	0.003597	(0.024843	0.019309	-0.051060)
96:	989.99	0.001041	5.26	0.000328	(-0.014321	0.000241	0.011089)
97:	1022.12	0.001331	6.73	0.000406	(-0.002801	0.019708	-0.003179)
98:	1022.25	0.000115	0.58	0.000035	(-0.005015	-0.002212	0.002268)
99:	1030.09	0.000610	3.08	0.000185	(-0.002550	-0.010994	0.007578)
100:	1031.53	0.000936	4.73	0.000283	(0.005682	0.000591	-0.015827)
101:	1036.27	0.000964	4.87	0.000290	(-0.011779	0.005211	0.011151)
102:	1038.08	0.001249	6.31	0.000376	(0.002984	-0.005911	0.018214)
103:	1039.89	0.002076	10.49	0.000623	(0.011250	0.005015	-0.021706)
104:	1042.21	0.000588	2.97	0.000176	(-0.012077	-0.002061	0.005086)
105:	1097.99	0.002698	13.63	0.000767	(-0.024346	0.008078	-0.010430)
106:	1128.56	0.001440	7.28	0.000398	(0.004732	-0.015155	-0.012091)
107:	1150.82	0.002093	10.58	0.000568	(0.009796	0.011068	0.018686)
108:	1170.97	0.045564	230.26	0.012143	(0.040926	-0.010404	0.101782)
109:	1179.03	0.000166	0.84	0.000044	(0.001833	-0.005371	0.003436)
110:	1191.93	0.003054	15.43	0.000800	(0.020213	0.003909	0.019385)
111:	1197.81	0.021582	109.07	0.005623	(0.043502	0.020159	-0.057653)
112:	1203.16	0.005715	28.88	0.001482	(-0.014224	0.033236	0.013244)
113:	1204.75	0.003090	15.62	0.000800	(0.006604	-0.011610	0.024941)
114:	1208.10	0.000249	1.26	0.000064	(-0.002518	0.004997	-0.005743)
115:	1211.62	0.004604	23.27	0.001186	(0.003600	0.018945	-0.028529)
116:	1212.37	0.000442	2.23	0.000114	(0.002917	-0.010212	-0.001002)
117:	1214.19	0.003703	18.71	0.000952	(0.014808	-0.014491	-0.022854)
118:	1218.50	0.006199	31.33	0.001588	(0.009467	0.005793	-0.038268)
119:	1219.07	0.002688	13.58	0.000688	(-0.002621	0.016417	-0.020291)
120:	1223.57	0.001618	8.18	0.000413	(0.012660	0.015423	0.003798)
121:	1225.85	0.001155	5.83	0.000294	(-0.002150	-0.007462	0.015285)
122:	1228.25	0.001993	10.07	0.000506	(-0.010724	-0.001513	0.019725)
123:	1233.11	0.002250	11.37	0.000569	(0.002109	-0.009279	0.021881)
124:	1236.31	0.000660	3.34	0.000167	(0.010833	-0.006028	-0.003597)
125:	1251.89	0.004048	20.46	0.001009	(0.018386	0.006555	-0.025063)
126:	1292.92	0.004941	24.97	0.001193	(-0.033323	-0.000380	-0.009064)
127:	1328.18	0.001939	9.80	0.000456	(-0.021242	-0.000930	-0.001861)
128:	1384.85	0.000659	3.33	0.000149	(0.008687	-0.000725	-0.008518)

129:	1385.25	0.000161	0.81	0.000036	(0.003753 -0.004248 -0.002016)
130:	1390.16	0.001035	5.23	0.000232	(-0.002592 -0.007219 -0.013173)
131:	1392.76	0.002976	15.04	0.000667	(0.007182 0.007841 0.023533)
132:	1395.63	0.001873	9.47	0.000419	(0.011754 -0.016724 -0.001024)
133:	1397.39	0.001055	5.33	0.000236	(0.014547 -0.001761 0.004580)
134:	1399.74	0.000678	3.42	0.000151	(-0.001605 0.005963 -0.010627)
135:	1402.80	0.004506	22.77	0.001002	(-0.028972 -0.012642 0.001793)
136:	1416.41	0.006183	31.24	0.001362	(0.007421 0.021456 -0.029098)
137:	1420.45	0.002070	10.46	0.000455	(-0.001142 0.021265 -0.001143)
138:	1428.84	0.002730	13.80	0.000596	(-0.009130 0.022123 0.004844)
139:	1431.85	0.002149	10.86	0.000468	(-0.021428 0.001701 -0.002529)
140:	1433.96	0.002025	10.23	0.000441	(-0.001678 0.008696 -0.019031)
141:	1438.17	0.009541	48.22	0.002070	(0.019147 0.002732 0.041186)
142:	1440.31	0.001133	5.73	0.000246	(-0.014348 0.006239 0.000840)
143:	1468.93	0.000045	0.23	0.000010	(-0.002654 0.000095 -0.001592)
144:	1469.89	0.000030	0.15	0.000006	(0.001904 0.000388 -0.001643)
145:	1470.72	0.000190	0.96	0.000040	(0.001253 0.005364 -0.003167)
146:	1472.06	0.000497	2.51	0.000105	(0.000324 0.009813 -0.002984)
147:	1474.66	0.000207	1.05	0.000044	(-0.006411 0.001229 -0.001137)
148:	1477.21	0.000182	0.92	0.000039	(0.005374 -0.003051 0.000582)
149:	1479.83	0.000160	0.81	0.000034	(0.004904 -0.000212 0.003097)
150:	1480.31	0.000447	2.26	0.000094	(-0.005511 0.001255 0.007891)
151:	1482.56	0.000529	2.67	0.000111	(0.001483 -0.001802 0.010287)
152:	1483.10	0.000327	1.65	0.000069	(0.005673 0.004534 -0.003997)
153:	1483.93	0.000336	1.70	0.000071	(-0.005561 0.002791 0.005644)
154:	1488.15	0.001959	9.90	0.000411	(0.010325 -0.000999 -0.017413)
155:	1496.82	0.002147	10.85	0.000448	(-0.012298 -0.002374 0.017053)
156:	1497.80	0.002122	10.72	0.000442	(-0.013713 -0.015931 0.000483)
157:	1499.01	0.000799	4.04	0.000166	(0.001299 -0.004798 0.011903)
158:	1501.21	0.000704	3.56	0.000146	(0.005806 0.006528 0.008373)
159:	1506.57	0.004737	23.94	0.000981	(0.026434 0.015431 0.006664)
160:	1508.17	0.002235	11.30	0.000462	(-0.003644 -0.002283 -0.021071)
161:	1509.13	0.004693	23.72	0.000970	(0.027511 -0.008207 -0.012091)
162:	1511.94	0.002548	12.87	0.000526	(0.011913 -0.008126 0.017828)
163:	1516.85	0.009696	49.00	0.001995	(-0.039973 0.004403 0.019430)
164:	1518.33	0.005385	27.21	0.001107	(-0.010356 -0.031588 -0.001316)
165:	1518.53	0.005810	29.36	0.001194	(0.013642 0.031559 -0.003454)
166:	1520.57	0.002218	11.21	0.000455	(0.006192 -0.002859 -0.020217)
167:	1524.45	0.003321	16.79	0.000680	(0.018685 -0.017106 0.006180)
168:	1588.46	0.005119	25.87	0.001006	(0.026838 0.006612 -0.015546)
169:	1647.60	0.005047	25.51	0.000956	(-0.028544 -0.004449 0.011017)
170:	3007.30	0.001686	8.52	0.000175	(0.009273 0.009432 -0.000141)
171:	3011.40	0.002740	13.85	0.000284	(0.001636 -0.005653 0.015790)
172:	3017.25	0.003445	17.41	0.000356	(0.012222 -0.014383 0.000191)
173:	3019.78	0.002307	11.66	0.000238	(-0.010470 -0.000581 0.011335)
174:	3021.23	0.001074	5.43	0.000111	(0.006276 -0.001926 -0.008238)
175:	3022.38	0.003024	15.28	0.000312	(-0.005598 0.005932 -0.015675)
176:	3023.48	0.004739	23.95	0.000489	(0.007526 0.018532 0.009435)
177:	3024.04	0.000861	4.35	0.000089	(-0.007059 -0.006067 -0.001474)
178:	3024.61	0.001943	9.82	0.000200	(-0.009220 -0.010553 -0.002025)
179:	3026.36	0.004325	21.86	0.000446	(-0.014940 0.014415 0.003873)
180:	3028.47	0.002860	14.45	0.000295	(-0.012723 0.000256 0.011521)
181:	3032.90	0.001435	7.25	0.000148	(0.009522 -0.003947 0.006431)
182:	3047.33	0.000084	0.42	0.000009	(0.002617 0.000995 -0.000860)
183:	3066.55	0.000033	0.17	0.000003	(0.000058 0.001840 0.000013)
184:	3080.85	0.002999	15.16	0.000304	(-0.004626 0.015734 0.005903)
185:	3086.47	0.002648	13.38	0.000268	(0.008693 -0.013562 -0.002864)
186:	3089.73	0.002125	10.74	0.000215	(-0.013535 0.000609 0.005578)
187:	3094.29	0.005627	28.44	0.000567	(0.017662 -0.000609 -0.015974)
188:	3097.29	0.000583	2.95	0.000059	(0.001549 0.006512 0.003738)
189:	3097.81	0.004840	24.46	0.000488	(-0.011756 -0.017378 0.006880)
190:	3098.89	0.001312	6.63	0.000132	(-0.010186 0.004912 -0.002051)
191:	3100.52	0.002094	10.58	0.000211	(-0.010824 -0.002547 -0.009332)
192:	3103.21	0.002868	14.49	0.000288	(-0.002375 0.010475 0.013154)

193:	3105.59	0.002009	10.15	0.000202	(-0.011373	0.003613	-0.007713)
194:	3108.40	0.000789	3.99	0.000079	(0.000368	0.006653	-0.005900)
195:	3110.68	0.000934	4.72	0.000094	(-0.003830	-0.002036	-0.008651)
196:	3114.53	0.001099	5.55	0.000110	(0.000626	0.010187	-0.002439)
197:	3114.71	0.000105	0.53	0.000010	(0.000303	0.003085	-0.000942)
198:	3115.26	0.004515	22.81	0.000452	(0.008303	0.010234	-0.016690)
199:	3118.45	0.001088	5.50	0.000109	(-0.008588	-0.003665	0.004660)
200:	3119.63	0.001247	6.30	0.000125	(0.010464	-0.002381	0.003099)
201:	3120.23	0.000707	3.57	0.000071	(-0.007718	0.000923	0.003205)
202:	3121.33	0.002647	13.38	0.000265	(-0.006898	-0.014732	0.000097)
203:	3125.25	0.000939	4.75	0.000094	(0.002593	0.000349	0.009323)
204:	3129.07	0.001988	10.05	0.000198	(-0.010329	0.009280	-0.002335)
205:	3129.36	0.001451	7.33	0.000145	(0.005745	-0.006711	-0.008165)
206:	3138.20	0.000883	4.46	0.000088	(-0.001062	0.004345	0.008235)
207:	3139.86	0.002574	13.01	0.000256	(-0.004662	0.004450	-0.014639)
208:	3141.37	0.001990	10.06	0.000198	(0.013128	0.004786	-0.001558)
209:	3143.10	0.001583	8.00	0.000157	(-0.009298	-0.004494	0.007107)
210:	3200.65	0.000029	0.15	0.000003	(0.001203	0.000485	-0.001061)
211:	3212.24	0.000697	3.52	0.000068	(-0.005350	-0.000908	0.006188)
212:	3221.49	0.001883	9.51	0.000182	(0.007059	0.001056	-0.011463)

Computed Raman spectrum of $[DAPP^{tBu}]^+$ (crystal) at the RI-r^{2}SCAN-3c(D4)/def2-mTZVPP level of theory

Mode	freq (cm**-1)	Activity	Depolarization
6:	25.58	0.675063	0.722113
7:	34.37	1.339014	0.744133
8:	40.03	0.731526	0.688109
9:	49.34	2.884955	0.743803
10:	70.74	0.157022	0.736380
11:	81.31	1.504911	0.645020
12:	89.62	1.301414	0.742982
13:	90.61	0.290189	0.534020
14:	92.79	0.571990	0.749589
15:	126.99	1.629795	0.420569
16:	142.53	0.406039	0.688404
17:	152.94	1.189643	0.561770
18:	161.25	0.275493	0.735466
19:	183.01	0.567708	0.723482
20:	200.36	1.369825	0.164646
21:	202.56	1.558542	0.382658
22:	210.17	0.180720	0.711133
23:	220.29	1.022794	0.744264
24:	226.95	3.185019	0.051293
25:	231.64	1.255417	0.230799
26:	235.50	1.362396	0.194033
27:	246.16	1.289432	0.373336
28:	247.79	0.968916	0.168838
29:	248.38	2.090089	0.460043
30:	257.08	4.456255	0.097334
31:	265.78	2.899755	0.232834
32:	269.78	0.672468	0.748354
33:	271.90	6.482551	0.172039
34:	284.20	1.882091	0.723255
35:	285.10	5.908416	0.215739
36:	293.22	1.427858	0.737836
37:	297.45	1.735182	0.692021
38:	306.02	2.523384	0.300685
39:	312.63	1.775430	0.435414
40:	318.98	1.037102	0.706075
41:	324.51	0.621595	0.518510
42:	326.17	0.453831	0.273487
43:	328.79	0.631913	0.543082
44:	332.84	0.969292	0.311808
45:	343.84	0.083533	0.650037
46:	352.48	2.929462	0.196007
47:	358.20	0.733279	0.154264
48:	382.26	0.341523	0.641554
49:	392.26	2.227657	0.179047
50:	400.80	0.564292	0.324899
51:	404.81	0.341725	0.648555
52:	405.54	1.273259	0.336195
53:	409.83	0.222180	0.186728
54:	414.00	0.672039	0.749766
55:	439.52	0.955427	0.690220
56:	445.32	4.035188	0.539514
57:	454.90	1.034953	0.747608
58:	471.72	2.262547	0.740403
59:	488.49	1.344697	0.332165
60:	504.33	10.428282	0.249155
61:	518.78	21.304466	0.031115
62:	520.94	6.948649	0.017396
63:	561.75	5.031152	0.222479
64:	583.58	9.576718	0.176851
65:	596.78	7.597742	0.392057
66:	610.97	10.690165	0.287285
67:	627.41	6.874119	0.541365
68:	651.86	1.694453	0.742790
69:	682.73	18.793590	0.254593
70:	719.73	14.323735	0.247753
71:	747.65	6.723252	0.066529
72:	757.95	4.678266	0.322787
73:	788.38	1.095981	0.232722
74:	805.42	6.268273	0.736690
75:	807.40	5.721150	0.746989
76:	813.09	9.684754	0.741327
77:	815.37	16.045020	0.365362
78:	825.43	14.431788	0.326612
79:	828.40	1.658399	0.411294
80:	841.26	1.673599	0.541889
81:	918.82	1.660940	0.615917
82:	942.01	4.373800	0.700226
83:	942.36	1.364116	0.710847
84:	942.91	0.426521	0.160315
85:	945.86	4.738014	0.735820
86:	946.58	1.517941	0.714748
87:	949.69	3.039615	0.747589
88:	950.78	1.359568	0.735757
89:	951.72	3.874067	0.746016
90:	955.18	2.357121	0.749118
91:	968.06	0.411688	0.632609
92:	971.40	0.023036	0.465839
93:	972.76	0.158407	0.748674
94:	976.58	0.118457	0.747302
95:	983.55	16.854660	0.364728
96:	989.99	1.276300	0.427851
97:	1022.12	0.378668	0.450531
98:	1022.25	2.158709	0.697655
99:	1030.09	1.660426	0.706917
100:	1031.53	1.817354	0.745322
101:	1036.27	2.661938	0.338143
102:	1038.08	1.560851	0.749270
103:	1039.89	1.572125	0.548592
104:	1042.21	0.995749	0.719904
105:	1097.99	18.907079	0.061203
106:	1128.56	3.178425	0.564755
107:	1150.82	11.142488	0.184047
108:	1170.97	49.627610	0.158361
109:	1179.03	24.153007	0.298683
110:	1191.93	44.207058	0.198595
111:	1197.81	51.726114	0.509340
112:	1203.16	6.721218	0.635553
113:	1204.75	12.076503	0.421897
114:	1208.10	1.037068	0.741826
115:	1211.62	3.378389	0.597514
116:	1212.37	1.538453	0.101805
117:	1214.19	22.166070	0.276931
118:	1218.50	6.983492	0.586195
119:	1219.07	2.259789	0.738060
120:	1223.57	5.748853	0.559347
121:	1225.85	1.658601	0.480537
122:	1228.25	9.556937	0.684887
123:	1233.11	2.485431	0.335241
124:	1236.31	11.332188	0.151493
125:	1251.89	33.110533	0.331680
126:	1292.92	79.672171	0.177509
127:	1328.18	16.414995	0.329139
128:	1384.85	0.115538	0.619178
129:	1385.25	1.611854	0.746218
130:	1390.16	1.562835	0.617597
131:	1392.76	0.711489	0.650379
132:	1395.63	0.957684	0.736689
133:	1397.39	1.571335	0.706586
134:	1399.74	1.390450	0.580810
135:	1402.80	2.355629	0.671466
136:	1416.41	2.839861	0.741579
137:	1420.45	0.468008	0.445385
138:	1428.84	0.635294	0.746090
139:	1431.85	2.347003	0.428115
140:	1433.96	1.588009	0.393320
141:	1438.17	14.641321	0.437737
142:	1440.31	8.494374	0.658466
143:	1468.93	1.976583	0.748581
144:	1469.89	3.818494	0.749948
145:	1470.72	2.150540	0.743008
146:	1472.06	4.523753	0.738007
147:	1474.66	7.295465	0.749493
148:	1477.21	8.852558	0.749629
149:	1479.83	2.145637	0.749792
150:	1480.31	5.850586	0.705248
151:	1482.56	2.392525	0.717901
152:	1483.10	2.991152	0.746857
153:	1483.93	8.072557	0.749911
154:	1488.15	4.886494	0.739438
155:	1496.82	17.031045	0.719745
156:	1497.80	5.272432	0.658914
157:	1499.01	4.703490	0.749945
158:	1501.21	8.136854	0.749045
159:	1506.57	2.139408	0.448095
160:	1508.17	6.837808	0.749940

161:	1509.13	9.282347	0.732149
162:	1511.94	13.413808	0.717969
163:	1516.85	20.641832	0.651152
164:	1518.33	3.927842	0.465298
165:	1518.53	1.556414	0.660738
166:	1520.57	2.448187	0.727068
167:	1524.45	1.175608	0.364227
168:	1588.46	43.935805	0.259231
169:	1647.60	101.977541	0.732986
170:	3007.30	26.685571	0.115453
171:	3011.40	129.780713	0.027122
172:	3017.25	26.295888	0.190972
173:	3019.78	72.114375	0.134050
174:	3021.23	21.294361	0.172336
175:	3022.38	9.822503	0.674493
176:	3023.48	258.400099	0.081170
177:	3024.04	99.602692	0.052221
178:	3024.61	120.890442	0.082895
179:	3026.36	266.794101	0.029526
180:	3028.47	380.034466	0.049765
181:	3032.90	279.116209	0.025788
182:	3047.33	160.186359	0.137809
183:	3066.55	150.985712	0.055047
184:	3080.85	29.321487	0.732962
185:	3086.47	48.705171	0.749787
186:	3089.73	24.606754	0.641555
187:	3094.29	49.808434	0.736236
188:	3097.29	35.941580	0.731579
189:	3097.81	122.597365	0.646930
190:	3098.89	53.894621	0.576075
191:	3100.52	38.595782	0.563270
192:	3103.21	120.223136	0.599902
193:	3105.59	67.162116	0.692341
194:	3108.40	39.606956	0.720320
195:	3110.68	40.397385	0.628695
196:	3114.53	50.841895	0.738806
197:	3114.71	32.840023	0.749568
198:	3115.26	46.072644	0.661998
199:	3118.45	53.933749	0.712880
200:	3119.63	20.065023	0.688045
201:	3120.23	33.016125	0.747025
202:	3121.33	30.228098	0.749195
203:	3125.25	18.185689	0.677731
204:	3129.07	17.130130	0.715694
205:	3129.36	42.288732	0.748756
206:	3138.20	34.518910	0.735896
207:	3139.86	27.036578	0.646984
208:	3141.37	32.612888	0.630108
209:	3143.10	15.683152	0.434337
210:	3200.65	50.434236	0.574055
211:	3212.24	94.948690	0.445498
212:	3221.49	169.578556	0.126619

Computed IR spectrum of $[PNOP^{tBu}]^+$ at the RI-r^{2}SCAN-3c(D4)/def2-mTZVPP level of theory

Mode	freq cm**-1	eps L/(mol*cm)	Int km/mol	T**2 a.u.	TX	TY	TZ
6:	26.21	0.000083	0.42	0.000983	(-0.027877	-0.013868	0.003644)
7:	49.39	0.000010	0.05	0.000062	(0.005489	-0.005354	0.001826)
8:	64.76	0.000127	0.64	0.000612	(-0.022881	-0.007277	-0.006001)
9:	68.83	0.000100	0.50	0.000453	(0.016588	0.006390	0.011690)
10:	81.81	0.000069	0.35	0.000264	(0.010251	0.012482	0.001830)
11:	101.84	0.000038	0.19	0.000115	(0.002048	-0.007989	0.006873)
12:	105.98	0.000230	1.16	0.000676	(0.021332	-0.001747	0.014767)
13:	115.95	0.000046	0.23	0.000123	(-0.007005	-0.005526	0.006573)
14:	118.54	0.000004	0.02	0.000011	(-0.001122	-0.003147	-0.000445)
15:	131.54	0.000150	0.76	0.000355	(-0.011794	-0.000238	-0.014698)
16:	146.42	0.000393	1.98	0.000837	(-0.024562	-0.012506	-0.008773)
17:	155.53	0.000264	1.33	0.000530	(0.016493	0.008496	0.013622)
18:	175.12	0.000053	0.27	0.000094	(-0.000909	0.009498	0.001762)
19:	183.23	0.000239	1.21	0.000407	(0.001140	-0.010859	0.016954)
20:	197.44	0.000112	0.57	0.000177	(0.001403	-0.012493	0.004371)
21:	200.79	0.000003	0.02	0.000005	(0.000283	0.001352	0.001865)
22:	204.61	0.000145	0.73	0.000221	(0.004069	-0.014014	0.002926)
23:	215.40	0.000085	0.43	0.000123	(0.002584	-0.010714	0.001047)
24:	220.92	0.000095	0.48	0.000134	(0.001781	-0.011155	0.002592)
25:	228.46	0.000175	0.88	0.000239	(0.002248	0.015273	-0.000777)
26:	236.58	0.000083	0.42	0.000109	(-0.009412	0.002415	0.003799)
27:	242.10	0.000535	2.70	0.000690	(0.009836	0.007875	0.023047)
28:	243.35	0.000183	0.93	0.000235	(-0.006898	-0.007967	0.011135)
29:	252.41	0.000325	1.64	0.000402	(-0.011638	-0.012428	-0.010599)
30:	256.09	0.000485	2.45	0.000591	(-0.007994	-0.017111	-0.015291)
31:	261.75	0.000541	2.73	0.000645	(-0.005890	0.004093	0.024355)
32:	266.21	0.000218	1.10	0.000256	(-0.015991	0.000445	-0.000032)
33:	278.71	0.000484	2.45	0.000542	(0.021832	0.007795	-0.002184)
34:	282.65	0.000784	3.96	0.000866	(-0.024922	-0.001874	-0.015528)
35:	286.40	0.000135	0.68	0.000147	(-0.010838	0.002298	-0.004941)
36:	291.13	0.000592	2.99	0.000635	(-0.020000	0.010653	0.011025)
37:	295.48	0.000619	3.13	0.000654	(-0.021873	-0.010854	-0.007608)
38:	305.36	0.000060	0.31	0.000062	(-0.007451	0.002347	-0.000866)
39:	318.05	0.001738	8.78	0.001706	(0.027860	-0.015899	-0.026011)
40:	323.12	0.000228	1.15	0.000220	(0.012981	-0.003178	-0.006439)
41:	329.93	0.000065	0.33	0.000062	(0.000148	-0.007562	-0.002095)
42:	333.97	0.000132	0.67	0.000123	(0.006534	0.004530	0.007735)
43:	337.50	0.000086	0.43	0.000079	(0.008681	-0.000739	-0.001847)
44:	340.35	0.000336	1.70	0.000308	(0.003840	-0.011423	-0.012775)
45:	351.25	0.000018	0.09	0.000016	(-0.002520	-0.002593	-0.001722)
46:	359.12	0.000769	3.89	0.000668	(0.020214	0.010060	-0.012594)
47:	361.93	0.000210	1.06	0.000181	(-0.008648	-0.008517	0.005781)
48:	369.35	0.000953	4.82	0.000805	(0.017053	-0.001229	0.022645)
49:	387.17	0.001029	5.20	0.000830	(-0.026239	-0.011725	0.001917)
50:	392.07	0.000149	0.75	0.000119	(0.005047	0.000718	-0.009638)
51:	400.30	0.000101	0.51	0.000079	(0.003778	-0.007701	-0.002349)
52:	404.72	0.000240	1.21	0.000185	(0.012430	-0.002128	-0.005112)
53:	411.98	0.000063	0.32	0.000048	(0.003105	-0.004778	-0.003902)
54:	413.11	0.000032	0.16	0.000024	(0.003553	0.001845	0.002877)
55:	426.33	0.000221	1.12	0.000162	(-0.004276	-0.010090	0.006475)
56:	431.23	0.000221	1.12	0.000160	(-0.010540	0.003538	-0.006053)
57:	435.23	0.001470	7.43	0.001054	(-0.003857	-0.020333	0.025018)
58:	455.03	0.003256	16.45	0.002233	(0.002805	0.037825	0.028182)
59:	469.35	0.001630	8.24	0.001084	(0.017891	0.024014	-0.013678)
60:	479.66	0.002571	12.99	0.001673	(0.027020	-0.030267	-0.005152)
61:	488.87	0.004818	24.35	0.003076	(-0.055192	-0.002988	-0.004545)
62:	534.73	0.000083	0.42	0.000048	(-0.006666	0.001810	-0.000746)
63:	563.09	0.000065	0.33	0.000036	(-0.004514	0.001674	0.003579)
64:	573.87	0.001156	5.84	0.000628	(0.023229	-0.004239	0.008419)

65:	603.77	0.000175	0.88	0.000090	(0.006595	-0.006382	-0.002472)
66:	613.61	0.001826	9.23	0.000929	(-0.018764	-0.023491	-0.004978)
67:	628.66	0.004236	21.41	0.002103	(0.036758	-0.022708	-0.015363)
68:	646.65	0.001010	5.10	0.000487	(-0.004830	-0.012372	-0.017632)
69:	661.73	0.002611	13.20	0.001232	(-0.033946	-0.006286	-0.006295)
70:	692.17	0.013405	67.74	0.006044	(0.021267	-0.055996	0.049555)
71:	707.51	0.003750	18.95	0.001654	(0.006340	-0.005869	0.039739)
72:	754.63	0.004031	20.37	0.001667	(-0.031039	0.026499	-0.001213)
73:	767.74	0.004840	24.46	0.001967	(-0.036745	-0.014102	-0.020447)
74:	789.02	0.002411	12.19	0.000954	(0.027466	0.006364	0.012604)
75:	811.37	0.001125	5.68	0.000433	(0.005927	0.000263	-0.019933)
76:	811.94	0.000501	2.53	0.000193	(-0.007135	-0.007785	-0.009008)
77:	814.30	0.002655	13.42	0.001018	(-0.019036	0.025328	0.003712)
78:	818.02	0.001005	5.08	0.000383	(0.003774	0.019078	-0.002289)
79:	831.47	0.002517	12.72	0.000945	(0.012926	-0.002982	-0.027727)
80:	855.02	0.011398	57.60	0.004160	(0.002420	0.060149	0.023161)
81:	893.63	0.049374	249.51	0.017242	(-0.014363	-0.065669	0.112796)
82:	913.15	0.000054	0.27	0.000019	(0.003424	0.002254	0.001332)
83:	938.41	0.000255	1.29	0.000085	(-0.002024	0.000100	-0.008986)
84:	939.94	0.000105	0.53	0.000035	(-0.004664	-0.000633	0.003546)
85:	942.78	0.000568	2.87	0.000188	(-0.005286	0.010400	-0.007210)
86:	942.97	0.000388	1.96	0.000128	(-0.011275	-0.000944	0.000624)
87:	946.89	0.000469	2.37	0.000154	(0.010125	-0.003234	0.006443)
88:	947.12	0.000155	0.78	0.000051	(-0.005105	-0.004473	-0.002207)
89:	947.71	0.000622	3.14	0.000205	(0.006759	0.006544	-0.010784)
90:	949.27	0.000812	4.10	0.000267	(-0.005235	-0.006062	0.014237)
91:	968.71	0.000096	0.49	0.000031	(0.005190	0.001443	-0.001417)
92:	970.03	0.002695	13.62	0.000867	(0.011233	0.026953	0.003791)
93:	970.64	0.000526	2.66	0.000169	(0.006263	0.010259	0.004977)
94:	972.60	0.000917	4.63	0.000294	(-0.006714	-0.014876	-0.005266)
95:	975.06	0.000069	0.35	0.000022	(0.002469	-0.001578	0.003654)
96:	985.65	0.005091	25.73	0.001612	(-0.000924	-0.026815	0.029865)
97:	1003.23	0.000105	0.53	0.000033	(0.003482	0.004464	0.000693)
98:	1015.46	0.001506	7.61	0.000463	(-0.017300	0.010668	0.007047)
99:	1024.62	0.000562	2.84	0.000171	(-0.003592	0.010790	0.006479)
100:	1027.81	0.000209	1.06	0.000063	(0.000688	-0.007842	-0.001227)
101:	1033.91	0.000309	1.56	0.000093	(-0.004382	-0.008087	-0.002944)
102:	1035.53	0.000915	4.63	0.000276	(-0.003066	-0.011913	0.011158)
103:	1037.84	0.000450	2.27	0.000135	(-0.002110	0.000582	-0.011419)
104:	1041.07	0.000370	1.87	0.000111	(-0.005240	0.007588	-0.005083)
105:	1045.93	0.000164	0.83	0.000049	(0.004369	-0.002426	0.004889)
106:	1046.84	0.000922	4.66	0.000275	(-0.016048	0.000502	-0.004118)
107:	1102.55	0.002513	12.70	0.000711	(0.020060	-0.010818	-0.013855)
108:	1126.31	0.000669	3.38	0.000185	(0.007733	-0.007910	-0.007936)
109:	1142.92	0.000945	4.78	0.000258	(0.006447	-0.006223	-0.013333)
110:	1183.68	0.002529	12.78	0.000667	(-0.022768	-0.010783	0.005672)
111:	1192.76	0.000193	0.98	0.000051	(-0.001482	0.005714	-0.003961)
112:	1200.36	0.002482	12.54	0.000645	(-0.015839	0.019567	0.003380)
113:	1201.77	0.008187	41.37	0.002126	(-0.009071	0.044593	0.007416)
114:	1204.22	0.003302	16.68	0.000856	(0.011711	0.013187	-0.023335)
115:	1206.01	0.000930	4.70	0.000241	(-0.013320	0.003131	-0.007309)
116:	1208.60	0.000387	1.95	0.000100	(0.000826	-0.009927	-0.000758)
117:	1209.09	0.001885	9.52	0.000486	(0.010242	-0.017809	-0.008022)
118:	1212.32	0.001564	7.91	0.000403	(0.015075	0.007575	0.010865)
119:	1217.13	0.000108	0.55	0.000028	(0.002906	-0.004380	-0.000394)
120:	1219.23	0.000162	0.82	0.000042	(-0.003912	-0.004708	-0.002022)
121:	1220.70	0.000083	0.42	0.000021	(-0.002653	0.003607	-0.001136)
122:	1223.10	0.001403	7.09	0.000358	(0.009309	-0.006057	0.015320)
123:	1224.93	0.002414	12.20	0.000615	(-0.010070	-0.010819	0.019913)
124:	1225.70	0.000110	0.56	0.000028	(-0.002711	0.002858	-0.003541)
125:	1270.83	0.004086	20.65	0.001003	(0.006775	0.020808	-0.022903)
126:	1296.17	0.005844	29.53	0.001407	(0.037344	-0.000126	0.003531)
127:	1346.61	0.000356	1.80	0.000083	(0.000961	-0.006873	0.005865)
128:	1385.35	0.000232	1.17	0.000052	(0.005879	-0.002713	0.003224)

129:	1388.86	0.000723	3.65	0.000162	(-0.009734 0.006630 0.004864)
130:	1390.14	0.000791	4.00	0.000178	(0.007499 -0.003750 0.010360)
131:	1391.98	0.001306	6.60	0.000293	(-0.007276 -0.011569 0.010297)
132:	1394.30	0.000542	2.74	0.000121	(-0.006789 0.001832 -0.008471)
133:	1395.33	0.002836	14.33	0.000634	(0.020413 0.013748 -0.005337)
134:	1397.24	0.002730	13.80	0.000610	(0.020827 -0.002630 0.013001)
135:	1399.37	0.001058	5.35	0.000236	(-0.013051 -0.007704 0.002491)
136:	1422.93	0.000577	2.92	0.000127	(0.002570 -0.009565 -0.005340)
137:	1425.85	0.005090	25.73	0.001114	(0.004312 0.026659 0.019617)
138:	1428.29	0.002167	10.95	0.000473	(0.012949 -0.006654 -0.016169)
139:	1429.74	0.000682	3.45	0.000149	(-0.000028 -0.010969 0.005339)
140:	1436.01	0.001593	8.05	0.000346	(0.009615 -0.009958 0.012436)
141:	1436.75	0.002295	11.60	0.000499	(0.004646 -0.015142 0.015738)
142:	1457.34	0.001793	9.06	0.000384	(0.019453 -0.001287 0.001979)
143:	1463.02	0.000244	1.23	0.000052	(-0.003359 -0.001394 0.006230)
144:	1468.20	0.000070	0.36	0.000015	(-0.000956 -0.000813 -0.003657)
145:	1469.61	0.000213	1.07	0.000045	(-0.002028 -0.005963 -0.002343)
146:	1470.44	0.000290	1.47	0.000062	(-0.006358 0.002140 -0.004076)
147:	1473.12	0.000913	4.61	0.000193	(0.006692 -0.007532 0.009586)
148:	1476.12	0.000909	4.59	0.000192	(0.007080 0.003748 -0.011310)
149:	1477.35	0.000805	4.07	0.000170	(-0.010607 -0.000454 -0.007570)
150:	1478.84	0.000151	0.77	0.000032	(0.000102 -0.004995 0.002647)
151:	1482.24	0.002105	10.64	0.000443	(-0.010263 -0.014728 0.010994)
152:	1483.18	0.001172	5.92	0.000247	(-0.011214 0.008986 -0.006327)
153:	1485.12	0.002742	13.86	0.000576	(0.002109 -0.010169 0.021641)
154:	1485.46	0.000495	2.50	0.000104	(0.007476 0.006791 -0.001418)
155:	1485.99	0.008138	41.13	0.001709	(0.004629 0.027040 -0.030927)
156:	1494.76	0.000434	2.19	0.000091	(-0.001547 0.006059 0.007180)
157:	1497.01	0.001258	6.36	0.000262	(0.009525 0.002364 -0.012881)
158:	1498.08	0.002349	11.87	0.000489	(0.016374 0.006232 -0.013506)
159:	1498.78	0.000894	4.52	0.000186	(-0.005957 -0.008453 -0.008895)
160:	1501.66	0.000732	3.70	0.000152	(0.007216 0.002083 -0.009786)
161:	1504.15	0.004439	22.43	0.000921	(-0.014030 0.004261 -0.026571)
162:	1509.32	0.001614	8.16	0.000334	(-0.014381 0.004237 -0.010438)
163:	1511.01	0.002624	13.26	0.000542	(-0.017544 -0.015302 0.000189)
164:	1516.50	0.005973	30.18	0.001229	(-0.020268 0.025196 0.013543)
165:	1519.48	0.000178	0.90	0.000037	(0.002231 -0.004356 0.003559)
166:	1519.90	0.002721	13.75	0.000559	(0.000683 -0.022241 0.007973)
167:	1521.92	0.003910	19.76	0.000802	(-0.005969 -0.022294 -0.016402)
168:	1607.40	0.007096	35.86	0.001378	(0.003430 -0.025639 0.026618)
169:	1619.64	0.014096	71.23	0.002716	(0.048832 -0.007831 -0.016432)
170:	3014.47	0.002521	12.74	0.000261	(0.000277 -0.008476 0.013750)
171:	3015.40	0.003223	16.29	0.000334	(0.011734 -0.009862 -0.009930)
172:	3022.03	0.002336	11.81	0.000241	(-0.000285 0.013329 -0.007968)
173:	3023.57	0.001837	9.28	0.000190	(-0.010827 -0.008428 0.001134)
174:	3024.08	0.003389	17.13	0.000350	(-0.016241 -0.000151 0.009272)
175:	3025.54	0.005673	28.67	0.000585	(0.012926 0.014207 0.014704)
176:	3026.13	0.000603	3.05	0.000062	(-0.001440 -0.001207 0.007661)
177:	3027.62	0.001824	9.22	0.000188	(0.012963 -0.004406 0.000757)
178:	3030.58	0.004695	23.72	0.000483	(-0.016438 0.009936 0.010700)
179:	3031.19	0.006450	32.60	0.000664	(-0.019914 -0.003807 -0.015905)
180:	3033.51	0.002237	11.31	0.000230	(-0.001986 0.012365 -0.008561)
181:	3034.16	0.003380	17.08	0.000348	(0.002434 0.015779 -0.009631)
182:	3046.14	0.000755	3.81	0.000077	(0.002463 -0.006849 0.004934)
183:	3074.81	0.000206	1.04	0.000021	(0.002032 -0.003177 0.002577)
184:	3088.91	0.002438	12.32	0.000246	(-0.014383 0.004158 0.004708)
185:	3089.23	0.002440	12.33	0.000246	(0.006231 0.014358 -0.001218)
186:	3096.20	0.001273	6.43	0.000128	(0.005087 0.009167 0.004287)
187:	3100.41	0.002151	10.87	0.000216	(0.004685 -0.012583 -0.006017)
188:	3100.60	0.001190	6.01	0.000120	(0.007522 -0.002146 0.007652)
189:	3102.10	0.002461	12.44	0.000248	(0.013950 -0.007221 -0.000886)
190:	3104.77	0.001063	5.37	0.000107	(0.009490 -0.002227 0.003438)
191:	3105.28	0.003608	18.23	0.000363	(-0.016415 -0.007029 -0.006612)
192:	3106.03	0.002332	11.79	0.000234	(0.004891 0.013589 0.005072)

193:	3107.05	0.000709	3.58	0.000071	(-0.003439	-0.007501	0.001777)
194:	3108.78	0.002870	14.50	0.000288	(0.015628	-0.006620	0.000112)
195:	3114.79	0.001009	5.10	0.000101	(-0.006967	-0.006992	0.001909)
196:	3115.14	0.000478	2.41	0.000048	(-0.006837	0.001051	0.000130)
197:	3115.88	0.002092	10.57	0.000209	(-0.008221	-0.006271	-0.010128)
198:	3123.24	0.001911	9.66	0.000191	(0.011734	-0.004599	-0.005663)
199:	3128.14	0.001578	7.97	0.000157	(-0.006168	0.004451	-0.009978)
200:	3128.53	0.000982	4.96	0.000098	(0.001110	0.003861	-0.009047)
201:	3133.12	0.001268	6.41	0.000126	(-0.000877	0.003784	-0.010548)
202:	3134.54	0.001013	5.12	0.000101	(-0.006919	-0.000617	-0.007256)
203:	3137.11	0.001646	8.32	0.000164	(-0.006819	-0.005925	0.009060)
204:	3138.27	0.000674	3.40	0.000067	(-0.005550	0.001563	-0.005807)
205:	3139.46	0.001926	9.73	0.000191	(-0.005259	-0.000677	-0.012778)
206:	3139.95	0.001017	5.14	0.000101	(0.006669	-0.000058	-0.007523)
207:	3144.36	0.000737	3.72	0.000073	(0.007721	-0.001270	-0.003448)
208:	3144.66	0.000562	2.84	0.000056	(0.005749	-0.004474	-0.001636)
209:	3145.63	0.001785	9.02	0.000177	(0.005630	0.011000	-0.004934)
210:	3179.65	0.000104	0.53	0.000010	(0.002333	-0.002172	-0.000308)
211:	3189.59	0.000833	4.21	0.000081	(-0.000632	-0.005979	0.006732)
212:	3199.90	0.000009	0.05	0.000001	(-0.000566	0.000742	-0.000019)

Computed Raman spectrum of [PNOP^{tBu}]$^+$ at the RI-r^{2}SCAN-3c(D4)/def2-mTZVPP level of theory

Mode	freq (cm**-1)	Activity	Depolarization
6:	26.21	0.462796	0.733687
7:	49.39	0.082863	0.457793
8:	64.76	1.693407	0.707718
9:	68.83	1.584777	0.722480
10:	81.81	0.655122	0.439234
11:	101.84	0.498263	0.721498
12:	105.98	1.760053	0.712308
13:	115.95	2.274363	0.697373
14:	118.54	1.747282	0.350503
15:	131.54	2.076240	0.631201
16:	146.42	0.408145	0.641118
17:	155.53	3.521812	0.538134
18:	175.12	1.448754	0.749696
19:	183.23	0.760558	0.715740
20:	197.44	0.921083	0.628065
21:	200.79	0.937478	0.330386
22:	204.61	0.772269	0.573666
23:	215.40	1.297743	0.083932
24:	220.92	0.536404	0.298803
25:	228.46	0.545622	0.554500
26:	236.58	1.074044	0.242547
27:	242.10	1.709086	0.223008
28:	243.35	0.130711	0.660194
29:	252.41	5.092484	0.221448
30:	256.09	3.120320	0.143440
31:	261.75	1.097590	0.740094
32:	266.21	6.576908	0.093554
33:	278.71	2.713993	0.257600
34:	282.65	1.198734	0.295528
35:	286.40	3.306460	0.055848
36:	291.13	0.604046	0.369625
37:	295.48	0.778346	0.579775
38:	305.36	3.101894	0.171623
39:	318.05	2.043591	0.493186
40:	323.12	0.273195	0.644746
41:	329.93	0.983386	0.749332
42:	333.97	0.189655	0.550013
43:	337.50	0.487694	0.645320
44:	340.35	0.682757	0.729284
45:	351.25	0.429045	0.418997
46:	359.12	0.824306	0.512371
47:	361.93	1.660791	0.238991
48:	369.35	1.005543	0.749746
49:	387.17	0.293013	0.434312
50:	392.07	0.663663	0.304912
51:	400.30	0.170485	0.577673
52:	404.72	0.275420	0.674732
53:	411.98	0.370637	0.743902
54:	413.11	0.418894	0.713554
55:	426.33	1.093028	0.456065
56:	431.23	3.326356	0.111553
57:	435.23	0.239963	0.582804
58:	455.03	1.049233	0.437466
59:	469.35	1.582423	0.509196
60:	479.66	4.832193	0.201458
61:	488.87	12.589461	0.113501
62:	534.73	2.151680	0.696788
63:	563.09	2.710888	0.170767
64:	573.87	34.981015	0.020852
65:	603.77	6.333431	0.400975
66:	613.61	7.660043	0.584567
67:	628.66	2.097728	0.625479
68:	646.65	3.070131	0.298523
69:	661.73	6.838203	0.107779
70:	692.17	12.257903	0.464638
71:	707.51	2.787473	0.191218
72:	754.63	2.080808	0.260850
73:	767.74	3.876337	0.120665
74:	789.02	3.466935	0.181091
75:	811.37	3.305692	0.749907
76:	811.94	6.222161	0.545561
77:	814.30	6.825026	0.662084
78:	818.02	6.552387	0.618531
79:	831.47	3.270460	0.222216
80:	855.02	3.927337	0.255046
81:	893.63	4.423399	0.418095
82:	913.15	3.726646	0.600641
83:	938.41	1.847017	0.732927
84:	939.94	1.968471	0.696834
85:	942.78	2.383211	0.742442
86:	942.97	1.303761	0.734106
87:	946.89	4.680109	0.732410
88:	947.12	1.814392	0.719572
89:	947.71	0.104226	0.431434
90:	949.27	2.720165	0.739830
91:	968.71	0.511082	0.723706
92:	970.03	8.828879	0.694078
93:	970.64	2.028657	0.514278
94:	972.60	3.374106	0.640010
95:	975.06	0.388770	0.749763
96:	985.65	2.064837	0.749998
97:	1003.23	0.625218	0.085314
98:	1015.46	30.080287	0.082513
99:	1024.62	1.034006	0.635568
100:	1027.81	1.173564	0.717310
101:	1033.91	1.487502	0.749928
102:	1035.53	1.958924	0.576839
103:	1037.84	0.732883	0.601561
104:	1041.07	2.171356	0.681785
105:	1045.93	2.719940	0.682414
106:	1046.84	0.499189	0.688875
107:	1102.55	13.167953	0.115733
108:	1126.31	7.882408	0.529775
109:	1142.92	12.072113	0.344696
110:	1183.68	10.574296	0.526397
111:	1192.76	2.353335	0.741691
112:	1200.36	9.172610	0.587453
113:	1201.77	6.201415	0.692726
114:	1204.22	4.426881	0.144678
115:	1206.01	11.361460	0.027946
116:	1208.60	0.558856	0.503998
117:	1209.09	1.623336	0.039372
118:	1212.32	4.508460	0.251662
119:	1217.13	2.332296	0.697783
120:	1219.23	1.184070	0.633820
121:	1220.70	1.642995	0.634394
122:	1223.10	4.556142	0.728454
123:	1224.93	1.721214	0.345460
124:	1225.70	5.380568	0.449064
125:	1270.83	5.140097	0.627520
126:	1296.17	54.402741	0.144310
127:	1346.61	3.605174	0.746715
128:	1385.35	0.322053	0.749773
129:	1388.86	0.715446	0.743658
130:	1390.14	0.966789	0.680006
131:	1391.98	1.615576	0.748987
132:	1394.30	0.755040	0.726317
133:	1395.33	0.707125	0.729132
134:	1397.24	0.545124	0.669942
135:	1399.37	1.947176	0.661650
136:	1422.93	0.420560	0.594851
137:	1425.85	2.397423	0.636002
138:	1428.29	1.699189	0.639010
139:	1429.74	1.241664	0.749456
140:	1436.01	1.387639	0.525879
141:	1436.75	3.781317	0.749930
142:	1457.34	1.726035	0.439280
143:	1463.02	3.417692	0.739000
144:	1468.20	1.830068	0.741730
145:	1469.61	1.922645	0.692326
146:	1470.44	3.107146	0.659857
147:	1473.12	5.013522	0.744412
148:	1476.12	6.652516	0.739520
149:	1477.35	4.905214	0.742230
150:	1478.84	9.631008	0.748847
151:	1482.24	2.277308	0.709354
152:	1483.18	8.919417	0.696461
153:	1485.12	1.829133	0.631744
154:	1485.46	2.859940	0.643418
155:	1485.99	1.371249	0.747827
156:	1494.76	11.240413	0.747720
157:	1497.01	6.204282	0.742793
158:	1498.08	6.498141	0.698454
159:	1498.78	6.774706	0.749850
160:	1501.66	4.905929	0.705618
161:	1504.15	2.772125	0.658166
162:	1509.32	14.287092	0.735262
163:	1511.01	16.394012	0.742548
164:	1516.50	2.471183	0.681625

165:	1519.48	0.544212	0.468678
166:	1519.90	0.897417	0.707696
167:	1521.92	0.038269	0.218653
168:	1607.40	31.735455	0.749999
169:	1619.64	9.637870	0.230818
170:	3014.47	150.292526	0.049433
171:	3015.40	81.673996	0.132877
172:	3022.03	26.632885	0.099725
173:	3023.57	144.342347	0.058839
174:	3024.08	121.190497	0.071011
175:	3025.54	77.142973	0.125104
176:	3026.13	5.644092	0.662186
177:	3027.62	115.269028	0.092552
178:	3030.58	150.967006	0.121564
179:	3031.19	346.868311	0.029381
180:	3033.51	294.780164	0.054492
181:	3034.16	172.552725	0.066551
182:	3046.14	172.948844	0.048302
183:	3074.81	118.107907	0.055618
184:	3088.91	67.413775	0.565686
185:	3089.23	42.637153	0.706047
186:	3096.20	51.477631	0.615970
187:	3100.41	47.185294	0.718909
188:	3100.60	38.640273	0.630986
189:	3102.10	44.994768	0.718985
190:	3104.77	28.151720	0.693401
191:	3105.28	72.951959	0.725600
192:	3106.03	59.909013	0.700350
193:	3107.05	42.234915	0.746985
194:	3108.78	107.864291	0.675628
195:	3114.79	17.290795	0.734555
196:	3115.14	40.007022	0.711621
197:	3115.88	48.283239	0.700722
198:	3123.24	28.607304	0.743108
199:	3128.14	17.492003	0.617330
200:	3128.53	16.150245	0.703077
201:	3133.12	9.222674	0.749699
202:	3134.54	49.843776	0.691672
203:	3137.11	8.878572	0.737459
204:	3138.27	36.142382	0.747339
205:	3139.46	25.891212	0.734571
206:	3139.95	19.070818	0.556584
207:	3144.36	46.959158	0.742991
208:	3144.66	28.732352	0.748514
209:	3145.63	27.759780	0.749997
210:	3179.65	49.940619	0.711292
211:	3189.59	58.683940	0.730084
212:	3199.90	218.377799	0.146924

7. References

[1] A. Decken, H. D. B. Jenkins, G. B. Nikiforov, J. Passmore, *Dalton trans.* **2004**, 2496.

[2] M. Kawatsura, J. F. Hartwig, *Organometallics* **2001**, *20*, 1960.

[3] G. R. Fulmer, A. J. M. Miller, N. H. Sherden, H. E. Gottlieb, A. Nudelman, B. M. Stoltz, J. E. Bercaw, K. I. Goldberg, *Organometallics* **2010**, *29*, 2176.

[4] R. K. Harris, E. D. Becker, S. M. Cabral de Menezes, R. Goodfellow, P. Granger, *Magn. Reson. Chem.* **2002**, *40*, 489.

[5] J. Cosier, A. M. Glazer, *J. Appl. Crystallogr.* **1986**, *19*, 105.

[6] a) Bruker, *SAINT, V 8.40B*. Bruker AXS Inc., Madison, Wisconsin, USA; b) Bruker, *TWINABS*. Bruker AXS Inc., Madison, Wisconsin, USA.

[7] G. M. Sheldrick, *Acta Crystallogr., Sect. A: Found. Crystallogr.* **2015**, *71*, 3.

[8] G. M. Sheldrick, *Acta Crystallogr., Sect. C: Cryst. Struct. Commun.* **2015**, *71*, 3.

[9] C. B. Hübschle, G. M. Sheldrick, B. Dittrich, *J. Appl. Crystallogr.* **2011**, *44*, 1281.

[10] a) D. Kratzert, J. J. Holstein, I. Krossing, *J. Appl. Crystallogr.* **2015**, *48*, 933; b) D. Kratzert, I. Krossing, *J. Appl. Crystallogr.* **2018**, *51*, 928.

[11] D. Kratzert, *FinalCif, V73*. https://www.xs3.uni-freiburg.de/research/finalcif.

[12] C. R. Groom, I. J. Bruno, M. P. Lightfoot, S. C. Ward, *Acta Crystallogr., Sect. B: Struct. Sci* **2016**, *72*, 171.

[13] F. Neese, F. Wennmohs, U. Becker, C. Riplinger, *J. Chem. Phys.* **2020**, *152*, 224108.

[14] S. Grimme, A. Hansen, S. Ehlert, J.-M. Mewes, *J. Chem. Phys.* **2021**, *154*, 64103.

[15] a) F. Weigend, R. Ahlrichs, *Phys. Chem. Chem. Phys.* **2005**, *7*, 3297; b) F. Weigend, *Phys. Chem. Chem. Phys.* **2006**, *8*, 1057.

[16] a) M. Sierka, A. Hogekamp, R. Ahlrichs, *J. Chem. Phys.* **2003**, *118*, 9136; b) R. Ahlrichs, *Phys. Chem. Chem. Phys.* **2004**, *6*, 5119.

[17] E. Caldeweyher, C. Bannwarth, S. Grimme, *J. Chem. Phys.* **2017**, *147*, 34112.

[18] V. Ásgeirsson, B. O. Birgisson, R. Bjornsson, U. Becker, F. Neese, C. Riplinger, H. Jónsson, *J. Chem. Theory Comput.* **2021**, *17*, 4929.

[19] K. Ishida, K. Morokuma, A. Komornicki, *J. Chem. Phys.* **1977**, *66*, 2153.

[20] I. Raabe, K. Wagner, K. Guttsche, M. Wang, M. Grätzel, G. Santiso-Quiñones, I. Krossing, *Chem. Eur. J.* **2009**, *15*, 1966.
